# Bapineuzumab for mild to moderate Alzheimer’s disease in two global, randomized, phase 3 trials

**DOI:** 10.1186/s13195-016-0189-7

**Published:** 2016-05-12

**Authors:** Rik Vandenberghe, Juha O. Rinne, Mercè Boada, Sadao Katayama, Philip Scheltens, Bruno Vellas, Michael Tuchman, Achim Gass, Jochen B. Fiebach, Derek Hill, Kasia Lobello, David Li, Tom McRae, Prisca Lucas, Iona Evans, Kevin Booth, Gerald Luscan, Bradley T. Wyman, Lisa Hua, Lingfeng Yang, H. Robert Brashear, Ronald S. Black, for the Bapineuzumab 3000 and 3001 Clinical Study Investigators

**Affiliations:** University Hospitals Leuven, Department of Neurosciences, Alzheimer Research Centre KU Leuven, Herestraat 49, 3000, Leuven, Belgium; Turku PET Centre and Division of Clinical Neurosciences, University of Turku and Turku University Hospital, Kiinamyllynkatu 4-8, 20520 Turku, Finland; Fundació ACE, Barcelona Alzheimer Treatment and Research Center, Gran via de Carles III, 85 Bis, 08028 Barcelona, Spain; Graduate School of Biomedical Sciences, Hiroshima University, 1-2-3 Kasumi, Minami-ku, Hiroshima 734-8551 Japan; Alzheimercentrum VUmc, Neurology, VU University Medical Center, PO Box 7057, 1007 MB Amsterdam, Netherlands; CHU Toulouse, Gérontopôle, 170 Avenue de Casselardit, TSA 40031, 31059 Toulouse, Cedex 9 France; Palm Beach Neurological Center, 3365 Burns Road, Suite 203, Palm Beach Gardens, FL 33410 USA; Department of Neurology, University Hospital Mannheim, University Medical Centre Mannheim, Theodor-Kutzer-Ufer 1-3, 68167 Mannheim, Germany; Center for Stroke Research Berlin (CSB), Charité Universitätsmedizin Berlin, Hindenburgdamm 30, 12200 Berlin, Germany; IXICO Ltd., The London Bioscience Innovation Centre, 4th Floor, Griffin Court, 15 Long Lane, London, EC1A 9PN UK; Pfizer Inc., 500 Arcola Road, Collegeville, PA 19426 USA; Pfizer Inc., 235 East 42nd Street, New York, NY 10017 USA; Pfizer Global Research and Development (PGRD), 23-25 avenue Du Docteur Lannelongue, Paris, Île-De-France 75014 France; Pfizer Ltd., Walton Oaks, Dorking Road, Tadworth, Surrey KT20 7NS UK; Pfizer Inc., 7 Kings Highway, Groton, CT 06340 USA; Janssen Alzheimer Immunotherapy Research & Development, LLC, 700 Gateway Boulevard, South San Francisco, CA 94080 USA

**Keywords:** Alzheimer’s disease, Bapineuzumab, Immunotherapy, Amyloid β, Clinical trial, ARIA-E, Vasogenic edema

## Abstract

**Background:**

Our objective was to evaluate the efficacy (clinical and biomarker) and safety of intravenous bapineuzumab in patients with mild to moderate Alzheimer’s disease (AD).

**Methods:**

Two of four phase 3, multicenter, randomized, double-blind, placebo-controlled, 18-month trials were conducted globally: one in apolipoprotein E ε4 carriers and another in noncarriers. Patients received bapineuzumab 0.5 mg/kg (both trials) or 1.0 mg/kg (noncarrier trial) or placebo every 13 weeks. Coprimary endpoints were change from baseline to week 78 on the 11-item Alzheimer’s Disease Assessment Scale–Cognitive subscale and the Disability Assessment for Dementia.

**Results:**

A total of 683 and 329 patients completed the current carrier and noncarrier trials, respectively, which were terminated prematurely owing to lack of efficacy in the two other phase 3 trials of bapineuzumab in AD. The current trials showed no significant difference between bapineuzumab and placebo for the coprimary endpoints and no effect of bapineuzumab on amyloid load or cerebrospinal fluid phosphorylated tau. (Both measures were stable over time in the placebo group.) Amyloid-related imaging abnormalities with edema or effusion were confirmed as the most notable adverse event.

**Conclusions:**

These phase 3 global trials confirmed lack of efficacy of bapineuzumab at tested doses on clinical endpoints in patients with mild to moderate AD. Some differences in the biomarker results were seen compared with the other phase 3 bapineuzumab trials. No unexpected adverse events were observed.

**Trial registration:**

Noncarriers (3000) ClinicalTrials.gov identifier NCT00667810; registered 24 Apr 2008.

Carriers (3001) ClinicalTrials.gov identifier NCT00676143; registered 2 May 2008.

**Electronic supplementary material:**

The online version of this article (doi:10.1186/s13195-016-0189-7) contains supplementary material, which is available to authorized users.

## Background

Immunotherapy with monoclonal antibodies has been under investigation as a therapeutic approach to Alzheimer’s disease (AD) [1, 2]. Bapineuzumab is a monoclonal antibody specific to the N-terminus of the amyloid β (Aβ) protein designed to decrease plaque formation and promote clearance of Aβ [2–4]. In phase 2 studies in patients with mild to moderate AD, bapineuzumab reduced phosphorylated tau (p-tau) protein in cerebrospinal fluid (CSF) and ^11^C-Pittsburgh compound B (PiB) average uptake visualized by positron emission tomography (PET) [5, 6]. The findings provided a rationale for conducting separate trials in apolipoprotein E (ApoE) ε4 allele carriers and noncarriers and for limiting the bapineuzumab dose in carriers to minimize risk of amyloid-related imaging abnormalities with edema or effusion (ARIA-E; previously termed *vasogenic edema*) [7].

The bapineuzumab phase 3 development program consisted of four nearly identical phase 3 trials conducted in parallel to evaluate the efficacy and safety of intravenous (IV) bapineuzumab over 18 months in patients with mild to moderate AD. The other two trials reported elsewhere were conducted primarily in the United States: Study 302 in ApoE ε4 allele carriers (ClinicalTrials.gov identifier NCT00575055) and Study 301 in noncarriers (ClinicalTrials.gov identifier NCT00574132) [3]. These studies showed no benefit of bapineuzumab on the cognitive or functional endpoints assessed.

Here we report results from the two global phase 3 trials of IV bapineuzumab in ApoE ε4 carriers (Study 3001, ClinicalTrials.gov identifier NCT00676143) and noncarriers (Study 3000, ClinicalTrials.gov identifier NCT00667810). Both trials were terminated prematurely because of lack of clinical efficacy observed in the 301 and 302 studies [3].

## Methods

### Study design

Studies 3000 and 3001 were multicenter, randomized, double-blind, placebo-controlled, 18-month clinical trials in which investigators evaluated the efficacy and safety of bapineuzumab 0.5 mg/kg versus placebo (ratio 3:2) in ApoE ε4 carriers (Study 3001) and bapineuzumab 0.5 mg/kg, 1.0 mg/kg, or placebo (ratio 3:3:4) in ApoE ε4 noncarriers (Study 3000). Originally, Study 3000 included a bapineuzumab 2.0 mg/kg dose, which was discontinued because of a high rate of clinically symptomatic ARIA-E. Patients randomized to the 2.0 mg/kg group were reassigned to receive 1.0 mg/kg for the remainder of the study, and the randomization ratio was adjusted accordingly. Allocation of patients to treatment groups using stratified block randomization proceeded through the entering of subject information by the study coordinator or delegate and the dispenser (unblinded pharmacist) into an interactive voice/web response system. The dispenser was then provided with a subject randomization number and treatment assignment, and a confirmatory facsimile was sent to the dispenser. Randomization was stratified by Mini Mental State Examination (MMSE) scores (16–21; 22–26); concomitant cholinesterase inhibitor and/or memantine use; substudy participation; and, in the carrier study, number of copies of ApoE ε4 allele (one allele; two alleles). Patients received a total of six IV infusions of bapineuzumab or placebo every 13 weeks, with brain magnetic resonance imaging (MRI) monitoring for ARIA-E conducted at 6 weeks after each infusion. There were three biomarker substudies: a brain amyloid PET substudy, a CSF substudy, and a volumetric magnetic resonance imaging (vMRI) substudy. No interim analyses were planned or performed.

### Inclusion criteria

Patients were eligible for enrollment if they were aged 50–88 years with a diagnosis of probable AD and had an MMSE score of 16–26, inclusive, and a screening MRI scan consistent with AD. Inclusion and exclusion criteria were similar to those used in the 301/302 studies [3] and are listed in Additional file 2.

### Outcome measures

Coprimary efficacy endpoints were change from baseline to week 78 in 11-item Alzheimer’s Disease Assessment Scale–Cognitive subscale (ADAS-Cog/11) score and Disability Assessment for Dementia (DAD) total score. Change from baseline to week 78 in Dependence Scale, Clinical Dementia Rating–Sum of Boxes (CDR-SOB), and Neuropsychological Test Battery (NTB) total Z-scores were additional endpoints. Prespecified secondary biomarker endpoints included change from baseline to week 71 in PiB-PET global cortical average (GCA) standardized uptake value ratio (SUVr) of five cortical regions of interest (frontal, lateral temporal, parietal, anterior cingulate, posterior cingulate/precuneus), CSF p-tau, and brain volume assessed by brain boundary shift integral (BBSI) on vMRI. PiB-PET and vMRI were read centrally. Human plasma Aβ_*x*–40_ peptide levels were measured using a validated electrochemiluminescence immunoassay method on a Meso Scale Discovery (Rockville, MD, USA) platform, which had a range of quantification from 50 to 3200 pg/ml in 100 % matrix with a minimum required sample dilution of 1:2 and maximum sample dilution of 1:64. This platform had an interassay precision of 9 % and an intraassay precision of 5 %. CSF ^181^phospho-tau concentrations were measured using the INNOTEST enzyme-linked immunosorbent assay (ELISA) (Innogenetics, Ghent, Belgium), which had an interassay precision of 3–6 % and an intraassay precision of 2–5 %. Samples were tested pairwise per patient to reduce between-assay run variability (i.e., baseline and postbaseline samples in the same assay run and on the same microtiter ELISA plate). All biosamples were analyzed in a centralized location at the Janssen Alzheimer Immunotherapy Research & Development Bioanalytical Development laboratory in South San Francisco, CA, USA. All prespecified endpoints are listed in Additional file 3.

### Statistical methods

For the ApoE ε4 carrier study, approximately 480 patients were to be randomized to bapineuzumab 0.5 mg/kg and 320 to placebo. This number of patients gave 90 % power to detect a 2.21-point advantage for the bapineuzumab group over placebo on ADAS-Cog/11 total score and a 5.39-point advantage on DAD total score at week 78. For the ApoE ε4 noncarrier study, approximately 295 patients were to be randomized to each bapineuzumab dose group (0.5 and 1.0 mg/kg) and 400 patients to placebo, giving 90 % power to detect a 2.65-point advantage on ADAS-Cog/11 total score for a bapineuzumab dose over placebo and a 6.56-point advantage on DAD total score at week 78. The standard deviations for the ADAS-Cog/11 total score and the DAD total score used in the power calculation were 9.3 and 23.0, respectively (based on previous studies). Planned substudy enrollment in the carrier substudies was 80 patients for the PiB-PET substudy, 300 for the CSF substudy, and 680 for the vMRI substudy. In the noncarrier study, planned enrollment was 90 patients for the PiB-PET substudy, 190 for the CSF substudy, and 550 for the vMRI substudy.

### Study populations

Study populations included *Safety*: all randomized patients who received at least one infusion or portion of an infusion of study drug; *Modified intention-to-treat (mITT): * subjects in the Safety population who had a baseline assessment and at least one postbaseline assessment of ADAS-Cog/11 and DAD total scores; *All PiB-PET, CSF, and vMRI:* subjects in the Safety population who were enrolled in the specified substudy and had a valid baseline assessment and at least one postbaseline measurement; *PiB-PET:* patients in the All PiB-PET population who had a baseline SUVr ≥1.35, the threshold for amyloid positivity, and had at least one postbaseline measurement. A restricted maximum likelihood-based mixed model for repeated measures (MMRM) was used to analyze the coprimary efficacy endpoints. Primary analysis was based on treatment difference using least squares means, with factor levels weighted according to overall baseline sample proportions. CSF biomarkers were analyzed using analysis of covariance, since week 71 was the only postbaseline assessment.

## Results

### Patient disposition

**Fig. 1:**
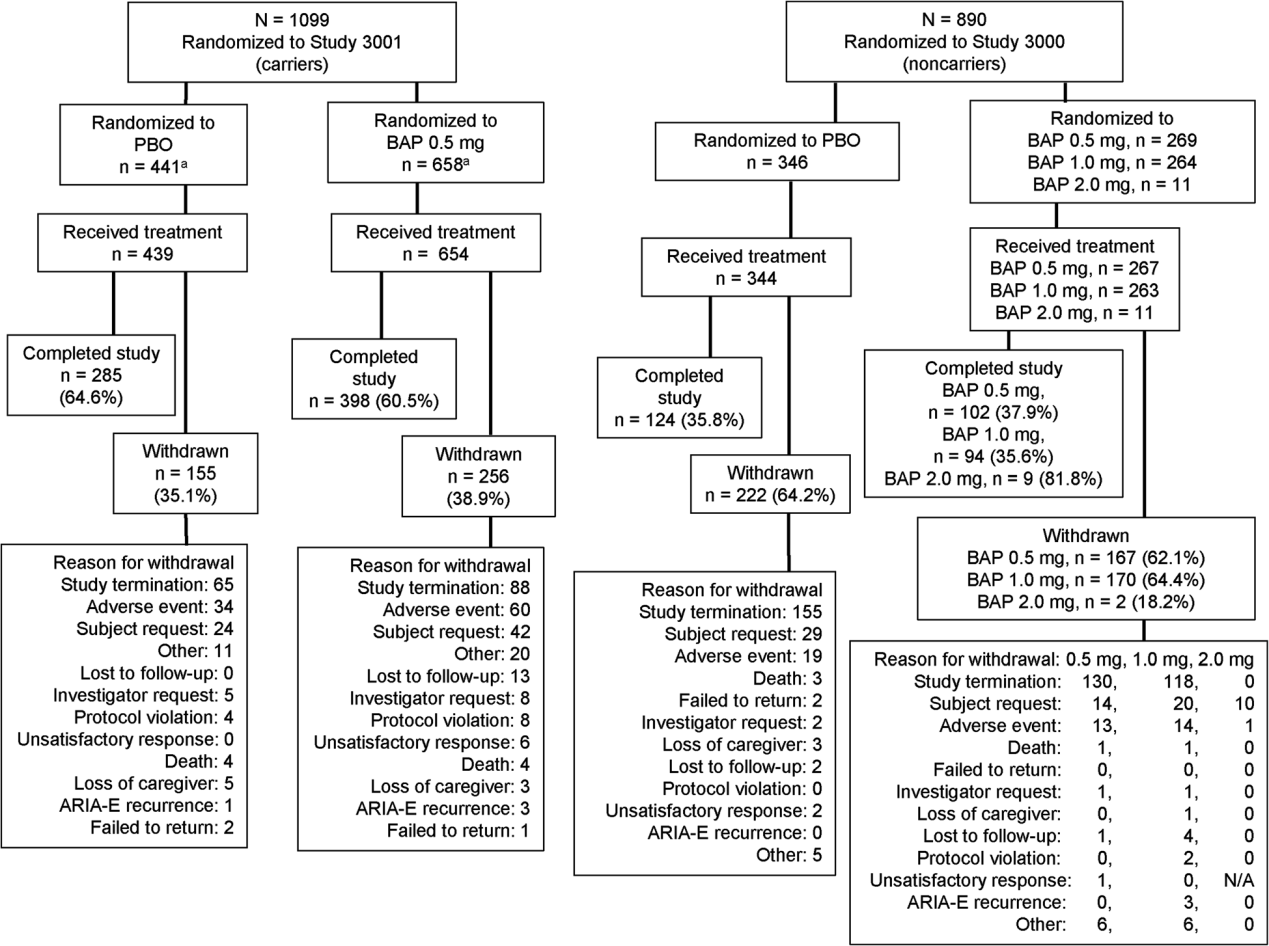
Disposition of patients with Alzheimer’s disease in the apolipoprotein E ε4 carrier and noncarrier studies. Recruitment and follow-up occurred between 28 May 2008 and 3 December 2012 for the carrier study and between 25 June 2008 and 27 November 2012 for the noncarrier study. ARIA-E, amyloid-related imaging abnormalities with edema or effusion; BAP, bapineuzumab; N/A, not applicable; PBO, placebo. ^a^Subject participation status is unknown for five subjects (one in PBO group, four in BAP group) owing to missing conclusion of patient participation in study and/or conclusion of patient participation in treatment electronic case report form pages. Four of these subjects completed six infusions and the week 78 visit. One subject completed four infusions and the week 45 visit

In the ApoE ε4 carrier study, 1099 patients were randomized and 1093 were treated (654 bapineuzumab 0.5 mg/kg, 439 placebo) (Fig. 1). A total of 1081 patients were included in the mITT population (650 bapineuzumab, 431 placebo). Three hundred ninety-eight treated patients (60.9 %) in the bapineuzumab group and 285 (64.9 %) in the placebo group completed the study (60.5 % and 64.6 % of randomized subjects, respectively) (Fig. 1). The most common reason for discontinuation was study termination by the sponsor (13.5 % bapineuzumab, 14.8 % placebo). Withdrawal due to adverse events (AEs) was higher for the bapineuzumab group (9.0 %) than for the placebo group (7.3 %) (Fig. 1).

In the ApoE ε4 noncarrier study, 890 patients were randomized with 885 treated (267 bapineuzumab 0.5 mg/kg, 263 bapineuzumab 1.0 mg/kg, 11 bapineuzumab 2.0 mg/kg, 344 placebo) (Fig. 1). The mITT population included 847 patients (255 bapineuzumab 0.5 mg/kg, 253 bapineuzumab 1.0 mg/kg, 11 bapineuzumab 2.0 mg/kg, 328 placebo). Patients in the 2.0 mg/kg group were not included in the primary efficacy analysis or safety analysis. Three hundred twenty-nine treated patients (37.2 %) completed the study (102 [38.2 %], 94 [35.7 %], 9 [81.8 %], and 124 [36.0 %] in the bapineuzumab 0.5 mg/kg, 1.0 mg/kg, 2.0 mg/kg, and placebo groups, respectively). A total of 556 treated patients withdrew, with the most common reason being sponsor decision to terminate the study (48.3 %, 44.9 %, and 45.1 % in the bapineuzumab 0.5 mg/kg, 1.0 mg/kg, and placebo groups, respectively); withdrawal due to AEs was comparable across treatment groups (4.9 %, 4.9 %, and 5.5 %, respectively) (Fig. 1).

### Exposure

In the carrier study safety population, the median duration of exposure was 1.49 years in both groups. All six infusions were administered to 57.4 % of patients in the bapineuzumab 0.5 mg/kg group and 69.8 % in the placebo group. In the noncarrier study safety population, the median duration of exposure was 1 year in all groups. All six infusions were administered to 41.6 %, 38.3 %, and 40.9 % in the bapineuzumab 0.5 mg/kg, bapineuzumab 1.0 mg/kg, and placebo groups, respectively, in the mITT population (Table 1). Among the 11 patients in the 2.0 mg/kg group, the median duration of exposure was 1 year and all six infusions were received by 54.5 % of patients in the mITT population.

**Table 1:** Patient demographics and baseline characteristics (modified intention-to-treat population)

	ApoE ε4 carrier study		ApoE ε4 noncarrier study		
	Placebo (*n* = 431)	BAP 0.5 (*n* = 650)	Placebo (*n* = 328)	BAP 0.5 (*n* = 255)	BAP 1.0 (*n* = 253)
Mean age, years	70.2	70.9	69.7	71.1	70.7
Female, %	60.1	64.5	57.9	55.7	57.3
White, %	82.6	79.5	80.5	79.2	79.4
Asian, %	16.0	17.7	17.1	17.3	17.4
Black, %	0.7	0.8	0.6	0.8	2.0
Other, %	0.7	2.0	1.8	2.7	1.2
Mean duration of AD, years (SD)	2.9 (2.2)	3.0 (2.2)	2.8 (2.5)	2.6 (2.3)	2.9 (2.2)
ApoE ε4 allele status, *n* (%)					
Heterozygous	334 (77.5)	500 (76.9)	–	–	–
Homozygous	97 (22.5)	150 (23.1)	–	–	–
Using anti-AD medication at baseline, *n* (%)	386 (89.6)	578 (88.9)	274 (83.5)	204 (80.0)	209 (82.6)
Mean MMSE score (SD)	21.0 (3.0)	20.9 (3.1)	20.8 (3.1)	20.8 (3.2)	20.8 (3.1)
Mean years of formal education (SD)	12.5 (3.6)	12.2 (3.7)	11.8 (3.9)	11.9 (3.9)	11.8 (3.9)
Substudy participation, *n* (%)					
vMRI + PET or vMRI + PET + CSF	47 (10.9)	64 (9.8)	34 (10.4)	27 (10.6)	21 (8.3)
CSF or CSF + vMRI	104 (24.1)	160 (24.6)	67 (20.4)	44 (17.3)	53 (20.9)
vMRI only	161 (37.4)	241 (37.1)	112 (34.1)	92 (36.1)	88 (34.8)
No substudy	119 (27.6)	185 (28.5)	115 (35.1)	92 (36.1)	91 (36.0)
Mean ADAS-Cog/11 score (SD)	22.6 (8.9)	23.2 (8.9)	22.9 (10.2)	23.2 (10.0)	23.5 (9.3)
Mean DAD score (SD)	80.9 (18.7)	79.9 (18.3)	79.6 (17.9)	78.6 (20.0)	79.0 (18.4)
Infusions received, *n* (%)					
1	13 (3.0)	34 (5.2)	40 (12.2)	22 (8.6)	29 (11.5)
2	17 (3.9)	42 (6.5)	44 (13.4)	37 (14.5)	46 (18.2)
3	29 (6.7)	57 (8.8)	36 (11.0)	31 (12.2)	25 (9.9)
4	28 (6.5)	46 (7.1)	33 (10.1)	24 (9.4)	26 (10.3)
5	43 (10.0)	98 (15.1)	40 (12.2)	35 (13.7)	30 (11.9)
6	301 (69.8)	373 (57.4)	134 (40.9)^a^	106 (41.6)	97 (38.3)

*AD* Alzheimer’s disease, *ADAS-Cog/11* 11-item Alzheimer’s Disease Assessment Scale–Cognitive subscale, *ApoE* apolipoprotein E, *BAP* bapineuzumab, *CSF* cerebrospinal fluid, *DAD* Disability Assessment for Dementia, *MMSE* Mini Mental State Examination, *PET* positron emission tomography, *vMRI* volumetric magnetic resonance imaging

^a^One patient in the placebo group received seven infusions

### Demographics and baseline characteristics

In both studies, mean age at study baseline was similar across treatment groups, and the majority of patients were white and female (Table 1). Approximately 17 % were Asian, as both studies recruited patients from 36 centers in Japan. The mean duration of AD diagnosis ranged between 2.6 and 3.0 years, and ≥80 % in each group were being treated with cholinesterase inhibitors and/or memantine. The distribution of randomization stratification factors was similar across groups; among carriers, the majority of patients (approximately 77 %) had a single ApoE ε4 allele.

### Clinical efficacy

**Fig. 2:**
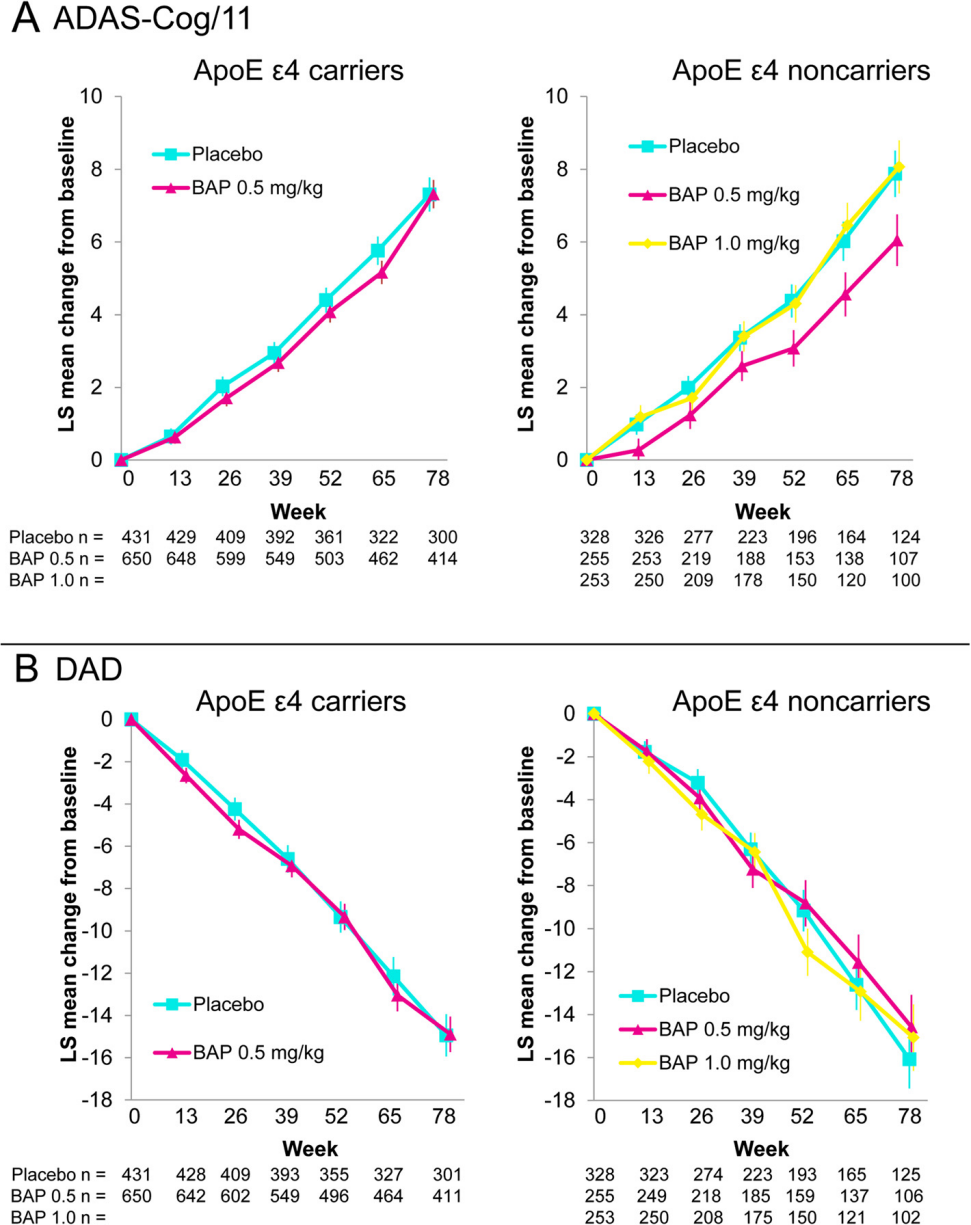
Primary efficacy outcome analysis: change from baseline to week 78. **a** ADAS-Cog/11: total score range is 0 (least impairment) to 70 (most impairment). A positive change from baseline indicates worsening cognitive impairment. **b** DAD: total score range is 0 to 100, with higher scores indicating better function. A negative change from baseline indicates worsening function. Data shown are least squares means with standard error of the mean. ADAS-Cog/11, 11-item Alzheimer’s Disease Assessment Scale–Cognitive subscale; ApoE, apolipoprotein E; BAP, bapineuzumab; DAD, Disability Assessment for Dementia; LS, least squares

In the ApoE ε4 carrier study, there was no statistically significant difference between the bapineuzumab 0.5 mg/kg and placebo groups for ADAS-Cog/11 (*p* = 0.979) or DAD (*p* = 0.973) (Fig. 2). Similarly, in noncarriers, no statistically significant difference was observed for ADAS-Cog/11 or DAD between the bapineuzumab 0.5 mg/kg and placebo groups (*p* = 0.057 and *p* = 0.459, respectively) or between the bapineuzumab 1.0 mg/kg and placebo groups (*p* = 0.848 and *p* = 0.623, respectively). An analysis of coprimary endpoints among completers yielded similar findings; no significant differences were observed for ADAS-Cog/11 or DAD in either study. For a subgroup analysis by disease severity and ApoE ε4 carrier status, see Additional file 4: Table S1.

### Additional efficacy endpoints

At week 78, there were no significant differences between bapineuzumab and placebo in the NTB total Z-score, CDR-SOB score, or Dependence Scale total score in either study, with one exception: in the noncarrier study, there was a significant difference in favor of bapineuzumab between bapineuzumab 0.5 mg/kg and placebo on NTB total Z-score (difference 0.10, *p* = 0.047). All additional efficacy endpoint data are shown in Table 2.

**Table 2:** Secondary and exploratory efficacy analyses: change from baseline to week 78

	ApoE ε4 carrier study		ApoE ε4 noncarrier study		
	PBO (*n* = 431)	BAP 0.5 (*n* = 650)	PBO (*n* = 328)	BAP 0.5 (*n* = 255)	BAP 1.0 (*n* = 253)
CDR-SOB total score^a^					
Number of subjects	310	427	144	115	110
Mean change (SD)	2.4 (2.8)	2.3 (2.9)	2.5 (2.8)	2.2 (2.8)	2.2 (2.6)
MMRM analysis					
LS mean change (SE)	2.59 (0.16)	2.44 (0.13)	2.59 (0.20)	2.23 (0.23)	2.41 (0.23)
Difference vs PBO		−0.15		−0.36	−0.18
*p* Value		0.448		0.238	0.564
DS total score^b^					
Number of subjects	316	437	145	121	112
Mean change (SD)	1.2 (2.2)	1.2 (2.3)	1.4 (2.5)	1.3 (2.0)	1.1 (2.5)
MMRM analysis					
LS mean change (SE)	1.33 (0.1)	1.22 (0.1)	1.45 (0.17)	1.29 (0.19)	1.16 (0.19)
Difference vs PBO		−0.11		−0.16	−0.29
*p* Value		0.462		0.516	0.257
NTB total Z-score^c^					
Number of subjects	296	403	120	105	96
Mean change (SD)	0.0 (0.5)	0.0 (0.6)	−0.1 (0.5)	0 (0.5)	0 (0.4)
MMRM analysis					
LS mean change (SE)	−0.11 (0.03)	−0.10 (0.02)	−0.09 (0.04)	0.02 (0.04)	−0.12 (0.04)
Difference vs PBO		0.01		0.10	−0.03
*p* Value		0.889		0.047	0.541

*ApoE* apolipoprotein E, *BAP* bapineuzumab, *CDR-SOB* Clinical Dementia Rating–Sum of Boxes, *DS* Dependence Scale, *LS* least squares, *MMRM* mixed model for repeated measures, *NTB* Neuropsychological Test Battery, *PBO* placebo

^a^CDR-SOB total score range is 0 (least impairment) to 18 (most impairment); a negative change from baseline indicates improvement

^b^DS total score range is 0–15, with higher scores indicating worse impairment; a negative change from baseline indicates improvement

^c^Positive change indicates improvement in NTB total Z-score

### Biomarkers

#### PiB-PET

**Fig. 3:**
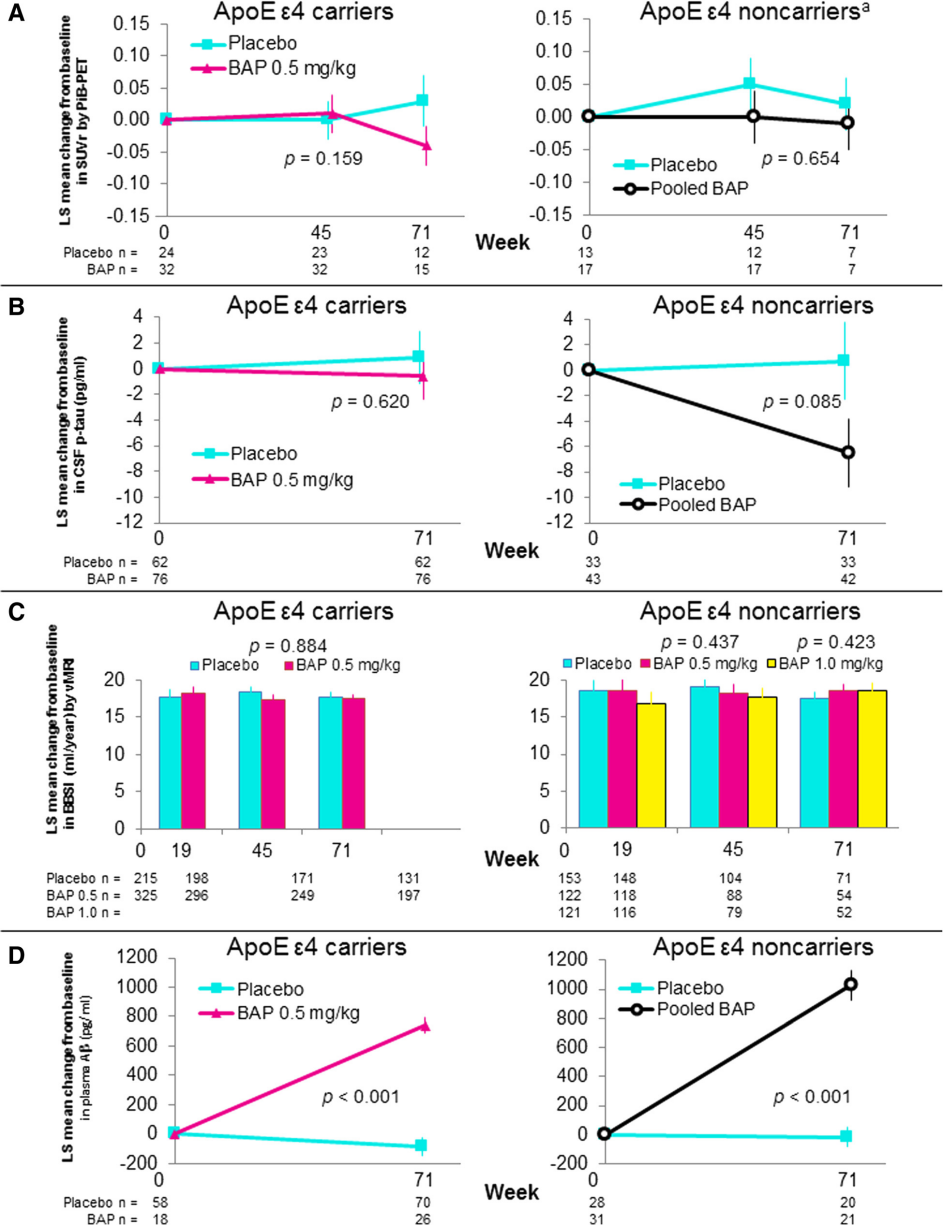
Analysis of biomarkers and plasma Aβ: change from baseline to week 71. Data shown are least squares means with standard error of the mean. **a** PiB-PET analysis (**b**) CSF p-tau analysis (**c**) Volumetric analysis: An increase in BBSI on vMRI indicates a decrease in brain volume. **d** Plasma Aβ analysis. Aβ, amyloid β; ApoE, apolipoprotein E; BAP, bapineuzumab; BBSI, brain boundary shift integral; CSF, cerebrospinal fluid; LS, least squares; PiB-PET, ^11^C-Pittsburgh compound B positron emission tomography; p-tau, phosphorylated tau protein; SUVr, standardized uptake value ratio; vMRI, volumetric magnetic resonance imaging. ^a^Excludes nine patients who were PiB-PET-negative for Aβ at baseline

**Fig. 4:**
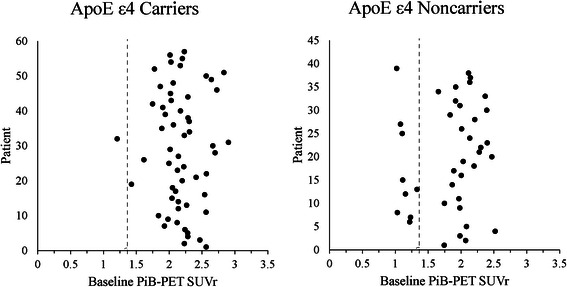
Individual patient baseline PiB-PET SUVr values (all PiB-PET population). ApoE, apolipoprotein E; PiB-PET, ^11^C-Pittsburgh compound B positron emission tomography; SUVr, standardized uptake value ratio

Change from baseline to week 71 for PiB-PET GCA SUVr was not statistically significant versus placebo in either study (Fig. 3a). Fifty-seven patients were enrolled in the ApoE ε4 carrier substudy and 39 in the noncarrier substudy. Twenty-three percent of patients did not meet the threshold of SUVr for the GCA region of interest ≥1.35 at baseline on PiB-PET in noncarriers (vs 2 % of carriers); all amyloid-negative patients were excluded from the PiB-PET population analysis. Baseline values of PiB-PET GCA SUVr (mean [standard deviation]) were similar between carriers: 2.2 (0.29) in the placebo group and 2.2 (0.31) in the bapineuzumab group, and noncarriers: 2.1 (0.21) in the placebo group and 2.1 (0.24) in the pooled bapineuzumab group. A plot of the baseline values for all patients screened for the PiB-PET substudy (Fig. 4) shows the separation between noncarriers who were classified as amyloid-negative and those who were classified as amyloid-positive. A total of 14 noncarriers and 27 carriers received the week 71 PET imaging assessment. To estimate the effect of premature study termination on statistical power, *p* values were calculated assuming that the data trend continued with full enrollment in each study. The *p* value in the carrier study was estimated to be 0.085 comparing bapineuzumab with placebo, and the *p* value was estimated to be 0.438 in the noncarrier study comparing pooled bapineuzumab with placebo.

#### CSF p-tau

The CSF carrier substudy enrolled 138 patients and the noncarrier substudy enrolled 76. Nearly all had a baseline and week 71 assessment. No significant differences were observed in CSF p-tau, although in noncarriers the difference showed a trend (*p* = 0.085) favoring the bapineuzumab pooled group (Fig. 3b).

#### vMRI

The vMRI carrier substudy enrolled 540 patients and the noncarrier substudy enrolled 401. No significant treatment difference was observed in annualized rate (milliliters per year) of whole-brain volume loss assessed by BBSI in either study (Fig. 3c). In all groups, the annual estimated decrease in whole-brain volume was approximately 18 ml/year across all treatment groups at the first follow-up assessment (week 19) as well as at subsequent assessments.

### Pharmacodynamics

Significant increases in plasma Aβ levels with bapineuzumab from baseline to week 71 were observed in both carrier (bapineuzumab-placebo difference 827.29 pg/ml, *p* < 0.001) and noncarrier (pooled bapineuzumab-placebo difference 941.86 pg/ml, *p* < 0.001) studies (Fig. 3d).

### Safety

Treatment-emergent adverse events (TEAEs) with >3 % difference in incidence between groups were ARIA-E and cerebral microhemorrhage (more with bapineuzumab) and anxiety and headache (more with placebo). For ApoE ε4 carriers, TEAEs considered treatment-related were reported in 34.6 % of patients in the bapineuzumab group and 22.1 % in the placebo group. In the ApoE ε4 noncarrier study, the incidence of TEAEs considered treatment-related was similar in the bapineuzumab 0.5 mg/kg and placebo groups (18.4 % and 18.3 % of patients, respectively), but it was higher in the bapineuzumab 1.0 mg/kg group (30.0 %). The incidences of ARIA-E for ApoE ε4 carriers were 16.7 % for the bapineuzumab 0.5 mg/kg group and 2.1 % for the placebo group. Discontinuation due to ARIA-E was higher in the bapineuzumab group (2.9 % vs 0.5 %). All prespecified events of clinical importance other than ARIA-E and hypersensitivity reactions were reported in ≤1.2 % in both treatment groups (Table 3). Seizure, deep vein thrombosis/pulmonary embolism, and intracranial hemorrhage were numerically more frequent, and hypersensitivity reaction was less frequent, in the bapineuzumab group than in the placebo group. For ApoE ε4 carriers, the incidences of intraparenchymal hemorrhage were 0.46 % and 0.76 % in the placebo and bapineuzumab groups, respectively (Table 3).

**Table 3:** Prespecified events of clinical importance—incidence proportion (95% CI)

Event, n, % (95% CI)	ApoE ε4 carrier study		ApoE ε4 noncarrier study		
	Placebo	BAP 0.5	Placebo	BAP 0.5	BAP 1.0
	(*n* = 439)	(*n* = 654)	(*n* = 344)	(*n* = 267)	(*n* = 263)
ARIA-E	9	109	2	13	31
	2.05	16.67	0.58	4.87	11.79
	(0.94, 3.86)	(13.89, 19.75)	(0.07, 2.08)	(2.62, 8.18)	(8.15, 16.3)
Intracranial hemorrhage	5	6	7	2	1
	1.14	0.92	2.03	0.75	0.38
	(0.37, 2.64)	(0.34, 1.99)	(0.82, 4.15)	(0.09, 2.68)	(0.01, 2.10)
Seizures/convulsions	1	7	3	1	0
	0.23	1.07	0.87	0.37	0.00
	(0.01, 1.26)	(0.43, 2.19)	(0.18, 2.53)	(0.01, 2.07)	(0.00, 1.39)
DVT/PE	2	8	0	1	1
	0.46	1.22	0.00	0.37	0.38
	(0.06, 1.64)	(0.53, 2.40)	(0.00, 1.07)	(0.01, 2.07)	(0.01, 2.10)
Hypersensitivity reactions	19	18	6	5	4
	4.33	2.75	1.74	1.87	1.52
	(2.63, 6.68)	(1.64, 4.32)	(0.64, 3.76)	(0.61, 4.32)	(0.42, 3.85)
Intraparenchymal hemorrhage	2	5	0	0	0
	0.46	0.76	0.00	0.00	0.00
	(0.06, 1.64)	(0.25, 1.78)	(0.00, 1.07)	(0.00, 1.37)	(0.00, 1.39)

*ApoE* apolipoprotein E, *ARIA-E* amyloid-related imaging abnormalities, edema/effusion, *BAP* bapineuzumab, *DVT/PE* deep vein thrombosis/pulmonary embolism

In noncarriers, incidences of ARIA-E were 4.9 % in the bapineuzumab 0.5 mg/kg group, 11.8 % in the bapineuzumab 1.0 mg/kg group, and 0.6 % in the placebo group (Table 3). Discontinuation due to ARIA-E was highest in the bapineuzumab 1.0 mg/kg group compared with the bapineuzumab 0.5 mg/kg and placebo groups (3.0 % vs 1.0 % and 0.6 %, respectively). All prespecified AEs other than ARIA-E occurred in ≤2.0 % in all three groups. Incidences of intracranial hemorrhage and seizure/convulsion were higher in the placebo group than in either bapineuzumab group. There was one case of deep vein thrombosis/pulmonary embolism in each of the bapineuzumab groups and no cases in the placebo group; no cases of intraparenchymal hemorrhage occurred in the noncarrier study. 

## Discussion

The presently reported studies (3000/3001) are the last of four phase 3 trials performed to evaluate bapineuzumab immunotherapy. As in the first two U.S. trials (Studies 301 and 302), no significant differences were found in the coprimary cognitive or functional endpoints in this more global demographic [3]. Early termination of the 3000/3001 studies could have contributed to the inability to detect a treatment effect; however, enrollment in the carrier (3001) study was complete (only the PET substudy was still recruiting), MMRM analysis was used, the treatment and placebo values were similar, and the negative results in the mITT and completer populations were consistent with studies 301/302 collectively, suggesting that the lack of clinical effect seen in these two studies was not due to early termination. There was no evidence of a clinical dose effect in the noncarrier study between the 0.5 mg/kg and 1.0 mg/kg doses. The small number of patients in the 2.0 mg/kg dose group (11 patients, 9 completers) and the reassignment of these patients to 1.0 mg/kg did not permit evaluation of the effects of higher doses.

The need for earlier intervention with amyloid-lowering therapy has been hypothesized as a potential reason for lack of clinical efficacy observed in recent phase 3 studies of bapineuzumab and of solanezumab, another anti-Aβ-targeted monoclonal antibody [2, 3, 8, 9]. Also, a statistically significant benefit of bapineuzumab was reported in Study 301 in a mild AD subgroup (MMSE >19) of ApoE ε4 noncarriers on the functional but not the cognitive measure [3]. On the basis of these findings, phase 3 studies in patients with mild AD and prodromal AD were initiated with solanezumab and another anti-Aβ-targeted monoclonal antibody, gantenerumab [10]. Twenty-three percent of noncarriers and two percent of carriers who had baseline PiB-PET scans met the clinical criteria for AD dementia but did not meet the PiB-PET threshold for amyloid positivity at baseline. These patients with “suspected nonamyloid pathology” may have had some other form of dementia that could not be differentiated from AD on the basis of clinical inclusion criteria. Patients with low levels of amyloid would not be expected to benefit from an antiamyloid therapy. Inclusion of subthreshold amyloid patients was also a factor in the 301/302 bapineuzumab PiB-PET substudies (36 % and 6.5 % of those with baseline scans in the noncarrier and carrier studies, respectively) [3]. The proportion of patients not meeting the amyloid threshold was lower in the 3000/3001 studies than in the 301/302 studies using the same threshold value, suggesting some differences in enrollment between the two sets of studies. The choice of amyloid threshold was based on the consensus of experts consulted at the time the studies were initiated, but the plot of baseline values shows a clear separation between positive and negative populations that would be robust to a range of selected thresholds. The amyloid-negative patients were included in the analyses of outcomes other than SUVr; however, it is unlikely that exclusion of the ten patients who were amyloid-negative would affect the overall study outcome. Since brain amyloid positivity was not an inclusion criterion, it is not possible to evaluate its impact on the observed cognitive and functional outcomes. Collectively, the findings from these studies indicate that amyloid assessment at screening is essential and that meeting an agreed amyloid threshold should be an inclusion criterion for future antiamyloid therapy trials.

The 3000/3001 bapineuzumab studies did not fully replicate the PiB-PET or CSF biomarker findings from Studies 301/302. In Studies 3000/3001, no significant treatment differences were seen in amyloid burden on PiB-PET or CSF p-tau (but trends were in the expected directions) (Fig. 3b), which appeared to be related to stability of the placebo groups over time. This finding differs from the results of Study 302 in ApoE ε4 carriers, in which SUVr on PiB-PET continued to increase in the placebo group with no change in the bapineuzumab group, suggesting prevention of Aβ accumulation. Significant decreases in amyloid load on PiB-PET with bapineuzumab were also reported from a phase 2 study [6]. However, Study 301 in noncarriers showed no change in SUVr in the placebo group and no significant difference at week 71 between placebo and the bapineuzumab 0.5 mg/kg and 1.0 mg/kg groups [3]. Mean amyloid load at baseline in amyloid-positive patients was similar between carriers and noncarriers in all four studies and between the 301/302 and 3000/3001 studies. A possible reason for lack of significance of the PiB-PET results in Studies 3000/3001 is the small number of subjects who completed each PiB-PET substudy, owing to early study termination. Only 27 patients in the current carrier study and 14 in the noncarrier study (approximately half in the bapineuzumab and half in the placebo group in each study) had an assessment at week 71, in contrast to 115 patients (75 bapineuzumab, 40 placebo) in the 302 carrier study and 39 patients (24 bapineuzumab, 15 placebo) in the 301 noncarrier study [3]. However, the projected *p* values for the PET substudies assuming full enrollment with the observed data trends suggest that full enrollment would not have changed the study conclusions. The cerebellar gray matter was used as reference region in the PET analyses, opening up another possibility that the use of the pons as reference region might have reduced the noise and thereby improved sensitivity to detect changes [11].

In Studies 3000/3001, there was no effect of treatment on CSF p-tau, although there was a trend for significant reduction in the pooled bapineuzumab group in noncarriers. This finding differs from the results of the 301/302 studies. In ApoE ε4 noncarriers in the latter studies, there was no effect on CSF p-tau concentrations in the pooled bapineuzumab groups (the prespecified analysis), but exploratory analyses showed a significant reduction in CSF p-tau with bapineuzumab 1.0 mg/kg [3]. As was the case for the PiB-PET analysis of SUVr, the results for CSF p-tau in the current studies were likely affected by the small number of patients who completed the assessments. Compared with the 301/302 studies, the number of patients in the bapineuzumab groups was less by almost half. vMRI assessments showed no significant effect of bapineuzumab treatment in any of the four studies, and rates of whole-brain volume loss were similar [3].

Plasma Aβ_*x*–40_ levels increased significantly in the bapineuzumab groups but not in the placebo group in the 3000/3001 studies, a result expected from anti-Aβ antibody infusion observed in other studies and an indicator of peripheral target engagement [2, 9].

Infusions of bapineuzumab 0.5 and 1.0 mg/kg every 13 weeks were generally well-tolerated, and the safety profile was consistent with that reported in previous studies. No new or unexpected safety findings were observed [3, 7]. ARIA-E were confirmed as dose-dependent TEAEs associated with bapineuzumab; these events increased with dose and ApoE ε4 allele number and led to discontinuation of the 2.0 mg/kg dose in all noncarrier studies [3]. Rates of ARIA-E were three to four times higher in the placebo group of ApoE ε4 carriers than among noncarriers in the current studies, which differs from what was reported in the previous studies. This difference may have been due to increased detection because of the central image-reading of every MRI scan, suggesting a need for more intensive radiologist training to detect ARIA-E, particularly in clinical trials. Further research is needed to identify risk factors for ARIA-E and their long-term clinical course [12]. There is growing evidence that ARIA is related to amyloid clearance from the brain [12], which could eventually be tested by comparing PET and MRI images from the four completed bapineuzumab studies.

### Study limitations

Early termination is the major limitation of both studies, as both were discontinued earlier than expected and before enrollment was complete in the noncarrier study. Premature termination led to smaller-than-expected sample sizes, particularly in the PiB-PET and CSF p-tau substudies. In addition, assuming that the PET substudy population is representative of the entire study population, a relatively high proportion of ApoE ε4 noncarriers did not meet a preestablished amyloid threshold and therefore lacked the drug target.

## Conclusions

Despite early termination, these studies in a more global population demographic confirm the overall negative clinical findings of the first phase 3 trials of bapineuzumab in mild to moderate AD. Some biomarker results of the 301/302 studies were not confirmed in the present studies, although small sample sizes limit their interpretation. In the future, a pooled analysis of the data from the four studies may provide more clarity on the effects of bapineuzumab on biomarkers of AD. In the PiB-PET substudy, the percentage of ApoE ε4 noncarriers with amyloid below the GCA threshold SUVr of 1.35 is lower in this study than in Study 301 (23 % vs 36 %). Assuming that the percentage is similar in the entire study population, it is still high and highlights the potential importance of meeting a preestablished amyloid threshold as an inclusion criterion when conducting future studies of this type. Preliminary studies to determine adequate blood-brain barrier penetration of therapeutic antibodies at planned study doses would also help answer questions about central target engagement. Large studies in patients with prodromal AD who have sufficient brain amyloid to confirm the diagnosis, with extended follow-up to ensure significant change from baseline in the placebo group, will likely be needed to ultimately determine the viability of an immunotherapeutic approach to AD treatment.

### Ethical approval and consent to participate

These studies were approved by the institutional review board or independent ethics committee at each site, and each patient (or the patient’s legal representative) and patient caregiver provided written informed consent before any screening procedures were performed. A complete list of all ethical bodies that approved the ApoE ε4 carrier and ApoE ε4 noncarrier studies are listed in Additional file 5 and Additional file 6, respectively.

### Availability of supporting data

Requests for access to the original data should be addressed to the study sponsors.

## Additional files

Additional file 1: Coinvestigators. Complete list of study investigators. (DOCX 46 kb) Additional file 2: Inclusion and exclusion criteria. Inclusion and exclusion criteria, similar to those used in the 301/302 studies. (DOCX 18 kb) Additional file 3: Prespecified outcomes in apolipoprotein E ε4 carrier and noncarrier trials. All prespecified endpoints are listed. (DOCX 18 kb) Additional file 4: Table S1. Change from baseline to week 78 by MMSE score at baseline (ApoE ε4 carriers and noncarriers). Table showing analysis of ADAS-Cog/11 and DAD scores by disease severity. (DOCX 14 kb) Additional file 5: List of independent ethics committees or institutional review boards. Complete list of independent ethics committees for all sites that screened subjects for the ApoE ε4 carrier study. (PDF 134 kb) Additional file 6: List of independent ethics committees or institutional review boards. Complete list of independent ethics committees for all sites that screened subjects for the ApoE ε4 noncarrier study. (PDF 339 kb)

## Abbreviations

Aβ: amyloid β
AD: Alzheimer’s disease
ADAS-Cog/11: 11-item Alzheimer’s Disease Assessment Scale–Cognitive subscale
AE: adverse event
ApoE: apolipoprotein E
ARIA-E: amyloid-related imaging abnormalities with edema or effusion
BAP: bapineuzumab
BBSI: brain boundary shift integral
CDR-SOB: Clinical Dementia Rating–Sum of Boxes
CSF: cerebrospinal fluid
DAD: Disability Assessment for Dementia
DS: Dependence Scale
DVT/PE: deep vein thrombosis/pulmonary embolism
ELISA: enzyme-linked immunosorbent assay
GCA: global cortical average
IV: intravenous
LS: least squares
mITT: modified intention to treat
MMRM: mixed model for repeated measures
MMSE: Mini Mental State Examination
MRI: magnetic resonance imaging
NTB: Neuropsychological Test Battery
PBO: placebo
PET: positron emission tomography
PiB: ^11^C-Pittsburgh compound B
p-tau: phosphorylated tau
SUVr: standardized uptake value ratio
TEAE: treatment-emergent adverse event
vMRI: volumetric magnetic resonance imaging
