# Supplementary material for: Bapineuzumab for mild to moderate Alzheimer’s disease in two global, randomized, phase 3 trials
Source: Alzheimers Res Ther. 2016 May 12;8:18. doi: 10.1186/s13195-016-0189-7 (PMC4866415; doi:10.1186/s13195-016-0189-7)
Supplement: Additional file 1: — Coinvestigators. Complete list of study investigators. (DOCX 46 kb) [file 13195_2016_189_MOESM1_ESM.docx]

**COINVESTIGATORS**

**Argentina**

Fernando Taragano, MD (CEMIC, PI); Diego Martin Castro (CEMIC, Sub-investigator); Monica Feldman (CEMIC, Sub-investigator); Maria Martelli (CEMIC, Sub-investigator); Diego Ruben Sarasola (CEMIC, Sub-investigator); Cecilia Mariela Serrano (CEMIC, Sub-investigator); Graciela Tufro (CEMIC, Sub-investigator); Oscar Boga, MD (Clínica IMECO, PI); Hector Alberto Maurino (Clínica IMECO, Sub-investigator); Silvia Alicia Pereyra (Clínica IMECO, Sub-investigator); Daniel Gustavo Politis (Clínica IMECO, Sub-investigator); Gladys Noemi Rey (Clínica IMECO, Sub-investigator); Wanda Yanina Rubinstein (Clínica IMECO, Sub-investigator); Dario Saferstein (Clínica IMECO, Sub-investigator); Gustavo Martin Stein (Clínica IMECO, Sub-investigator); Daniel Seinhart, MD (Hospital Italiano de Buenos Aires, PI); Silvina Edith Brienza (Hospital Italiano de Buenos Aires, Sub-investigator); Maria Elena Guajardo (Hospital Italiano de Buenos Aires, Sub-investigator); Luisa Edith Labos (Hospital Italiano de Buenos Aires, Sub-investigator); Bernardo Julio Martinez (Hospital Italiano de Buenos Aires, Sub-investigator); Maria Elvira Soderlund (Hospital Italiano de Buenos Aires, Sub-investigator); Sofia Andrea Trojanowski (Hospital Italiano de Buenos Aires, Sub-investigator); Ruben Carlos Vallejos (Hospital Italiano de Buenos Aires, Sub-investigator); Leonardo Kremer, MD (Instituto Kremer, PI); Maria Alejandra Amuchastegui (Instituto Kremer, Sub-investigator); Adolfo Jalowski (Instituto Kremer, Sub-investigator); Juan Francisco Luquez (Instituto Kremer, Sub-investigator); Luciana del Carmen Perret (Instituto Kremer, Sub-investigator)

**Australia**

Denis Crimmins, MD (Gosford Hospital, PI); Michelle Anderson (Gosford Hospital, Sub-investigator); Kate Cotter (Gosford Hospital, Sub-investigator); Jamie Gills (Gosford Hospital, Sub-investigator); Dayna Griffiths (Gosford Hospital, Sub-investigator); Anna Schutz (Gosford Hospital, Sub-investigator); Alison Slattery (Gosford Hospital, Sub-investigator); Jonathan Sturm (Gosford Hospital, Sub-investigator); Scott Whyte (Gosford Hospital, Sub-investigator); Veronica Zenteno (Gosford Hospital, Sub-investigator); Michael Woodward, MD (Heidelberg Repatriation Hospital, PI); Claudia Anton (Heidelberg Repatriation Hospital, Sub-investigator); Kristin Cooper (Heidelberg Repatriation Hospital, Sub-investigator); Leonie Peta Johnston (Heidelberg Repatriation Hospital, Sub-investigator); Jennifer Lesley Lowday (Heidelberg Repatriation Hospital, Sub-investigator); Silvana Micallef (Heidelberg Repatriation Hospital, Sub-investigator); Margaret Owens (Heidelberg Repatriation Hospital, Sub-investigator); Josephine Popham (Heidelberg Repatriation Hospital, Sub-investigator); Irene AR Tan (Heidelberg Repatriation Hospital, Sub-investigator); Paul Yates (Heidelberg Repatriation Hospital, Sub-investigator); Henry Zeimer (Heidelberg Repatriation Hospital, Sub-investigator); Roger Clarnette, MBBS, FRACP (The McCusker Alzheimer’s Research Foundation, PI); Fiona Cameron (The McCusker Alzheimer’s Research Foundation, Sub-investigator); Felicite Kelsall (The McCusker Alzheimer’s Research Foundation, Sub-investigator); Helen Macartney (The McCusker Alzheimer’s Research Foundation, Sub-investigator); Rocky Nyaod (The McCusker Alzheimer’s Research Foundation, Sub-investigator); Hamid Sohrabi (The McCusker Alzheimer’s Research Foundation, Sub-investigator); Jennifer Wright (The McCusker Alzheimer’s Research Foundation, Sub-investigator); Susan Kurrle, MBBS, PhD (Hornsby Ku-ring-gai Hospital, PI); Simon Se Lerg Chan (Hornsby Ku-ring-gai Hospital, Sub-investigator); Kate Fredericks (Hornsby Ku-ring-gai Hospital, Sub-investigator); Roseanne Hogarth (Hornsby Ku-ring-gai Hospital, Sub-investigator); Mohammad Enayet Karim (Hornsby Ku-ring-gai Hospital, Sub-investigator); Jane McAuliffe (Hornsby Ku-ring-gai Hospital, Sub-investigator); Cesar Uy (Hornsby Ku-ring-gai Hospital, Sub-investigator); Karyn Boundy, MBBS, FRACP (The Queen Elizabeth Hospital, PI); Shelley Casey (The Queen Elizabeth Hospital, Sub-investigator); Judith Deimel (The Queen Elizabeth Hospital, Sub-investigator); Jane Mathias (The Queen Elizabeth Hospital, Sub-investigator); Christine Pandos (The Queen Elizabeth Hospital, Sub-investigator); Cathy Short (The Queen Elizabeth Hospital, Sub-investigator); Patricia Steventon (The Queen Elizabeth Hospital, Sub-investigator); Jessica Sylvester (The Queen Elizabeth Hospital, Sub-investigator); Heather Waddy (The Queen Elizabeth Hospital, Sub-investigator); Robert Prowse, MBBS (Royal Adelaide Hospital, PI); Colin Field (Royal Adelaide Hospital, Sub-investigator); Kathy Robinson (Royal Adelaide Hospital, Sub-investigator); Alice Bourke (Royal Adelaide Hospital, Sub-investigator); Alison Marlow (Royal Adelaide Hospital, Sub-investigator); Jane Hecker (Royal Adelaide Hospital, Sub-investigator); John Maddison (Royal Adelaide Hospital, Sub-investigator); Jan Harry (Royal Adelaide Hospital, Sub-investigator); Mark Yates, MBBS, FRACP (Ballarat Health Services, PI); Toby Commerford (Ballarat Health Services, Sub-investigator); Chris Powers (Ballarat Health Services, Sub-investigator); Mark Johannesen (Ballarat Health Services, Sub-investigator); Louise Boin (Ballarat Health Services, Sub-investigator); Alison Dalziel (Ballarat Health Services, Sub-investigator); Michelle Morvell (Ballarat Health Services, Sub-investigator); Josephine Popham (Ballarat Health Services, Sub-investigator); Kerrie Shiell (Ballarat Health Services, Sub-investigator);

**Austria**

Josef Marksteiner, MD (LKH Klagenfurt, PI); Ruth Gallowitsch (LKH Klagenfurt, Sub-investigator); Johann Gasser (LKH Klagenfurt, Sub-investigator); Klaus Hausegger (LKH Klagenfurt, Sub-investigator); Erich Morak (LKH Klagenfurt, Sub-investigator); Wolfgang Pipam (LKH Klagenfurt, Sub-investigator); Barbara Sadnek (LKH Klagenfurt, Sub-investigator); Hans Schofnegger (LKH Klagenfurt, Sub-investigator); Wolfgang Springer (LKH Klagenfurt, Sub-investigator); Christian Walcher (LKH Klagenfurt, Sub-investigator); Michael Rainer, MD (Sozialmedizinisches Zentrum Ost-Donauspital, PI); Florence Eidler (Sozialmedizinisches Zentrum Ost-Donauspital, Sub-investigator); Wolfgang Krampla (Sozialmedizinisches Zentrum Ost-Donauspital, Sub-investigator); Christine Krueger-Rainer (Sozialmedizinisches Zentrum Ost-Donauspital, Sub-investigator); Stephan Wildner (Sozialmedizinisches Zentrum Ost-Donauspital, Sub-investigator); Peter Dal-Bianco, MD (Medizinische Universitaet Wien, PI); Reinhold Schmidt, MD (Medizinische Universitaet Graz, PI); Anja Grazer (Medizinische Universitaet Graz, Sub-investigator); Anita Lechner (Medizinische Universitaet Graz, Sub-investigator); Barbara Pendl (Medizinische Universitaet Graz, Sub-investigator); Katja Elisabeth Petrovic (Medizinische Universitaet Graz, Sub-investigator); Stefan Ropele (Medizinische Universitaet Graz, Sub-investigator)

**Belgium**

Miek Thys (University Hospital Gasthuisberg, Sub-investigator); Johan Van Cleemput (University Hospital Gasthuisberg, Sub-investigator); Mathieu Vandenbulcke (University Hospital Gasthuisberg, Sub-investigator); Raymond Oyen (University Hospital Gasthuisberg, Sub-investigator); Lieve Kathleen Porke (University Hospital Gasthuisberg, Sub-investigator); Hilde Vandenhout (University Hospital Gasthuisberg, Sub-investigator); Peter De Deyn, MD (ZNA Middelheim, PI); Didier De Surgeloose (ZNA Middelheim, Sub-investigators); Nore Somers (ZNA Middelheim, Sub-investigators); Willy Lemmens (ZNA Middelheim, Sub-investigators); Johan Goeman (ZNA Middelheim, Sub-investigators); Rishi Sheorajpanday (ZNA Middelheim, Sub-investigators); Jos Saerens (ZNA Middelheim, Sub-investigators); Peter Marien (ZNA Middelheim, Sub-investigators); Valerie Duquet (ZNA Middelheim, Sub-investigators); Sebastiaan Engelborghs (ZNA Middelheim, Sub-investigators); Brigitte Appel (ZNA Middelheim, Sub-investigators); Adrian Ivanoiu, MD, PhD (Cliniques Universitaires Saint-Luc, PI); Christine Baumans (Cliniques Universitaires Saint-Luc, Sub-investigator); Caroline Detry (Cliniques Universitaires Saint-Luc, Sub-investigator); Cecile Grandin (Cliniques Universitaires Saint-Luc, Sub-investigator); Anne Jeanjean (Cliniques Universitaires Saint-Luc, Sub-investigator); Pauline Meys (Cliniques Universitaires Saint-Luc, Sub-investigator); Dominique Rectem (Cliniques Universitaires Saint-Luc, Sub-investigator); Eva Turconi (Cliniques Universitaires Saint-Luc, Sub-investigator); Patrick Cras, MD (UZ Antwerpen, PI); Paul M. Parizel (UZ Antwerpen, Sub-investigator); Vicky Heyvaert (UZ Antwerpen, Sub-investigator); Gregory Helsen (UZ Antwerpen, Sub-investigator); Katlijn Schotsmans (UZ Antwerpen, Sub-investigator); Nelly Govers (UZ Antwerpen, Sub-investigator); Rizvana Amir (UZ Antwerpen, Sub-investigator); Sofie De Blauwe (UZ Antwerpen, Sub-investigator); Paul M. Parizel (UZ Antwerpen, Sub-investigator); Philip Bourgeois, MD (H.-Hartziekenhuis Roeselare – Menen, PI); Maarten Buyle (H.-Hartziekenhuis Roeselare – Menen, Sub-investigator); Frederik Clement (H.-Hartziekenhuis Roeselare – Menen, Sub-investigator); Marie-Christine Hasenbroekx (H.-Hartziekenhuis Roeselare – Menen, Sub-investigator); Jonny Naert (H.-Hartziekenhuis Roeselare – Menen, Sub-investigator); Olivier Deryck, MD (AZ Sint-Jan Brugge-Oostende, PI); Evy Maes (AZ Sint-Jan Brugge-Oostende, Sub-investigator); Joris Vlaemynck (AZ Sint-Jan Brugge-Oostende, Sub-investigator); Jan W. Casselman (AZ Sint-Jan Brugge-Oostende, Sub-investigator); Kris Van De Moortele (AZ Sint-Jan Brugge-Oostende, Sub-investigator)

**Canada**

Sandra Elizabeth Black, MD (Sunnybrook Health Science Centre, PI); Yannick Nadeau (Sunnybrook Health Science Centre, Sub-investigator); Alexandra Victorovna Kim (Sunnybrook Health Science Centre, Sub-investigator); Benjamin Lam (Sunnybrook Health Science Centre, Sub-investigator); Kie Honjo (Sunnybrook Health Science Centre, Sub-investigator); Mario Masellis (Sunnybrook Health Science Centre, Sub-investigator); Anoop Ganda (Sunnybrook Health Science Centre, Sub-investigator); Emnet Gammada (Sunnybrook Health Science Centre, Sub-investigator); Isabel Lam (Sunnybrook Health Science Centre, Sub-investigator); Robin Harry (Sunnybrook Health Science Centre, Sub-investigator); Jennifer Bray (Sunnybrook Health Science Centre, Sub-investigator); Andre Beauchesne, MD (Centre de Recherche Clinique Adapra Inc., PI); Yves Fortin (Centre de Recherche Clinique Adapra Inc., Sub-investigator); Gilles Berthelot (Centre de Recherche Clinique Adapra Inc., Sub-investigator); Isabelle Tremblay (Centre de Recherche Clinique Adapra Inc., Sub-investigator); Lyne Kelly (Centre de Recherche Clinique Adapra Inc., Sub-investigator); Sylvie Cloutier (Centre de Recherche Clinique Adapra Inc., Sub-investigator); Claude Patry, MD (ALPHA Recherche Clinique, PI); Marylene Vezina (ALPHA Recherche Clinique, Sub-investigator); Chantal Belanger (ALPHA Recherche Clinique, Sub-investigator); Jasmin Belle-Isle (ALPHA Recherche Clinique, Sub-investigator); Marjolaine Caron (ALPHA Recherche Clinique, Sub-investigator); Isabelle Tremblay (ALPHA Recherche Clinique, Sub-investigator); Jessica Maude Petit (ALPHA Recherche Clinique, Sub-investigator); Carole Hudon (ALPHA Recherche Clinique, Sub-investigator); Caroline Girard (ALPHA Recherche Clinique, Sub-investigator); Sylvie Martin (ALPHA Recherche Clinique, Sub-investigator); Marcel Germain, MD (Diex Research Inc., PI); Germain Bergeron (Diex Research Inc., Sub-investigator); Celine Boismenu (Diex Research Inc., Sub-investigator); Ginette Girard (Diex Research Inc., Sub-investigator); Nicole Pare (Diex Research Inc., Sub-investigator); Andrew Frank, MD (Bruyère Continuing Care, PI); Carole Gravelle (Bruyère Continuing Care, Sub-investigator); Stephanie Yamin (Bruyère Continuing Care, Sub-investigator); Danilo Antonio Guzman (Bruyère Continuing Care, Sub-investigator); Pierre Soucie (Bruyère Continuing Care, Sub-investigator); Rami Habib (Bruyère Continuing Care, Sub-investigator); Andrea De Hartog (Bruyère Continuing Care, Sub-investigator); Denise Latour (Bruyère Continuing Care, Sub-investigator); Giovanni C. Marotta, MD (The Centre for Memory and Aging, PI); Josephine Accarrino (The Centre for Memory and Aging, Sub-investigator); Lara Attardo (The Centre for Memory and Aging, Sub-investigator); Vincent Tsui (The Centre for Memory and Aging, Sub-investigator); David Sedran (The Centre for Memory and Aging, Sub-investigator); Peter P. Liu (The Centre for Memory and Aging, Sub-investigator); Rory H.G. Fisher (The Centre for Memory and Aging, Sub-investigator); Nalu Vit Pitcher (The Centre for Memory and Aging, Sub-investigator); Nina Dopslaff (The Centre for Memory and Aging, Sub-investigator); Alain Robillard, MD (Hôpital Maisonneuve-Rosemont, PI); Celine Chayer (Hôpital Maisonneuve-Rosemont, Sub-investigator); Louise Charrette (Hôpital Maisonneuve-Rosemont, Sub-investigator); Louise Lachapelle (Hôpital Maisonneuve-Rosemont, Sub-investigator); Marie-Lucie Trouve (Hôpital Maisonneuve-Rosemont, Sub-investigator); Nicole Lachance (Hôpital Maisonneuve-Rosemont, Sub-investigator); Pascale Demers (Hôpital Maisonneuve-Rosemont, Sub-investigator); Sharon Cohen, MD, FRCPC (Toronto Memory Program, PI); Atif Shaikh (Toronto Memory Program, Sub-investigator); Linda Schlesinger (Toronto Memory Program, Sub-investigator); Maheleth Llinas (Toronto Memory Program, Sub-investigator); Mary Reinoso (Toronto Memory Program, Sub-investigator); Sugitha Maheswaran (Toronto Memory Program, Sub-investigator); Varakini Parameswaran (Toronto Memory Program, Sub-investigator); Charles Ian Cohen (Toronto Memory Program, Sub-investigator); Ellen J. Buchman (Toronto Memory Program, Sub-investigator); Janice Y. Faulknor (Toronto Memory Program, Sub-investigator); Peter P. Liu (Toronto Memory Program, Sub-investigator); Sara Wainberg (Toronto Memory Program, Sub-investigator); Bholy Chaudhary (Toronto Memory Program, Sub-investigator); Karen Ng (Toronto Memory Program, Sub-investigator)

**Chile**

Sergio Gloger, MD (Psicomedica Research Group, PI); Ximena Cortés (Psicomedica Research Group, Sub-investigator); María Lizzette Duque (Psicomedica Research Group, Sub-investigator); Christian Caceres (Psicomedica Research Group, Sub-investigator); Juan Pablo Cleary (Psicomedica Research Group, Sub-investigator); Luz Maria Arellano (Psicomedica Research Group, Sub-investigator); Rafael Aranguiz (Psicomedica Research Group, Sub-investigator); Tania Paola Rodriguez (Psicomedica Research Group, Sub-investigator); Manuel Lavados, MD, PhD (Especialidades Medicas L&S, PI); Daniel Jimenez (Especialidades Medicas L&S, Sub-investigator); Marta Guillon (Especialidades Medicas L&S, Sub-investigator); Patricio Manuel Quezada (Especialidades Medicas L&S, Sub-investigator); Priscila Delano (Especialidades Medicas L&S, Sub-investigator); Cristina Mujica (Especialidades Medicas L&S, Sub-investigator); Gonzalo Quiroz (Especialidades Medicas L&S, Sub-investigator); Hector Zambrano (Especialidades Medicas L&S, Sub-investigator)

**Croatia**

Zlatko Trkanjec, MD, PhD (Clinical Hospital Sestre Milosrdnice, PI); Fran Borovecki (Clinical Hospital Sestre Milosrdnice, Sub-investigator); Marina Boban (Clinical Hospital Sestre Milosrdnice, Sub-investigator); Dragana Mijatovic (Clinical Hospital Sestre Milosrdnice, Sub-investigator); Josip Sekovanic (Clinical Hospital Sestre Milosrdnice, Sub-investigator); Natasa Klepac (Clinical Hospital Sestre Milosrdnice, Sub-investigator); Zdenko Mubrin, MD, PhD (University Hospital Center Zagreb, PI); Marina Boban (University Hospital Center Zagreb, Sub-investigator); Fran Borovecki (University Hospital Center Zagreb, Sub-investigator); Visnja Djakovic (University Hospital Center Zagreb, Sub-investigator); Natasa Klepac (University Hospital Center Zagreb, Sub-investigator); Ana Kocijan (University Hospital Center Zagreb, Sub-investigator); Branko Malojcic (University Hospital Center Zagreb, Sub-investigator); Dragana Mijatovic (University Hospital Center Zagreb, Sub-investigator); Josip Sekovanic (University Hospital Center Zagreb, Sub-investigator)

**Finland**

Hilkka Soininen, MD, PhD (University of Eastern Finland, PI); Anne-Mari Kantanen (University of Eastern Finland, Sub-investigator); Helena Makela (University of Eastern Finland, Sub-investigator); Ilona Hallikainen (University of Eastern Finland, Sub-investigator); Maija Pihlajamaki (University of Eastern Finland, Sub-investigator); Merja Hallikainen (University of Eastern Finland, Sub-investigator); Noora Nenonen (University of Eastern Finland, Sub-investigator); Sirkka Tanskanen (University of Eastern Finland, Sub-investigator); Teemu Paajanen (University of Eastern Finland, Sub-investigator); Veera Koponen (University of Eastern Finland, Sub-investigator); Susanna Tervo (University of Eastern Finland, Sub-investigator); Lasse Nieminen (University of Eastern Finland, Sub-investigator); Kirsti Virtanen (University of Turku, Sub-investigator); Iina Volanen (University of Turku, Sub-investigator); Laura Ponkanen (University of Turku, Sub-investigator); Mika Scheinin (University of Turku, Sub-investigator); Mikko Suomensalo (University of Turku, Sub-investigator); Minja Westerlund (University of Turku, Sub-investigator); Riitta Uimonen (University of Turku, Sub-investigator); Marita Kailajarvi (University of Turku, Sub-investigator); Kirsti Virtanen (University of Turku, Sub-investigator)

**France**

Philippe Robert, MD, PhD (Hôpital de Cimiez, PI); Renaud David (Hôpital de Cimiez, Sub-investigator); Claire Gervais (Hôpital de Cimiez, Sub-investigator); Emmanuel Mulin (Hôpital de Cimiez, Sub-investigator); Georges Niewiadomski (Hôpital de Cimiez, Sub-investigator); Marc Paccalin, MD, PhD (CHU La Milétrie, PI); Foucaud du Boisgueheneuc (CHU La Milétrie, Sub-investigator); Pierre Jean Saulnier (CHU La Milétrie, Sub-investigator); Roger Gil, MD (CHU La Milétrie, PI); Jacques Touchon, MD (CHU de Montpellier, PI); Karim Bennys (CHU de Montpellier, Sub-investigator); Audrey Gabelle (CHU de Montpellier, Sub-investigator); Lynda Touati (CHU de Montpellier, Sub-investigator); Serge Bakchine, MD (CHU de Reims, PI); Rachid Mahmoudi (CHU de Reims, Sub-investigator); Jean-luc Novella (CHU de Reims, Sub-investigator); Anelia Benarrosh (CHU de Reims, Sub-investigator); Olivier Godefroy, MD, PhD (CHU d’Amiens - Hôpital Nord, PI); Candice Picard (CHU d’Amiens - Hôpital Nord, Sub-investigator); Diane Dupuy (CHU d’Amiens - Hôpital Nord, Sub-investigator); Agnes Devendeville (CHU d’Amiens - Hôpital Nord, Sub-investigator); Pierre Jouanny (CHU d’Amiens - Hôpital Nord, Sub-investigator); Jean Marie Serot (CHU d’Amiens - Hôpital Nord, Sub-investigator); Francoise Lala (CHU de Casselardit, Sub-investigator); Pierre-Jean Ousset (CHU de Casselardit, Sub-investigator); Nathalie Sastre (CHU de Casselardit, Sub-investigator); Marie-Cecile Deneuville (CHU de Casselardit, Sub-investigator); Serge Belliard, MD, PhD (CHU Pontchaillou Rennes, PI); Olivier Michel (CHU Pontchaillou Rennes, Sub-investigator); Jean-Francois Dartigues, MD, PhD (CHU de Pellegrin, PI); Sophie Auriacombe (CHU de Pellegrin, Sub-investigator); Maritchu Doireau (CHU de Pellegrin, Sub-investigator); Alexandra Foubert-Samier (CHU de Pellegrin, Sub-investigator); Isabelle Marcet (CHU de Pellegrin, Sub-investigator); Florence Pasquier, MD (CHRU de Lille, PI); Stephanie Bombois (CHRU de Lille, Sub-investigator); Vincent Deramecourt (CHRU de Lille, Sub-investigator); Marie Anne Mackowiak (CHRU de Lille, Sub-investigator); Adeline Rollin-Sillaire (CHRU de Lille, Sub-investigator); Marion Paulin (CHRU de Lille, Sub-investigator); Anne-Sophie Rigaud, MD, PhD (Broca Hospital, PI); Catherine Bayle (Broca Hospital, Sub-investigator); Olivier Hanon (Broca Hospital, Sub-investigator); Hermine Lenoir (Broca Hospital, Sub-investigator); Marie-Laure Seux (Broca Hospital, Sub-investigator); Florence Latour (Broca Hospital, Sub-investigator); Mathieu Ceccaldi, MD, PhD (CHU de La Timone, PI); Olivier Felician (CHU de La Timone, Sub-investigator); Claude Gueriot (CHU de La Timone, Sub-investigator); Lejla Koric (CHU de La Timone, Sub-investigator); Marie Noelle Lefebvre (CHU de La Timone, Sub-investigator); Mira Didic (CHU de La Timone, Sub-investigator); Didier Hannequin, MD (CHU de Rouen, PI); Lucie Guyant Marechal (CHU de Rouen, Sub-investigator); Olivier Martinaud (CHU de Rouen, Sub-investigator); Snejana Jurici (CHU de Rouen, Sub-investigator); Frederique Dugny (CHU de Rouen, Sub-investigator); Martine Vercelletto, MD (CHU Nord Nantes, PI); Claire Boutoleau-Bretonniere (CHU Nord Nantes, Sub-investigator); Francois Sellal, MD (Hopitaux Civils de Colmar, PI); Jean-Marc Michel (Hopitaux Civils de Colmar, Sub-investigator); Vincent de la Sayette, MD (CHU Côte de Nacre, PI); Nathalie Derache (CHU Côte de Nacre, Sub-investigator); Michele Puel, MD (CHU Purpan, Sub-investigator); Fabienne Calvas (CHU Purpan, Sub-investigator); Monique Galitzki-Gerber (CHU Purpan, Sub-investigator); Jeremie Pariente (CHU Purpan, Sub-investigator); Claire Thalamas (CHU Purpan, Sub-investigator); Isabelle Roullet-Solignac, MD (Groupement Hospitalier Est, PI); Maïté Formaglio (Groupement Hospitalier Est, Sub-investigator); Alain Vighetto (Groupement Hospitalier Est, Sub-investigator); Isabelle Roullet-Solignac (Groupement Hospitalier Est, Sub-investigator); Pierre Krolak-Salmon, MD, PhD (Groupement Hospitalier Est, PI); Bernard-Francois Michel, MD (Sainte-Marguerite’s Hospital, PI); Marie Noelle Lefebvre (Sainte-Marguerite’s Hospital, Sub-investigator); Frank Rouby (Sainte-Marguerite’s Hospital, Sub-investigator); Laetitia Gueyrard (Sainte-Marguerite’s Hospital, Sub-investigator); Bruno Dubois, MD (Groupe Hospitalier Pitie-Salpetriere, PI); Claudine Kopp (Groupe Hospitalier Pitie-Salpetriere, Sub-investigator); Didier Dormont (Groupe Hospitalier Pitie-Salpetriere, Sub-investigator); Michel Kalafat (Groupe Hospitalier Pitie-Salpetriere, Sub-investigator); Sara Leder (Groupe Hospitalier Pitie-Salpetriere, Sub-investigator); Marc Teichmann (Groupe Hospitalier Pitie-Salpetriere, Sub-investigator); Marie Sarazin (Groupe Hospitalier Pitie-Salpetriere, Sub-investigator); Eve Attali (Groupe Hospitalier Pitie-Salpetriere, Sub-investigator); Olivier Rouaud, MD (CHU de Dijon, PI); Yannick Bejot (CHU de Dijon, Sub-investigator)

**Germany**

Hans Hermann Kluenemann, MD (Universitaet Regensburg am Bezirksklinikum, PI); Brigitte Schalhorn (Universitaet Regensburg am Bezirksklinikum, Sub-investigator); Maria Cristina Mendoza (Universitaet Regensburg am Bezirksklinikum, Sub-investigator); Goeran Hajak (Universitaet Regensburg am Bezirksklinikum, Sub-investigator); Vjera Holthoff, MD (Technische Universitaet Dresden, PI); Karolina Leopold (Technische Universitaet Dresden, Sub-investigator); Shirin Meyer (Technische Universitaet Dresden, Sub-investigator); Josef A. Nees (Technische Universitaet Dresden, Sub-investigator); Michael Huell, MD (Universitaetsklinikum Freiburg, PI); Bernhard Heimbach (Universitaetsklinikum Freiburg, Sub-investigator); Berit Prinz (Universitaetsklinikum Freiburg, Sub-investigator); Martin Schuhmacher (Universitaetsklinikum Freiburg, Sub-investigator); Iris Wernher (Universitaetsklinikum Freiburg, Sub-investigator); Siegfried Muhlack, MD, PhD (University Hospital Ruhr-University Bochum, PI); Milena Rockhoff (University Hospital Ruhr-University Bochum, Sub-investigator); Juergen Andrich (University Hospital Ruhr-University Bochum, Sub-investigator); Ulrike Theodoridis (University Hospital Ruhr-University Bochum, Sub-investigator); Rainer Hoffmann (University Hospital Ruhr-University Bochum, Sub-investigator); Joanna Schoellhammer (University Hospital Ruhr-University Bochum, Sub-investigator); Christine von Arnim, MD (Universitaetsklinikum Ulm, PI); Sonja Fuchs (Universitaetsklinikum Ulm, Sub-investigator); Jan Kassubek (Universitaetsklinikum Ulm, Sub-investigator); Madlen Pflueger (Universitaetsklinikum Ulm, Sub-investigator); Therese Poehler (Universitaetsklinikum Ulm, Sub-investigator); Doerte Polivka (Universitaetsklinikum Ulm, Sub-investigator); Oliver Peters, MD (Universitatsmedizin Berlin, PI); Britta Jaenen (Universitatsmedizin Berlin, Sub-investigator); Alexander Luborzewski (Universitatsmedizin Berlin, Sub-investigator); Klaus Schmidtke, MD (Ortenau Klinikum, PI); Eva Haefner (Ortenau Klinikum, Sub-investigator); Iris Wernher (Ortenau Klinikum, Sub-investigator)

**Italy**

Carlo Caltagirone, MD (Fondazione Santa Lucia, PI); Luca Cravello (Fondazione Santa Lucia, Sub-investigator); Fulvia Di Iulio (Fondazione Santa Lucia, Sub-investigator); Simona Gabriella Di Santo (Fondazione Santa Lucia, Sub-investigator); Giacomo Luccichenti (Fondazione Santa Lucia, Sub-investigator); Federica Lupo (Fondazione Santa Lucia, Sub-investigator); Ilenia Debora Mazzu (Fondazione Santa Lucia, Sub-investigator); Umberto Sabatini (Fondazione Santa Lucia, Sub-investigator); Silvia Zabberoni (Fondazione Santa Lucia, Sub-investigator); Stefano Francesco Cappa, MD (Ospedale San Raffaele IRCCS, PI); Andrea Falini (Ospedale San Raffaele IRCCS, Sub-investigator); Sandro Iannaccone (Ospedale San Raffaele IRCCS, Sub-investigator); Alessandra Marcone (Ospedale San Raffaele IRCCS, Sub-investigator); Maria Cristina Giusti (Ospedale San Raffaele IRCCS, Sub-investigator); Michele Zamboni (Ospedale San Raffaele IRCCS, Sub-investigator); Sara Marelli (Ospedale San Raffaele IRCCS, Sub-investigator); Valeria Golzi (Ospedale San Raffaele IRCCS, Sub-investigator); Fabrizio Tagliavini, MD (Fondazione IRCCS-Instituto Neurologico Carlo Besto, PI); Alessandra Erbetta (Fondazione IRCCS-Instituto Neurologico Carlo Besto, Sub-investigator); Dominga Paridi (Fondazione IRCCS-Instituto Neurologico Carlo Besto, Sub-investigator); Eleonora Orena (Fondazione IRCCS-Instituto Neurologico Carlo Besto, Sub-investigator); Giuseppe Di Fede (Fondazione IRCCS-Instituto Neurologico Carlo Besto, Sub-investigator); Lorenzo Nanetti (Fondazione IRCCS-Instituto Neurologico Carlo Besto, Sub-investigator); Sara Prioni (Fondazione IRCCS-Instituto Neurologico Carlo Besto, Sub-investigator); Valentina Pilato (Fondazione IRCCS-Instituto Neurologico Carlo Besto, Sub-investigator); Veronica Redaelli (Fondazione IRCCS-Instituto Neurologico Carlo Besto, Sub-investigator); Leandro Provinciali, MD (Università Politecnica delle Marche, PI); Gabriele Polonara (Università Politecnica delle Marche, Sub-investigator); Giovanni Flamma (Università Politecnica delle Marche, Sub-investigator); Sara Baldinelli (Università Politecnica delle Marche, Sub-investigator); Valentina Cameriere (Università Politecnica delle Marche, Sub-investigator); Giovanna Viticchi (Università Politecnica delle Marche, Sub-investigator); Katia Fabi (Università Politecnica delle Marche, Sub-investigator); Simona Luzzi (Università Politecnica delle Marche, Sub-investigator); Viviana Cafazzo (Università Politecnica delle Marche, Sub-investigator); Sandro Sorbi, MD (Azienda Ospedaliero Universitaria Careggi, PI); Camilla Ferrari (Azienda Ospedaliero Universitaria Careggi, Sub-investigator); Guia Martinenghi (Azienda Ospedaliero Universitaria Careggi, Sub-investigator); Laura Bracco (Azienda Ospedaliero Universitaria Careggi, Sub-investigator); Linda Beccani (Azienda Ospedaliero Universitaria Careggi, Sub-investigator); Sandro Marini (Azienda Ospedaliero Universitaria Careggi, Sub-investigator); Sonia Padiglioni (Azienda Ospedaliero Universitaria Careggi, Sub-investigator); Valentina Bessi (Azienda Ospedaliero Universitaria Careggi, Sub-investigator); Antonio Federico, MD (Università degli Studi di Siena, PI); Alessandra Carluccio (Università degli Studi di Siena, Sub-investigator); Alfonso Cerase (Università degli Studi di Siena, Sub-investigator); Francesca Forte (Università degli Studi di Siena, Sub-investigator); Elena Pretegiani (Università degli Studi di Siena, Sub-investigator); Alessandra Rufa (Università degli Studi di Siena, Sub-investigator); Maria Laura Stromillo (Università degli Studi di Siena, Sub-investigator); Enza Zicari (Università degli Studi di Siena, Sub-investigator); Maria Dotti (Università degli Studi di Siena, Sub-investigator); Marco Onofrj, MD (Università “G. D’Annunzio,” PI); Francesca Anzellotti (Università “G. D’Annunzio,” Sub-investigator); Stefania Bifolchetti (Università “G. D’Annunzio,” Sub-investigator); Laura Bonanni (Università “G. D’Annunzio,” Sub-investigator); Fausta Ciccocioppo (Università “G. D’Annunzio,” Sub-investigator); Anna D'Andrea Giovanni (Università “G. D’Annunzio,” Sub-investigator); Daniela Monaco (Università “G. D’Annunzio,” Sub-investigator); Armando Tartaro (Università “G. D’Annunzio,” Sub-investigator); Astrid Thomas (Università “G. D’Annunzio,” Sub-investigator); Raffaella Carnoso (Università “G. D’Annunzio,” Sub-investigator); Alessandro Padovani, MD, PhD (Azienda Ospedaliera “Spedali Civili,” PI); Antonella Alberici (Azienda Ospedaliera “Spedali Civili,” Sub-investigator); Elisabetta Cottini (Azienda Ospedaliera “Spedali Civili,” Sub-investigator); Enrico Premi (Azienda Ospedaliera “Spedali Civili,” Sub-investigator); Erminio Costanzo, MD (Azienda Ospedaliera per l’Emergenza Ospedale Cannizzaro, PI); Giuseppe Castro (Azienda Ospedaliera per l’Emergenza Ospedale Cannizzaro, Sub-investigator); Noemi Russo (Azienda Ospedaliera per l’Emergenza Ospedale Cannizzaro, Sub-investigator); Delfo Matarazzo (Azienda Ospedaliera per l’Emergenza Ospedale Cannizzaro, Sub-investigator); Giuseppe Caravaglios (Azienda Ospedaliera per l’Emergenza Ospedale Cannizzaro, Sub-investigator); Roberto Marziolo (Azienda Ospedaliera per l’Emergenza Ospedale Cannizzaro, Sub-investigator); Francesco Luigi Le Pira, MD (Azienda Ospedaliero-Universitaria Policlinico – Vittorio Emanuele di Catania, PI); M. Luisa Capuana (Azienda Ospedaliero-Universitaria Policlinico – Vittorio Emanuele di Catania, Sub-investigator); Tiziana Maci (Azienda Ospedaliero-Universitaria Policlinico – Vittorio Emanuele di Catania, Sub-investigator); Daniela Modica (Azienda Ospedaliero-Universitaria Policlinico – Vittorio Emanuele di Catania, Sub-investigator); Giuseppe Pero (Azienda Ospedaliero-Universitaria Policlinico – Vittorio Emanuele di Catania, Sub-investigator); Carlo Ferrarese, MD (Università di Milano-Bicocca, PI); Francesca Ferri (Università di Milano-Bicocca, Sub-investigator); Marco Grimaldi (Università di Milano-Bicocca, Sub-investigator); Cristina Mapelli (Università di Milano-Bicocca, Sub-investigator); Giorgio Gelosa (Università di Milano-Bicocca, Sub-investigator); Gisella Costantino (Università di Milano-Bicocca, Sub-investigator); Ildebrando Appollonio (Università di Milano-Bicocca, Sub-investigator); Lucio Tremolizzo (Università di Milano-Bicocca, Sub-investigator); Valeria Isella (Università di Milano-Bicocca, Sub-investigator); Paolo Rossini, MD (Università Campus Bio-Medico di Roma, PI); Federica Bressi (Università Campus Bio-Medico di Roma, Sub-investigator); Paola Chiovenda (Università Campus Bio-Medico di Roma, Sub-investigator); Laura Parisi (Università Campus Bio-Medico di Roma, Sub-investigator); Carlo Cosimo Quattrocchi (Università Campus Bio-Medico di Roma, Sub-investigator); Francesca Ursini (Università Campus Bio-Medico di Roma, Sub-investigator)

**Japan**

Ryo Murakawa, MD (National Hospital Organization Kokura Medical Center, PI); Hiroshi Shiozaki (National Hospital Organization Kokura Medical Center, Sub-investigator); Koji Sasaki Satoshi Yamada (National Hospital Organization Kokura Medical Center, Sub-investigator); Shuichi Isomura (National Hospital Organization Kokura Medical Center, Sub-investigator); Soichiro Wasano (National Hospital Organization Kokura Medical Center, Sub-investigator); Taro Setoguchi (National Hospital Organization Kokura Medical Center, Sub-investigator); Tatsuyuki Ishibashi (National Hospital Organization Kokura Medical Center, Sub-investigator); Yusuke Yamauchi (National Hospital Organization Kokura Medical Center, Sub-investigator); Hidekazu Ishiuchi (National Hospital Organization Kokura Medical Center, Sub-investigator); Hiroyuki Shimizu (National Hospital Organization Kokura Medical Center, Sub-investigator); Masamichi Shigyo (National Hospital Organization Kokura Medical Center, Sub-investigator); Shinichiro Ohsawa (National Hospital Organization Kokura Medical Center, Sub-investigator); Yuji Takeuchi (National Hospital Organization Kokura Medical Center, Sub-investigator); Hironobu Abe (National Hospital Organization Kokura Medical Center, Sub-investigator); Mieh Kim (National Hospital Organization Kokura Medical Center, Sub-investigator); Mihoko Kamada (National Hospital Organization Kokura Medical Center, Sub-investigator); Ryota Sakamoto (National Hospital Organization Kokura Medical Center, Sub-investigator); Yasuhiro Nakajima (National Hospital Organization Kokura Medical Center, Sub-investigator); Kanako Wajima (National Hospital Organization Kokura Medical Center, Sub-investigator); Masako Ishizaka (National Hospital Organization Kokura Medical Center, Sub-investigator); Wasano Soichiro (National Hospital Organization Kokura Medical Center, Sub-investigator); Toshinari Odawara, MD (Yokohama City University Medical Center, PI); Shogo Komatsu Akiko Kobayashi (Yokohama City University Medical Center, Sub-investigator); Munetaka Nomoto (Yokohama City University Medical Center, Sub-investigator); Toyo Suzuki (Yokohama City University Medical Center, Sub-investigator); Daizo Kondo (Yokohama City University Medical Center, Sub-investigator); Toru Amagai (Yokohama City University Medical Center, Sub-investigator); Atsuhiko Watanabe (Yokohama City University Medical Center, Sub-investigator); Mina Jinno (Yokohama City University Medical Center, Sub-investigator); Takashi Haraguchi, MD (National Hospital Organization Minami-Okayama Medical Center, PI); Keiko Suenaga (National Hospital Organization Minami-Okayama Medical Center, Sub-investigator); Koki Terachi (National Hospital Organization Minami-Okayama Medical Center, Sub-investigator); Taiji Nagai (National Hospital Organization Minami-Okayama Medical Center, Sub-investigator); Yumiko Kozuka (National Hospital Organization Minami-Okayama Medical Center, Sub-investigator); Hidenori Yoshida (National Hospital Organization Minami-Okayama Medical Center, Sub-investigator); Keigo Nobukuni (National Hospital Organization Minami-Okayama Medical Center, Sub-investigator); Kenichi Sakai (National Hospital Organization Minami-Okayama Medical Center, Sub-investigator); Naoko Katayama (National Hospital Organization Minami-Okayama Medical Center, Sub-investigator); Shigeto Nagao (National Hospital Organization Minami-Okayama Medical Center, Sub-investigator); Yasuyuki Tanabe (National Hospital Organization Minami-Okayama Medical Center, Sub-investigator); Yoshito Tanaka (National Hospital Organization Minami-Okayama Medical Center, Sub-investigator); Yuuetsu Ihara (National Hospital Organization Minami-Okayama Medical Center, Sub-investigator); Aki Moriki (National Hospital Organization Minami-Okayama Medical Center, Sub-investigator); Kimie Ariyoshi (National Hospital Organization Minami-Okayama Medical Center, Sub-investigator); Mika Kawasaki (National Hospital Organization Minami-Okayama Medical Center, Sub-investigator); Nobuto Shibata, MD (Juntendo University Hospital, PI); Aiko Kodaka (Juntendo University Hospital, Sub-investigator); Ayaka Miyata (Juntendo University Hospital, Sub-investigator); Emi Satomura (Juntendo University Hospital, Sub-investigator); Heii Arai (Juntendo University Hospital, Sub-investigator); Hiromi Shimazaki (Juntendo University Hospital, Sub-investigator); Masanobu Ito (Juntendo University Hospital, Sub-investigator); Miwa Komatsu (Juntendo University Hospital, Sub-investigator); Tokiko Hatano (Juntendo University Hospital, Sub-investigator); Tsuneyoshi Ota (Juntendo University Hospital, Sub-investigator); Yuko Tagata (Juntendo University Hospital, Sub-investigator); Satoshi Nakajima, PhD (National Hospital Organization Niigata National Hospital, PI); Izumi Aida (National Hospital Organization Niigata National Hospital, Sub-investigator); Shinya Higuchi (National Hospital Organization Niigata National Hospital, Sub-investigator); Suguru Takahashi (National Hospital Organization Niigata National Hospital, Sub-investigator); Yosuke Yonemochi (National Hospital Organization Niigata National Hospital, Sub-investigator); Masatoshi Fuse (National Hospital Organization Niigata National Hospital, Sub-investigator); Naoyuki Shibayama (National Hospital Organization Niigata National Hospital, Sub-investigator); Ayaka Tokuma (National Hospital Organization Niigata National Hospital, Sub-investigator); Yasumasa Yoshiyama, MD, PhD (National Hospital Organization Chiba-East Hospital, PI); Ayumi Noju (National Hospital Organization Chiba-East Hospital, Sub-investigator); Sagiri Isose (National Hospital Organization Chiba-East Hospital, Sub-investigator); Kimihito Arai (National Hospital Organization Chiba-East Hospital, Sub-investigator); Kimiko Ito (National Hospital Organization Chiba-East Hospital, Sub-investigator); Mami Kato (National Hospital Organization Chiba-East Hospital, Sub-investigator); Satoko Otokozawa (National Hospital Organization Chiba-East Hospital, Sub-investigator); Yuki Ogata (National Hospital Organization Chiba-East Hospital, Sub-investigator); Yu Nakamura, MD, PhD (Kagawa University Hospital, PI); Yuka Kashimoto (Kagawa University Hospital, Sub-investigator); Ai Usui (Kagawa University Hospital, Sub-investigator); Hideki Imai (Kagawa University Hospital, Sub-investigator); Hideto Shinno (Kagawa University Hospital, Sub-investigator); Hiromi Kuma (Kagawa University Hospital, Sub-investigator); Ichiro Ishikawa (Kagawa University Hospital, Sub-investigator); Mami Yamanaka (Kagawa University Hospital, Sub-investigator); Mizue Kido (Kagawa University Hospital, Sub-investigator); Nobuo Ando (Kagawa University Hospital, Sub-investigator); Sayaka Ishihara (Kagawa University Hospital, Sub-investigator); Shigeaki Sakamoto (Kagawa University Hospital, Sub-investigator); Shuhei Fukumoto (Kagawa University Hospital, Sub-investigator); Sonoko Danjyo (Kagawa University Hospital, Sub-investigator); Tadashi Yokoi (Kagawa University Hospital, Sub-investigator); Miyuki Ueno (Kagawa University Hospital, Sub-investigator); Tomomi Shinohara (Kagawa University Hospital, Sub-investigator); Tomokazu Obi, MD (Shizuoka Institute of Epilepsy and Neurological Disorders, PI); Kouichi Mizoguchi (Shizuoka Institute of Epilepsy and Neurological Disorders, Sub-investigator); Akira Sugiura (Shizuoka Institute of Epilepsy and Neurological Disorders, Sub-investigator); Kinya Yamazaki (Shizuoka Institute of Epilepsy and Neurological Disorders, Sub-investigator); Nobuyasu Yagi (Shizuoka Institute of Epilepsy and Neurological Disorders, Sub-investigator); Yoshinobu Kasai (Shizuoka Institute of Epilepsy and Neurological Disorders, Sub-investigator); Fumino Kato (Shizuoka Institute of Epilepsy and Neurological Disorders, Sub-investigator); Takami Miki, MD (Osaka City University Hospital, PI); Ayumi Yokote (Osaka City University Hospital, Sub-investigator); Masaaki Yasui (Osaka City University Hospital, Sub-investigator); Yuki Iwamoto (Osaka City University Hospital, Sub-investigator); Akiko Tamura (Osaka City University Hospital, Sub-investigator); Akitoshi Takeda (Osaka City University Hospital, Sub-investigator); Hideki Masaki (Osaka City University Hospital, Sub-investigator); Hiroshi Naka (Osaka City University Hospital, Sub-investigator); Hiroyuki Shimada (Osaka City University Hospital, Sub-investigator); Kazuhiro Ito (Osaka City University Hospital, Sub-investigator); Suzuka Ataka (Osaka City University Hospital, Sub-investigator); Jun Takeuchi (Osaka City University Hospital, Sub-investigator); Motokatsu Kanemoto (Osaka City University Hospital, Sub-investigator); Rie Azuma (Osaka City University Hospital, Sub-investigator); Yukie Fukumoto (Osaka City University Hospital, Sub-investigator); Eizo Iseki, MD (Juntendo Tokyo Koto Geriatric Medical Center, PI); Hideki Sugiyama (Juntendo Tokyo Koto Geriatric Medical Center, Sub-investigator); Hiroshige Fujishiro (Juntendo Tokyo Koto Geriatric Medical Center, Sub-investigator); Kanae Hayashi (Juntendo Tokyo Koto Geriatric Medical Center, Sub-investigator); Maiko Kitazawa (Juntendo Tokyo Koto Geriatric Medical Center, Sub-investigator); Mayumi Yamagata (Juntendo Tokyo Koto Geriatric Medical Center, Sub-investigator); Motohiro Nozawa (Juntendo Tokyo Koto Geriatric Medical Center, Sub-investigator); Natsuki Kamagata (Juntendo Tokyo Koto Geriatric Medical Center, Sub-investigator); Ryo Kumagai (Juntendo Tokyo Koto Geriatric Medical Center, Sub-investigator); Yoichiro Matsubara (Juntendo Tokyo Koto Geriatric Medical Center, Sub-investigator); Sayoko Ito (Juntendo Tokyo Koto Geriatric Medical Center, Sub-investigator); Noriko Matsumoto (Juntendo Tokyo Koto Geriatric Medical Center, Sub-investigator); Yohei Kita (Juntendo Tokyo Koto Geriatric Medical Center, Sub-investigator); Yoshiaki Nishida (Juntendo Tokyo Koto Geriatric Medical Center, Sub-investigator); Yosuke Ichimiya (Juntendo Tokyo Koto Geriatric Medical Center, Sub-investigator); Yosuke Ichimiya (Juntendo Tokyo Koto Geriatric Medical Center, Sub-investigator); Yuta Manabe (Juntendo Tokyo Koto Geriatric Medical Center, Sub-investigator); Kazumi Ota (Juntendo Tokyo Koto Geriatric Medical Center, Sub-investigator); Yuki Ebihara (Juntendo Tokyo Koto Geriatric Medical Center, Sub-investigator); Yosuke Ichimiya, MD (Juntendo Tokyo Koto Geriatric Medical Center, PI); Haruo Hanyu, MD (Tokyo Medical University Hospital, PI); Ayaka Miyata (Tokyo Medical University Hospital, Sub-investigator); Hidekazu Kanetaka (Tokyo Medical University Hospital, Sub-investigator); Hirofumi Sakurai (Tokyo Medical University Hospital, Sub-investigator); Kentaro Hirao (Tokyo Medical University Hospital, Sub-investigator); Takahiko Umahara (Tokyo Medical University Hospital, Sub-investigator); Soichiro Shimizu (Tokyo Medical University Hospital, Sub-investigator); Shunichi Koyama (Tokyo Medical University Hospital, Sub-investigator); Kaori Arashino (Tokyo Medical University Hospital, Sub-investigator); Kaori Funakoshi (Tokyo Medical University Hospital, Sub-investigator); Mikako Murakami (Tokyo Medical University Hospital, Sub-investigator); Shigenobu Nakamura, MD, PhD (Rakuwakai Otowa Hospital, PI); Ikuo Tooyama (Rakuwakai Otowa Hospital, Sub-investigator); Kyoko Ozawa (Rakuwakai Otowa Hospital, Sub-investigator); Masahiro Tomoi (Rakuwakai Otowa Hospital, Sub-investigator); Tomoharu Kinoshita (Rakuwakai Otowa Hospital, Sub-investigator); Kiyohide Usami (Rakuwakai Otowa Hospital, Sub-investigator); Koichi Ando (Rakuwakai Otowa Hospital, Sub-investigator); Shuichi Hirose (Rakuwakai Otowa Hospital, Sub-investigator); Tadashi Ino (Rakuwakai Otowa Hospital, Sub-investigator); Toru Kimura (Rakuwakai Otowa Hospital, Sub-investigator); Ai Hamaguchi (Rakuwakai Otowa Hospital, Sub-investigator); Takako Aida (Rakuwakai Otowa Hospital, Sub-investigator); Tamiko Hayashi (Rakuwakai Otowa Hospital, Sub-investigator); Koji Abe, MD, PhD (Okayama University Hospital, PI); Misaki Hisatomi (Okayama University Hospital, Sub-investigator); Katsuya Kato (Okayama University Hospital, Sub-investigator); Tomoko Kurata (Okayama University Hospital, Sub-investigator); Kentaro Deguchi (Okayama University Hospital, Sub-investigator); Masami Ikeda (Okayama University Hospital, Sub-investigator); Nobutoshi Morimoto (Okayama University Hospital, Sub-investigator); Shoko Nagotani (Okayama University Hospital, Sub-investigator); Tohru Matsuura (Okayama University Hospital, Sub-investigator); Yasuyuki Ohta (Okayama University Hospital, Sub-investigator); Yoshio Ikeda (Okayama University Hospital, Sub-investigator); Keiko Matsuo (Okayama University Hospital, Sub-investigator); Keiko Muneno (Okayama University Hospital, Sub-investigator); Makiko Horiuchi (Okayama University Hospital, Sub-investigator); Kazuo Shigematsu, MD (National Hospital Organization Minami-Kyoto Hospital, PI); Hiroshi Hasegawa (National Hospital Organization Minami-Kyoto Hospital, Sub-investigator); Takahiro Ito (National Hospital Organization Minami-Kyoto Hospital, Sub-investigator); Miyuki Iwasa (National Hospital Organization Minami-Kyoto Hospital, Sub-investigator); Chiiko Kashiwagi (National Hospital Organization Minami-Kyoto Hospital, Sub-investigator); Nobuyuki Oka (National Hospital Organization Minami-Kyoto Hospital, Sub-investigator); Hiroshi Sugiyama (National Hospital Organization Minami-Kyoto Hospital, Sub-investigator); Chigusa Watanabe, MD (National Hospital Organization Hiroshima-Nishi Medical Center, PI); Itsuro Ohta (National Hospital Organization Hiroshima-Nishi Medical Center, Sub-investigator); Masahiro Higaki (National Hospital Organization Hiroshima-Nishi Medical Center, Sub-investigator); Syoso Munemasa (National Hospital Organization Hiroshima-Nishi Medical Center, Sub-investigator); Takahiro Suzuki (National Hospital Organization Hiroshima-Nishi Medical Center, Sub-investigator); Takako Makino (National Hospital Organization Hiroshima-Nishi Medical Center, Sub-investigator); Yoshiro Tachiyama (National Hospital Organization Hiroshima-Nishi Medical Center, Sub-investigator); Kenji Miyasaka (National Hospital Organization Hiroshima-Nishi Medical Center, Sub-investigator); Aki Moriki (National Hospital Organization Hiroshima-Nishi Medical Center, Sub-investigator); Kaori Minami (National Hospital Organization Hiroshima-Nishi Medical Center, Sub-investigator); Miyo Hanamoto (National Hospital Organization Hiroshima-Nishi Medical Center, Sub-investigator); Sawako Arai (National Hospital Organization Hiroshima-Nishi Medical Center, Sub-investigator); Sumiyo Kawase (National Hospital Organization Hiroshima-Nishi Medical Center, Sub-investigator); Hiroshi Kurisaki, MD (National Hospital Organization Tokyo National Hospital, PI); Mariko Ootsuka (National Hospital Organization Tokyo National Hospital, Sub-investigator); Mitsuko Horibe (National Hospital Organization Tokyo National Hospital, Sub-investigator); Harumi Shiroyama (National Hospital Organization Tokyo National Hospital, Sub-investigator); Nobutaka Ishizu (National Hospital Organization Tokyo National Hospital, Sub-investigator); Satoko Kamiya (National Hospital Organization Tokyo National Hospital, Sub-investigator); Satomi Inoue (National Hospital Organization Tokyo National Hospital, Sub-investigator); Yuko Uchida (National Hospital Organization Tokyo National Hospital, Sub-investigator); Akira Yoshioka, MD (National Hospital Organization Maizuru Medical Center, PI); Rei Yasuda (National Hospital Organization Maizuru Medical Center, Sub-investigator); Takuma Omichi (National Hospital Organization Maizuru Medical Center, Sub-investigator); Yuko Hayashi (National Hospital Organization Maizuru Medical Center, Sub-investigator); Yumiko Azuma (National Hospital Organization Maizuru Medical Center, Sub-investigator); Natsuko Yuki (National Hospital Organization Maizuru Medical Center, Sub-investigator); Reiko Mizutani (National Hospital Organization Maizuru Medical Center, Sub-investigator); Hiroshi Nishikawa (National Hospital Organization Maizuru Medical Center, Sub-investigator); Kazuaki Nishimura (National Hospital Organization Maizuru Medical Center, Sub-investigator); Masafumi Yoshimura, MD, PhD (Kansai Medical University Takii Hospital, PI); Ayano Hirota PhD (Kansai Medical University Takii Hospital, Sub-investigator); Azusa Suwa (Kansai Medical University Takii Hospital, Sub-investigator); Tatsuya Nishida (Kansai Medical University Takii Hospital, Sub-investigator); Toshihiko Kinoshita (Kansai Medical University Takii Hospital, Sub-investigator); Aran Tajika (Kansai Medical University Takii Hospital, Sub-investigator); Keiichiro Nishida (Kansai Medical University Takii Hospital, Sub-investigator); Tatsuya Sugimoto (Kansai Medical University Takii Hospital, Sub-investigator); Yoshiteru Takekita (Kansai Medical University Takii Hospital, Sub-investigator); Koichi Okamoto, MD (Gunma University Hospital, PI); Yuichi Tashiro (Gunma University Hospital, Sub-investigator); Yuji Mizuno (Gunma University Hospital, Sub-investigator); Masaki Ikeda (Gunma University Hospital, Sub-investigator); Tsuneo Yamazaki (Gunma University Hospital, Sub-investigator); Yasuji Yamamoto, MD, PhD (Kobe University Hospital, PI); Masako Kuranaga (Kobe University Hospital, Sub-investigator); Kazuo Sakai (Kobe University Hospital, Sub-investigator); Masahiko Fujii (Kobe University Hospital, Sub-investigator); Noriko Hasegawa (Kobe University Hospital, Sub-investigator); Toshio Kawamata (Kobe University Hospital, Sub-investigator); Yasuo Yoshihara (Kobe University Hospital, Sub-investigator); Mariko Shiroshima (Kobe University Hospital, Sub-investigator); Mizuho Adachi (Kobe University Hospital, Sub-investigator); Naoko Iwamoto (Kobe University Hospital, Sub-investigator); Sachi Takegawa (Kobe University Hospital, Sub-investigator); Noriyuki Matsukawa, MD (Nagoya City University Hospital, PI); Yasuko Hoshikawa (Nagoya City University Hospital, Sub-investigator); Fumiyasu Ishii (Nagoya City University Hospital, Sub-investigator); Tetsuko Kanamori (Nagoya City University Hospital, Sub-investigator); Shoji Kawashima (Nagoya City University Hospital, Sub-investigator); Toshiyasu Miura (Nagoya City University Hospital, Sub-investigator); Takuya Oguri (Nagoya City University Hospital, Sub-investigator); Kenji Okita (Nagoya City University Hospital, Sub-investigator); Takafumi Sagisaka (Nagoya City University Hospital, Sub-investigator); Keita Sakurai (Nagoya City University Hospital, Sub-investigator); Yoko Shimizu (Nagoya City University Hospital, Sub-investigator); Miwako Shimizu (Nagoya City University Hospital, Sub-investigator); Takanari Toyoda (Nagoya City University Hospital, Sub-investigator); Norihiko Ucmatsu (Nagoya City University Hospital, Sub-investigator); Yoshino Ueki (Nagoya City University Hospital, Sub-investigator); Takehiko Yamanaka (Nagoya City University Hospital, Sub-investigator); Manabu Hattori (Nagoya City University Hospital, Sub-investigator); Masatoshi Takeda, MD, PhD (Osaka University Graduate School of Medicine, PI); Toshihisa Tanaka (Osaka University Graduate School of Medicine, Sub-investigator); Yutaka Inoue (Osaka University Graduate School of Medicine, Sub-investigator); Kenji Yoshiyama (Osaka University Graduate School of Medicine, Sub-investigator); Hiroaki Kazui (Osaka University Graduate School of Medicine, Sub-investigator); Shinji Tagami (Osaka University Graduate School of Medicine, Sub-investigator); Takashi Morihara (Osaka University Graduate School of Medicine, Sub-investigator); Mami Watanabe (Osaka University Graduate School of Medicine, Sub-investigator); Naoko Takeuchi (Osaka University Graduate School of Medicine, Sub-investigator); Yuko Kawaguchi (Osaka University Graduate School of Medicine, Sub-investigator); Hiromasa Tokunaga (Osaka University Graduate School of Medicine, Sub-investigator); Kiyoshi Kanaya, MD (Tokyo Medical University Hachioji Medical Center, PI); Shine Abe (Tokyo Medical University Hachioji Medical Center, Sub-investigator); Hiroko Fujii (Tokyo Medical University Hachioji Medical Center, Sub-investigator); Tsuyoshi Hashimoto (Tokyo Medical University Hachioji Medical Center, Sub-investigator); Magui Iioka (Tokyo Medical University Hachioji Medical Center, Sub-investigator); Minoru Sakai (Tokyo Medical University Hachioji Medical Center, Sub-investigator); Aki Shiina (Tokyo Medical University Hachioji Medical Center, Sub-investigator); Daisuke Suyama (Tokyo Medical University Hachioji Medical Center, Sub-investigator); Shingo Inoue (Tokyo Medical University Hachioji Medical Center, Sub-investigator); Daisuke Suyama (Tokyo Medical University Hachioji Medical Center, Sub-investigator); Hideki Takubo, MD (Ebara Hospital, PI); Takehiko Nagao (Ebara Hospital, Sub-investigator); Hiroshi Suwa (Ebara Hospital, Sub-investigator); Kazumasa Arii (Ebara Hospital, Sub-investigator); Keisuke Imai (Ebara Hospital, Sub-investigator); Kyugo Hirabayashi (Ebara Hospital, Sub-investigator); Masahiro Ida (Ebara Hospital, Sub-investigator); Masaya Okamura (Ebara Hospital, Sub-investigator); Masayuki Yokochi (Ebara Hospital, Sub-investigator); Nahoko Yoshimura (Ebara Hospital, Sub-investigator); Toshiyuki Otake (Ebara Hospital, Sub-investigator); Mari Inami (Ebara Hospital, Sub-investigator); Sayaka Suetake (Ebara Hospital, Sub-investigator); Yukiko Chiba (Ebara Hospital, Sub-investigator); Yukiko Furudate (Ebara Hospital, Sub-investigator); Yuichi Komaba, MD (Chiba Hokusoh Hospital, PI); Makoto Hamamoto (Chiba Hokusoh Hospital, Sub-investigator); Yusuke Toda (Chiba Hokusoh Hospital, Sub-investigator); Akiko Ozawa (Chiba Hokusoh Hospital, Sub-investigator); Makoto Sakurazawa (Chiba Hokusoh Hospital, Sub-investigator); Mina Harada (Chiba Hokusoh Hospital, Sub-investigator); Nobuo Kamiya (Chiba Hokusoh Hospital, Sub-investigator); Susumu Okada (Chiba Hokusoh Hospital, Sub-investigator); Yutaka Nishiyama (Chiba Hokusoh Hospital, Sub-investigator); Kei Usui (Chiba Hokusoh Hospital, Sub-investigator); Sachiyo Iizuka (Chiba Hokusoh Hospital, Sub-investigator); Yasuo Harigaya, MD (Maebashi Red Cross Hospital, PI); Hideo Morita (Maebashi Red Cross Hospital, Sub-investigator); Hiroshi Ishizaka (Maebashi Red Cross Hospital, Sub-investigator); Takemi Kurabayashi (Maebashi Red Cross Hospital, Sub-investigator); Kazuyuki Mizushima (Maebashi Red Cross Hospital, Sub-investigator); Kimitoshi Hirayanagi (Maebashi Red Cross Hospital, Sub-investigator); Natsumi Furuta (Maebashi Red Cross Hospital, Sub-investigator); Youko Obara (Maebashi Red Cross Hospital, Sub-investigator); Youko Tasaka (Maebashi Red Cross Hospital, Sub-investigator); Yumiko Motoi, MD (Juntendo University Hospital, PI); Ai Tan (Juntendo University Hospital, Sub-investigator); Ayaka Miyata (Juntendo University Hospital, Sub-investigator); Shigeto Sato (Juntendo University Hospital, Sub-investigator); Taiki Kambe (Juntendo University Hospital, Sub-investigator); Taku Hatano (Juntendo University Hospital, Sub-investigator); Hiromi Fujiwara (Juntendo University Hospital, Sub-investigator); Keiko Matsuo (Juntendo University Hospital, Sub-investigator); Hisashi Yonezawa, MD (Iwate Medical University Hospital, PI); Naoki Ishizuka (Iwate Medical University Hospital, Sub-investigator); Satoshi Takahashi (Iwate Medical University Hospital, Sub-investigator); Hisashi Yonezawa (Iwate Medical University Hospital, Sub-investigator); Junko Takahashi (Iwate Medical University Hospital, Sub-investigator); Masako Kudo (Iwate Medical University Hospital, Sub-investigator); Shigeru Ehara (Iwate Medical University Hospital, Sub-investigator); Hiromi Shiraishi (Iwate Medical University Hospital, Sub-investigator); Kuniko Ueno (Iwate Medical University Hospital, Sub-investigator); Nobuya Kawabata, MD, PhD (Yachiyo Hospital, PI); Kazuyuki Koyama (Yachiyo Hospital, Sub-investigator); Mariko Kitano (Yachiyo Hospital, Sub-investigator); Masahiro Yamazaki (Yachiyo Hospital, Sub-investigator); Yoshiyuki Okouchi (Yachiyo Hospital, Sub-investigator); Ayumi Tanaka (Yachiyo Hospital, Sub-investigator); Shinobu Hikosaka (Yachiyo Hospital, Sub-investigator); Shinji Ohara, MD (National Hospital Organization Matsumoto Medical Center, PI); Hiroshi Koshihara (National Hospital Organization Matsumoto Medical Center, Sub-investigator); Kenya Oguchi (National Hospital Organization Matsumoto Medical Center, Sub-investigator); Yoichi Takei (National Hospital Organization Matsumoto Medical Center, Sub-investigator); Hozumi Nomura (National Hospital Organization Matsumoto Medical Center, Sub-investigator); Shizuo Hatashita, MD (Shonan Atsugi Hospital, PI); Aki Uchida (Shonan Atsugi Hospital, Sub-investigator); Nobuhisa Tashiro (Shonan Atsugi Hospital, Sub-investigator); Hidetomo Yamasaki (Shonan Atsugi Hospital, Sub-investigator); Nobuaki Shinozaki (Shonan Atsugi Hospital, Sub-investigator); Takumi Sasaki (Shonan Atsugi Hospital, Sub-investigator); Kana Arita (Shonan Atsugi Hospital, Sub-investigator); Keiko Fusegi (Shonan Atsugi Hospital, Sub-investigator); Miwako Suzuki (Shonan Atsugi Hospital, Sub-investigator); Shoko Masuda (Shonan Atsugi Hospital, Sub-investigator); Taeko Hosono (Shonan Atsugi Hospital, Sub-investigator); Shin Kitamura, MD (Musashi Kosugi Hospital, PI); Eriko Yuba (Musashi Kosugi Hospital, Sub-investigator); Makoto Hamamoto (Musashi Kosugi Hospital, Sub-investigator); Yasuhiro Shimizu (Musashi Kosugi Hospital, Sub-investigator); Yoko Murano (Musashi Kosugi Hospital, Sub-investigator); Akiko Ishiwata (Musashi Kosugi Hospital, Sub-investigator); Manabu Yamamuro (Musashi Kosugi Hospital, Sub-investigator); Mariko Toyoshima (Musashi Kosugi Hospital, Sub-investigator); Yoshiaki Aihara, MD (Clinical Corporation Ikuseikai Shinozuka Hospital, PI); Yuki Imagawa (Clinical Corporation Ikuseikai Shinozuka Hospital, Sub-investigator); Makoto Tanaka (Clinical Corporation Ikuseikai Shinozuka Hospital, Sub-investigator); Yuko Aihara (Clinical Corporation Ikuseikai Shinozuka Hospital, Sub-investigator); Masaki Muranaka (Clinical Corporation Ikuseikai Shinozuka Hospital, Sub-investigator); Naoto Iwazaki (Clinical Corporation Ikuseikai Shinozuka Hospital, Sub-investigator); Ikumi Matsuzawa (Clinical Corporation Ikuseikai Shinozuka Hospital, Sub-investigator); Koichi Kashiwado, MD (Hakuyoukai Kashiwado Hospital, PI); Michie Miyoshi (Hakuyoukai Kashiwado Hospital, Sub-investigator); Toshihiro Saito (Hakuyoukai Kashiwado Hospital, Sub-investigator); Emi Arai (Hakuyoukai Kashiwado Hospital, Sub-investigator); Mami Kato (Hakuyoukai Kashiwado Hospital, Sub-investigator); Sachiyo Iizuka (Hakuyoukai Kashiwado Hospital, Sub-investigator); Yo Nishimura, MD (Nishi-Kobe Medical Center, PI); Chie Yanagihara (Nishi-Kobe Medical Center, Sub-investigator); Rieko Motooka (Nishi-Kobe Medical Center, Sub-investigator); Yoichiro Okada (Nishi-Kobe Medical Center, Sub-investigator); Yuko Wada (Nishi-Kobe Medical Center, Sub-investigator); Hiroko Taira (Nishi-Kobe Medical Center, Sub-investigator); Koichi Mino, MD (Kobe City Hospital Organization, PI); Yukihiro Takemura (Kobe City Hospital Organization, Sub-investigator); Keiko Miyake (Kobe City Hospital Organization, Sub-investigator); Maho Hattori (Kobe City Hospital Organization, Sub-investigator); Kaori Iwabuki (Kobe City Hospital Organization, Sub-investigator)

**Korea**

Ki Woong Kim, MD, PhD (Seoul National University Bundang Hospital, PI); Deok Ha Park (Seoul National University Bundang Hospital, Sub-investigator); In Yang Koo (Seoul National University Bundang Hospital, Sub-investigator); Jin Hee An (Seoul National University Bundang Hospital, Sub-investigator); Myeong Ju Pak (Seoul National University Bundang Hospital, Sub-investigator); Seon Young Yun (Seoul National University Bundang Hospital, Sub-investigator); Tae Hui Kim (Seoul National University Bundang Hospital, Sub-investigator); Jiwon Han (Seoul National University Bundang Hospital, Sub-investigator); Jin Yeong Choe (Seoul National University Bundang Hospital, Sub-investigator); Yeon Ja Do (Seoul National University Bundang Hospital, Sub-investigator); Seung Ho Ryu, MD, PhD (KonKuk University Medical Center, PI); Doo-Heum Park (KonKuk University Medical Center, Sub-investigator); Jaeeun Shin (KonKuk University Medical Center, Sub-investigator); Jaehak Yu (KonKuk University Medical Center, Sub-investigator); Jee Hyun Ha (KonKuk University Medical Center, Sub-investigator); Miyoo Cheon (KonKuk University Medical Center, Sub-investigator)

**Mexico**

Sarug Reyes Morales, MD (Instituto Biomédico de Investigación, A.C., PI); Angelica del Rosario Lopez Avalos (Instituto Biomédico de Investigación, A.C., Sub-investigator); Arlina Estrada-Valenciano (Instituto Biomédico de Investigación, A.C., Sub-investigator); Georgina Guadalupe Campos-Gallegos (Instituto Biomédico de Investigación, A.C., Sub-investigator); Ivan Wenceslao Leija Franco (Instituto Biomédico de Investigación, A.C., Sub-investigator); Mireya Vazquez Munoz (Instituto Biomédico de Investigación, A.C., Sub-investigator); Roberto Sanchez-Torre (Instituto Biomédico de Investigación, A.C., Sub-investigator); Erika Jimenez (Instituto Biomédico de Investigación, A.C., Sub-investigator); Hector Vazquez (Instituto Biomédico de Investigación, A.C., Sub-investigator); Jose Aleman (Instituto Biomédico de Investigación, A.C., Sub-investigator); Oscar Godoy Castaneda (Instituto Biomédico de Investigación, A.C., Sub-investigator); Salvador Perez-Jaime (Instituto Biomédico de Investigación, A.C., Sub-investigator); Santiago Paulino Ramirez-Diaz (Instituto Biomédico de Investigación, A.C., Sub-investigator); Juan Jose Esparza Correa (Instituto Biomédico de Investigación, A.C., Sub-investigator); Fabiola Ababel Juarez Medina (Instituto Biomédico de Investigación, A.C., Sub-investigator); Gabriela Martinez Muñoz, MD (OCA Hospital/Monterrey International Research Center, PI); Adrian De la Fuente-Cantu (OCA Hospital/Monterrey International Research Center, Sub-investigator); Albino Contreras (OCA Hospital/Monterrey International Research Center, Sub-investigator); Paola Rodriguez (OCA Hospital/Monterrey International Research Center, Sub-investigator); Sofia Siller (OCA Hospital/Monterrey International Research Center, Sub-investigator)

**Netherlands**

Evelien Lemstra (VUMC Amsterdam, Sub-investigator); W. de Haan (VUMC Amsterdam, Sub-investigator); Annelieke Fleuren (VUMC Amsterdam, Sub-investigator); I. E.W. Reuling (VUMC Amsterdam, Sub-investigator); L. Smits (VUMC Amsterdam, Sub-investigator); N. Prins (VUMC Amsterdam, Sub-investigator); N. Sistermans (VUMC Amsterdam, Sub-investigator); P. J. Visser (VUMC Amsterdam, Sub-investigator); Hoda Rezaei (VUMC Amsterdam, Sub-investigator); Paul L.J. Dautzenberg, MD, PhD (Jeroen Bosch Ziekenhuis, PI); Jolanda H.M. van de Kerkhof (Jeroen Bosch Ziekenhuis, Sub-investigator); Cees Wouters (Jeroen Bosch Ziekenhuis, Sub-investigator); Nelly W. Wouters-Van Ooyen (Jeroen Bosch Ziekenhuis, Sub-investigator); Leo Boelaarts, MD (Medical Center Alkmaar, PI); M. Blom (Medical Center Alkmaar, Sub-investigator); J. F.M. de Jonghe (Medical Center Alkmaar, Sub-investigator); J. Peetoom (Medical Center Alkmaar, Sub-investigator); Jules J. Claus, MD, PhD (Tergooi Ziekenhuizen Hilversum, PI); Dirk Hederschee (Tergooi Ziekenhuizen Hilversum, Sub-investigator); Martijn Stevens (Tergooi Ziekenhuizen Hilversum, Sub-investigator); P.M.M. Van Erven, MD, PhD (Amphia Ziekenhuis, PI); Johanna F. De Rijkvan van Andel (Amphia Ziekenhuis, Sub-investigator); V. van Oers (Amphia Ziekenhuis, Sub-investigator); E.A.C.M. Sanders, MD, PhD (Amphia Ziekenhuis, PI); Dieneke Z.B. Van Asselt, MD, PhD (Medisch Centrum Leeuwarden, PI); R. Koffijberg (Medisch Centrum Leeuwarden, Sub-investigator); A. Kwast (Medisch Centrum Leeuwarden, Sub-investigator); Arjan Minneboo (Medisch Centrum Leeuwarden, Sub-investigator); M. van Dommele (Medisch Centrum Leeuwarden, Sub-investigator); L. van Houte (Medisch Centrum Leeuwarden, Sub-investigator); P. van Walderveen (Medisch Centrum Leeuwarden, Sub-investigator); John C. Van Swieten, MD, PhD (Erasmus Medical Center Rotterdam, PI); W. Z. Chiu (Erasmus Medical Center Rotterdam, Sub-investigator); R. de Graaf (Erasmus Medical Center Rotterdam, Sub-investigator); I. de Koning (Erasmus Medical Center Rotterdam, Sub-investigator); Cees Wouters, MD (Jeroen Bosch Ziekenhuis, PI); Jolanda H.M. van de Kerkhof (Jeroen Bosch Ziekenhuis, Sub-investigator); Nelly W. Wouters-Van Ooyen (Jeroen Bosch Ziekenhuis, Sub-investigator); Kees Kalisvaart, MD, PhD (Kennemer Gasthuis, PI); A. Spilt (Kennemer Gasthuis, Sub-investigator); Gisela Dekker (Kennemer Gasthuis, Sub-investigator); Milko Van Langen (Kennemer Gasthuis, Sub-investigator); Gerrit Jan Hafkamp (Kennemer Gasthuis, Sub-investigator); Koos Keizer, MD, PhD (Catharina Ziekenhuis, PI); A. A.M. Nusselein (Catharina Ziekenhuis, Sub-investigator); P. Lenders (Catharina Ziekenhuis, Sub-investigator); S. C. Dijksman (Catharina Ziekenhuis, Sub-investigator); Femke Bouwman, MD, PhD (Catharina Ziekenhuis, PI)

**New Zealand**

John R. Elliot, MBChB, FRACP (Signet Research Ltd., PI); Anna Jane Fenton (Signet Research Ltd., Sub-investigator); Brian John Deavoll (Signet Research Ltd., Sub-investigator); Averly Andrews (Signet Research Ltd., Sub-investigator); Deborah Snell (Signet Research Ltd., Sub-investigator); Philip Clive Wood, MBChB, FRACP (Waikato Hospital, PI); Jane Bryce (Waikato Hospital, Sub-investigator); Lorna Crawford (Waikato Hospital, Sub-investigator); Mary Manson Lythe (Waikato Hospital, Sub-investigator); Alexander Joseph Srzich (Waikato Hospital, Sub-investigator); Patricia Sullivan (Waikato Hospital, Sub-investigator)

**Poland**

Jan Ilkowski, MD (NZOZ “Neuro-Kard,” PI); Maria Westerska (NZOZ “Neuro-Kard,” Sub-investigator); Anna Lewandowska (NZOZ “Neuro-Kard,” Sub-investigator); Lucyna Baszkiewicz (NZOZ “Neuro-Kard,” Sub-investigator); Magdalena Starkowska (NZOZ “Neuro-Kard,” Sub-investigator); Malgorzata Oleskowicz-Popiel (NZOZ “Neuro-Kard,” Sub-investigator); Anna Dabrowska (NZOZ “Neuro-Kard,” Sub-investigator); Dobroslawa Kwiatkowska-Kusnierek (NZOZ “Neuro-Kard,” Sub-investigator); Dorota Gulczynska (NZOZ “Neuro-Kard,” Sub-investigator); Jolanta Jujka (NZOZ “Neuro-Kard,” Sub-investigator); Piotr Sosnowski (NZOZ “Neuro-Kard,” Sub-investigator); Arleta Kuczynska-Zardzewialy, MD (SPZOZ Szpital Wolski im. Dr Anny Gostynskiej, PI); Hanna Bragoszowska (SPZOZ Szpital Wolski im. Dr Anny Gostynskiej, Sub-investigator); Agnieszka Gutkiewicz (SPZOZ Szpital Wolski im. Dr Anny Gostynskiej, Sub-investigator); Anna Lewandowska (SPZOZ Szpital Wolski im. Dr Anny Gostynskiej, Sub-investigator); Grazyna Miach-Uchman (SPZOZ Szpital Wolski im. Dr Anny Gostynskiej, Sub-investigator); Barbara Pomianowska (SPZOZ Szpital Wolski im. Dr Anny Gostynskiej, Sub-investigator); Beata Papierowska (SPZOZ Szpital Wolski im. Dr Anny Gostynskiej, Sub-investigator); Robert Kucharski, MD (NZOZ Dom Sue Ryder, PALLMED Sp. z o.o., PI); Paulina Andryszak (NZOZ Dom Sue Ryder, PALLMED Sp. z o.o., Sub-investigator); Kinga Jochim (NZOZ Dom Sue Ryder, PALLMED Sp. z o.o., Sub-investigator); Katarzyna Lachut (NZOZ Dom Sue Ryder, PALLMED Sp. z o.o., Sub-investigator); Maria Nowak (NZOZ Dom Sue Ryder, PALLMED Sp. z o.o., Sub-investigator); Hanna Rutkowska (NZOZ Dom Sue Ryder, PALLMED Sp. z o.o., Sub-investigator); Aleksandra Seredyka (NZOZ Dom Sue Ryder, PALLMED Sp. z o.o., Sub-investigator); Mariusz Wachulski (NZOZ Dom Sue Ryder, PALLMED Sp. z o.o., Sub-investigator); Marcin Wozniak (NZOZ Dom Sue Ryder, PALLMED Sp. z o.o., Sub-investigator); Marzena Ziolkowska-Kochan (NZOZ Dom Sue Ryder, PALLMED Sp. z o.o., Sub-investigator); Malgorzata Gawel, MD, PhD (Samodzielny Publiczny Centralny Szpital Kliniczny, PI); Andrzej Cieszanowski (Samodzielny Publiczny Centralny Szpital Kliniczny, Sub-investigator); Marek Golebiowski (Samodzielny Publiczny Centralny Szpital Kliniczny, Sub-investigator); Krystyna Gospodarczyk-Szot (Samodzielny Publiczny Centralny Szpital Kliniczny, Sub-investigator); Irena Jalenska-Szygula (Samodzielny Publiczny Centralny Szpital Kliniczny, Sub-investigator); Izabela Jelenska-Szygula (Samodzielny Publiczny Centralny Szpital Kliniczny, Sub-investigator); Justyna Kubiszewska (Samodzielny Publiczny Centralny Szpital Kliniczny, Sub-investigator); Edyta Maj (Samodzielny Publiczny Centralny Szpital Kliniczny, Sub-investigator); Monika Nardzenska-Szczepanik (Samodzielny Publiczny Centralny Szpital Kliniczny, Sub-investigator); Anna Paciorek (Samodzielny Publiczny Centralny Szpital Kliniczny, Sub-investigator); Beata Pilczuk (Samodzielny Publiczny Centralny Szpital Kliniczny, Sub-investigator); Anna Potulska-Chromik (Samodzielny Publiczny Centralny Szpital Kliniczny, Sub-investigator); Olgierd Rowinski (Samodzielny Publiczny Centralny Szpital Kliniczny, Sub-investigator); Martyna Sitek (Samodzielny Publiczny Centralny Szpital Kliniczny, Sub-investigator); Elzbieta Szmidt-Salkowska (Samodzielny Publiczny Centralny Szpital Kliniczny, Sub-investigator); Andrzej Urbanik (Samodzielny Publiczny Centralny Szpital Kliniczny, Sub-investigator); Wadim Wojiechowski (Samodzielny Publiczny Centralny Szpital Kliniczny, Sub-investigator); Hubert Kwieciński, MD, PhD (Samodzielny Publiczny Centralny Szpital Kliniczny, PI); Alicja Klich-Raczka, MD, DPhil (Szpital Uniwersytecki w Krakowie, PI); Barbara Wizner (Szpital Uniwersytecki w Krakowie, Sub-investigator); Karolina Piotrowicz (Szpital Uniwersytecki w Krakowie, Sub-investigator); Malgorzata Stompor (Szpital Uniwersytecki w Krakowie, Sub-investigator); Melania Pitucha (Szpital Uniwersytecki w Krakowie, Sub-investigator); Barbara Wizner (Szpital Uniwersytecki w Krakowie, Sub-investigator); Karolina Piotrowicz (Szpital Uniwersytecki w Krakowie, Sub-investigator); Malgorzata Stompor (Szpital Uniwersytecki w Krakowie, Sub-investigator); Melania Pitucha (Szpital Uniwersytecki w Krakowie, Sub-investigator); Pawel Pólrola, MD, PhD (NZOZ “SYNAPSA,” PI); Danuta Glanda (NZOZ “SYNAPSA,” Sub-investigator); Marek Cwiakala (NZOZ “SYNAPSA,” Sub-investigator); Slawomir Plewa (NZOZ “SYNAPSA,” Sub-investigator); Izabela Chojnowska Cwiakala (NZOZ “SYNAPSA,” Sub-investigator)

**Portugal**

Ana Herrero Valverde, MD, PhD (Hospital Fernando Fonseca, PI); Ana Paula Silva (Hospital Fernando Fonseca, Sub-investigator); Andre Miguel Carvalho (Hospital Fernando Fonseca, Sub-investigator); Antonio Vasco Sande e Castro Salgado (Hospital Fernando Fonseca, Sub-investigator); Carina Cristina Carvalho Gonçalves Alves (Hospital Fernando Fonseca, Sub-investigator); Elsa Paixao Parreira (Hospital Fernando Fonseca, Sub-investigator); Angela Timoteo (Hospital Fernando Fonseca, Sub-investigator); Cristina Costa (Hospital Fernando Fonseca, Sub-investigator); Nuno Inacio (Hospital Fernando Fonseca, Sub-investigator); Sonia Costa (Hospital Fernando Fonseca, Sub-investigator); Sara Machado (Hospital Fernando Fonseca, Sub-investigator); Tiago Mendes (Hospital Fernando Fonseca, Sub-investigator); Catarina Castro (Hospital Fernando Fonseca, Sub-investigator); Rita Simoes (Hospital Fernando Fonseca, Sub-investigator); Luis Cunha, MD, PhD (Hospitais da Universidade de Coimbra, PI); Margarida Filipa Fonseca Vicente (Hospitais da Universidade de Coimbra, Sub-investigator); Ana Roigues (Hospitais da Universidade de Coimbra, Sub-investigator); Catarina Cunha (Hospitais da Universidade de Coimbra, Sub-investigator); Raquel Lemos (Hospitais da Universidade de Coimbra, Sub-investigator); Beatriz Santiago (Hospitais da Universidade de Coimbra, Sub-investigator); Isabel Santana (Hospitais da Universidade de Coimbra, Sub-investigator); Alexandre Mendonça, MD, PhD (Hospital Santa Maria, PI); Clara Loureiro (Hospital Santa Maria, Sub-investigator); Catarina Chester (Hospital Santa Maria, Sub-investigator); Manuela Guerreiro (Hospital Santa Maria, Sub-investigator); Frederico Couto (Hospital Santa Maria, Sub-investigator); Ana Verdelho (Hospital Santa Maria, Sub-investigator)

**Russian Federation**

Oleg Anatolievich Balunov, MD, PhD (Saint Petersburg Psychoneurological Research Institute n.a. V.M. Bekhterev of Roszdrav, PI); Denis Zakharov (Saint Petersburg Psychoneurological Research Institute n.a. V.M. Bekhterev of Roszdrav, Sub-investigator); Julia V Kotsubinskaya (Saint Petersburg Psychoneurological Research Institute n.a. V.M. Bekhterev of Roszdrav, Sub-investigator); Natalia V. Safonova (Saint Petersburg Psychoneurological Research Institute n.a. V.M. Bekhterev of Roszdrav, Sub-investigator); Eduard Zakirzianovich Yakupov, MD, PhD (Scientific Research Medical Complex Vashe Zdorovie, PI); Yulia Zhitkova (Scientific Research Medical Complex Vashe Zdorovie, Sub-investigator); Leisan Kamalova (Scientific Research Medical Complex Vashe Zdorovie, Sub-investigator); Natalya Vasiljevna Agafonova (Scientific Research Medical Complex Vashe Zdorovie, Sub-investigator); Elvira R Gizatullina (Scientific Research Medical Complex Vashe Zdorovie, Sub-investigator); Nikolay Neznanov, MD (Saint Petersburg Psychoneurological Research Institute n.a. V.M. Bekhterev of Roszdrav, PI); Elena Zadorozhnaya (Saint Petersburg Psychoneurological Research Institute n.a. V.M. Bekhterev of Roszdrav, Sub-investigator); Natalia Zalutskaya (Saint Petersburg Psychoneurological Research Institute n.a. V.M. Bekhterev of Roszdrav, Sub-investigator); Svetlana Ephremovna Tatulyan (Saint Petersburg Psychoneurological Research Institute n.a. V.M. Bekhterev of Roszdrav, Sub-investigator); Tatiana Apraksina (Saint Petersburg Psychoneurological Research Institute n.a. V.M. Bekhterev of Roszdrav, Sub-investigator); Zhanna Zheltyakova (Saint Petersburg Psychoneurological Research Institute n.a. V.M. Bekhterev of Roszdrav, Sub-investigator); Miroslav Mikhailovich Odinak, MD, PhD (Military Medical Academy n.a. S.M. Kirov of Ministry of Defense of Russia, PI); Andrey Kashin (Military Medical Academy n.a. S.M. Kirov of Ministry of Defense of Russia, Sub-investigator); Andrey Yurievich Emelin (Military Medical Academy n.a. S.M. Kirov of Ministry of Defense of Russia, Sub-investigator); Sergey Vladimirovich Vorobyev (Military Medical Academy n.a. S.M. Kirov of Ministry of Defense of Russia, Sub-investigator); Vladimir Yurievich Lobzin (Military Medical Academy n.a. S.M. Kirov of Ministry of Defense of Russia, Sub-investigator); Sofia F. Sluchevskaya, MD (Saint-Petersburg State Institution of Healthcare, PI); Maya Nikolaeva (Saint-Petersburg State Institution of Healthcare, Sub-investigator); Andrey Georgievich Samokhin (Saint-Petersburg State Institution of Healthcare, Sub-investigator); Elena Galubeva (Saint-Petersburg State Institution of Healthcare, Sub-investigator); Inna Kiseleva (Saint-Petersburg State Institution of Healthcare, Sub-investigator); Lilia Viktorovna Lenskaya (Saint-Petersburg State Institution of Healthcare, Sub-investigator); Maxim Gordeev (Saint-Petersburg State Institution of Healthcare, Sub-investigator); Natalia Georgievna Malyavina (Saint-Petersburg State Institution of Healthcare, Sub-investigator); Alexander V. Gustov, MD, PhD (State Institution of Healthcare Nizhny Novgorod Regional Clinical Hospital n.a. N.A. Semashko, PI); Svetlana Kopishinskaya (State Institution of Healthcare Nizhny Novgorod Regional Clinical Hospital n.a. N.A. Semashko, Sub-investigator); Ekaterina A. Alexandrova (State Institution of Healthcare Nizhny Novgorod Regional Clinical Hospital n.a. N.A. Semashko, Sub-investigator); Elena A. Antipenko (State Institution of Healthcare Nizhny Novgorod Regional Clinical Hospital n.a. N.A. Semashko, Sub-investigator); Maria B. Karpukhina (State Institution of Healthcare Nizhny Novgorod Regional Clinical Hospital n.a. N.A. Semashko, Sub-investigator); Viktoria A. Antonova (State Institution of Healthcare Nizhny Novgorod Regional Clinical Hospital n.a. N.A. Semashko, Sub-investigator); Zhanna V. Romanenko (State Institution of Healthcare Nizhny Novgorod Regional Clinical Hospital n.a. N.A. Semashko, Sub-investigator)

**Serbia**

Svetlana Miletic Drakulic, MD, Ass. PhD (Clinical Centre Kragujevac, PI); Vladimir Kostic, MD, PhD (Klinicki centar Srbije, PI); Edita Cvitan (Klinicki centar Srbije, Sub-investigator); Eleonora Dzoljic (Klinicki centar Srbije, Sub-investigator); Mihail Gavrilov (Klinicki centar Srbije, Sub-investigator); Slobodan Lavrnic (Klinicki centar Srbije, Sub-investigator); Milica Lukic Jecmenica (Klinicki centar Srbije, Sub-investigator); Gorana Mandic (Klinicki centar Srbije, Sub-investigator); Tanja Stosic Opincal (Klinicki centar Srbije, Sub-investigator); Igor Petrovic (Klinicki centar Srbije, Sub-investigator); Vladana Spica (Klinicki centar Srbije, Sub-investigator); Elka Stefanova (Klinicki centar Srbije, Sub-investigator); Tanja Stojkovic (Klinicki centar Srbije, Sub-investigator); Marina Svetel (Klinicki centar Srbije, Sub-investigator); Aleksandra Tomic (Klinicki centar Srbije, Sub-investigator); Tatjana Stosic Opincal (Klinicki centar Srbije, Sub-investigator); Vesna Divac (Klinicki centar Srbije, Sub-investigator); Zorica Knezevic, MD, MSc (Klinicki centar Kragujevac, PI); Tatjana Boskovic Matic (Klinicki centar Kragujevac, Sub-investigator); Milan Mijailovic (Klinicki centar Kragujevac, Sub-investigator); Mirjana Petrovic (Klinicki centar Kragujevac, Sub-investigator); Zorica Smiljanic (Klinicki centar Kragujevac, Sub-investigator); Gordana Toncev (Klinicki centar Kragujevac, Sub-investigator); Gordana Zlatic (Klinicki centar Kragujevac, Sub-investigator); Svetlana Miletic Drakulic (Klinicki centar Kragujevac, Sub-investigator); Marija Semnic, MD, PhD (Klinicki centar Vojvodina, PI); Vojislava Bugarski (Klinicki centar Vojvodina, Sub-investigator); Dunja Cigic (Klinicki centar Vojvodina, Sub-investigator); Nenad Delibasic (Klinicki centar Vojvodina, Sub-investigator); Olga Horvat (Klinicki centar Vojvodina, Sub-investigator); Aleksandar Jesic (Klinicki centar Vojvodina, Sub-investigator); Nemanja Popvic (Klinicki centar Vojvodina, Sub-investigator); Ljiljana Ruzic (Klinicki centar Vojvodina, Sub-investigator); Robert Semnic (Klinicki centar Vojvodina, Sub-investigator); Dragana Stefanovic (Klinicki centar Vojvodina, Sub-investigator); Anastazija Stojsi Milosavljevic (Klinicki centar Vojvodina, Sub-investigator); Lorand Szakallas (Klinicki centar Vojvodina, Sub-investigator)

**Slovakia**

Peter Korcsog, MD (General Hospital Rimavska Sobota, PI); Abdul Mohammad Shinwari (General Hospital Rimavska Sobota, Sub-investigator); Peter Strecka (General Hospital Rimavska Sobota, Sub-investigator); Robert Sliva (General Hospital Rimavska Sobota, Sub-investigator); Izabela Mátéffy, MD (Faculty Hospital Bratislava, PI); Dagmar Dziakova (Faculty Hospital Bratislava, Sub-investigator); Daniel Petersky (Faculty Hospital Bratislava, Sub-investigator); Miroslav Satko (Faculty Hospital Bratislava, Sub-investigator); Martin Kalas (Faculty Hospital Bratislava, Sub-investigator); Zuzana Zacharova (Faculty Hospital Bratislava, Sub-investigator); Katarina Edelova (Faculty Hospital Bratislava, Sub-investigator); Monika Biackova, MD (Psychiatric Hospital Michalovce, PI); Danica Korpova (Psychiatric Hospital Michalovce, Sub-investigator); Erika Rusinova (Psychiatric Hospital Michalovce, Sub-investigator); Pavol Balasic (Psychiatric Hospital Michalovce, Sub-investigator); Peter Molčan, MD (Ružinovská poliklinika, PI); Silvia Bogyaiova (Ružinovská poliklinika, Sub-investigator); Dagmar Dziakova (Ružinovská poliklinika, Sub-investigator); Daniel Petersky (Ružinovská poliklinika, Sub-investigator); Miroslav Satko (Ružinovská poliklinika, Sub-investigator); Frantisek Jurcaga, MD (Neurologicka klinika SZU, PI); Andrea Hergottova (Neurologicka klinika SZU, Sub-investigator); Karin Pribisova (Neurologicka klinika SZU, Sub-investigator); Peter Koson (Neurologicka klinika SZU, Sub-investigator); Anetta Kovacikova (Neurologicka klinika SZU, Sub-investigator); Egon Kurča, MD, PhD (Martinska Fakultna nemocnica, PI); Slavka Jasekova (Martinska Fakultna nemocnica, Sub-investigator); Daniela Sutorova (Martinska Fakultna nemocnica, Sub-investigator); Milan Grofik (Martinska Fakultna nemocnica, Sub-investigator); Stefan Sivak (Martinska Fakultna nemocnica, Sub-investigator); Vladimir Nosal (Martinska Fakultna nemocnica, Sub-investigator); Peter Turcani, MD, PhD (Univerzitna nemocnica Bratislava, PI); Monika Dolezalova (Univerzitna nemocnica Bratislava, Sub-investigator); Jana Borovska (Univerzitna nemocnica Bratislava, Sub-investigator); Marian Kondas (Univerzitna nemocnica Bratislava, Sub-investigator); Stanislav Sutovsky (Univerzitna nemocnica Bratislava, Sub-investigator); Juraj Vyletelka, MD (Faculty Hospital Zilina, PI); Barbora Hanova (Faculty Hospital Zilina, Sub-investigator); Julia Poljakova (Faculty Hospital Zilina, Sub-investigator); Miroslav Vastik (Faculty Hospital Zilina, Sub-investigator); Peter Hano (Faculty Hospital Zilina, Sub-investigator); Silvie Kuzmova (Faculty Hospital Zilina, Sub-investigator)

**South Africa**

Judy Green, MBChB (St. Augustine’s Hospital, PI); Farhana Motala (St. Augustine’s Hospital, Sub-investigator); Michael Shane Mason, MBChB (Panorama Psychiatry and Memory Clinic, PI); Paul Magni (Panorama Psychiatry and Memory Clinic, Sub-investigator); Azelle Antoinette Mayne (Panorama Psychiatry and Memory Clinic, Sub-investigator); Stanley Lipschitz, MBChB (The Osteoporosis Clinic and Memory Centre, PI); Hendi Bekker (The Osteoporosis Clinic and Memory Centre, Sub-investigator); Karin Kopenhager (The Osteoporosis Clinic and Memory Centre, Sub-investigator); Felix Claude Victor Potocnik, MD (Flexivest Fourteen Research Centre, PI); Dana Niehaus (Flexivest Fourteen Research Centre, Sub-investigator); Salumu Selemani, MBChB (Rand Clinic Johannesburg, PI); Nyembue Kazadi (Rand Clinic Johannesburg, Sub-investigator); Gert P. Bosch, MBChB (Denmar Psychiatric Hospital, PI); Irma Verster (Denmar Psychiatric Hospital, Sub-investigator); Renata du Preez (Denmar Psychiatric Hospital, Sub-investigator); Desiree Rossouw (Denmar Psychiatric Hospital, Sub-investigator)

**Spain**

Asuncion Lafuente Rodes (Fundació ACE, Sub-investigator); Cesar Morcillo Serra (Fundació ACE, Sub-investigator); Irene Rosell Abril (Fundació ACE, Sub-investigator); Isabel Hernandez Ruiz (Fundació ACE, Sub-investigator); Mar Lara (Fundació ACE, Sub-investigator); Marina Guitart Pinedo (Fundació ACE, Sub-investigator); Marta Ibarria Sala (Fundació ACE, Sub-investigator); Pablo Martinez Lage (Fundació ACE, Sub-investigator); Susana Diego Gullon (Fundació ACE, Sub-investigator); Jordi Peña-Casanova, MD (Hospital del Mar, PI); Joao Pedro Morais Ribeira (Hospital del Mar, Sub-investigator); Lluis Planellas Gine (Hospital del Mar, Sub-investigator); Monica Tolsanas (Hospital del Mar, Sub-investigator); Sara Carillo Molina (Hospital del Mar, Sub-investigator); Sonia Quinones Ubeda (Hospital del Mar, Sub-investigator); Susana De Sola Llopis (Hospital del Mar, Sub-investigator); Gracia Cucurella Montane (Hospital del Mar, Sub-investigator); Gonzalo Sanchez Benavides (Hospital del Mar, Sub-investigator); Marta Casals Coll (Hospital del Mar, Sub-investigator); Rafael Blesa, MD, PhD (Hospital de la Santa Creu i Sant Pau, PI); Amparo Villar Canovas (Hospital de la Santa Creu i Sant Pau, Sub-investigator); Isabel Sala Matavera (Hospital de la Santa Creu i Sant Pau, Sub-investigator); Maria Belen Sanchez Saudinos (Hospital de la Santa Creu i Sant Pau, Sub-investigator); Maria Carmona Iragui (Hospital de la Santa Creu i Sant Pau, Sub-investigator); Sofia Anton Aguirre (Hospital de la Santa Creu i Sant Pau, Sub-investigator); Anna Maria Pujol Nuez (Hospital de la Santa Creu i Sant Pau, Sub-investigator); Maria del Pilar Sainz Pelayo (Hospital de la Santa Creu i Sant Pau, Sub-investigator); Rosa Maria Antonijoan Arbos (Hospital de la Santa Creu i Sant Pau, Sub-investigator); Carme Garcia (Hospital de la Santa Creu i Sant Pau, Sub-investigator); Miguel Aguilar Barbera, MD, PhD (Hospital Mutua de Terrassa, PI); Heiko Wegrich (Hospital Mutua de Terrassa, Sub-investigator); Jorge de Francisco Moure (Hospital Mutua de Terrassa, Sub-investigator); Lorena Garcia Ortiz (Hospital Mutua de Terrassa, Sub-investigator); Pilar Quilez Ferrer (Hospital Mutua de Terrassa, Sub-investigator); Dolores Badenes Guia (Hospital Mutua de Terrassa, Sub-investigator); Laura Casas Hernanz (Hospital Mutua de Terrassa, Sub-investigator); Noemi Calzado Martinez (Hospital Mutua de Terrassa, Sub-investigator); Ana Frank Garcia, MD, PhD (Hospital Universitario La Paz, PI); Carolina Saenz Lafourcade (Hospital Universitario La Paz, Sub-investigator); Jesus Maria Lopez Arrieta (Hospital Universitario La Paz, Sub-investigator); Marcos Llanero (Hospital Universitario La Paz, Sub-investigator); Rocio Garcia Cobos (Hospital Universitario La Paz, Sub-investigator); Antonio Tallon (Hospital Universitario La Paz, Sub-investigator); Arturo Sampedro (Hospital Universitario La Paz, Sub-investigator); Maria Ascencion Zea (Hospital Universitario La Paz, Sub-investigator); Meritxel Valenti (Hospital Universitario La Paz, Sub-investigator); Ana Simon (Hospital Universitario La Paz, Sub-investigator); Manuel Lara Lara (Hospital Universitario La Paz, Sub-investigator); Clarissa Vincent Lazaro (Hospital Universitario La Paz, Sub-investigator); Genny Lubrini (Hospital Universitario La Paz, Sub-investigator); Miguel Goñi Imizcoz, MD (Complejo Asistencial de Burgos, PI); Nicolas Herrera Varo (Complejo Asistencial de Burgos, Sub-investigator); Fernando Iglesias Diez (Complejo Asistencial de Burgos, Sub-investigator); Francisco Javier Moreno Martinez (Complejo Asistencial de Burgos, Sub-investigator); Félix Bermejo Pareja, MD, PhD (Hospital Universitario 12 de Octubre, PI); Irene Fernandez Herraez (Hospital Universitario 12 de Octubre, Sub-investigator); Alberto Villarejo Galende (Hospital Universitario 12 de Octubre, Sub-investigator); Alvaro Sanchez Ferro (Hospital Universitario 12 de Octubre, Sub-investigator); Carolina Vera Garcia (Hospital Universitario 12 de Octubre, Sub-investigator); Veronica Puertas Martin (Hospital Universitario 12 de Octubre, Sub-investigator); Manuel Fernández Martínez, MD, PhD (Hospital de Cruces, PI); Rocio Bereincua (Hospital de Cruces, Sub-investigator); Jose Losada (Hospital de Cruces, Sub-investigator); Ana Molano (Hospital de Cruces, Sub-investigator); Pedro Gil Gregorio, MD (Hospital Universitario Clínico San Carlos, PI); Marta Vigara Garcia (Hospital Universitario Clínico San Carlos, Sub-investigator); Alan Albarracin Delgado (Hospital Universitario Clínico San Carlos, Sub-investigator); Aurora Viloria Jimenez (Hospital Universitario Clínico San Carlos, Sub-investigator); Carmen Pablos (Hospital Universitario Clínico San Carlos, Sub-investigator); Claudia Teran Benzaquen (Hospital Universitario Clínico San Carlos, Sub-investigator); Francisco Soria (Hospital Universitario Clínico San Carlos, Sub-investigator); Luis Donis (Hospital Universitario Clínico San Carlos, Sub-investigator); Marisa Covarrubias Esquer (Hospital Universitario Clínico San Carlos, Sub-investigator); Miriam Ramos (Hospital Universitario Clínico San Carlos, Sub-investigator); Monica Chung Jaen (Hospital Universitario Clínico San Carlos, Sub-investigator); Morclo Leonilde (Hospital Universitario Clínico San Carlos, Sub-investigator); Isabel Cruz Orduña (Hospital Universitario Clínico San Carlos, Sub-investigator); Jara Velasco Garcia-Cuevas (Hospital Universitario Clínico San Carlos, Sub-investigator); Raquel Yubero Pancorvo (Hospital Universitario Clínico San Carlos, Sub-investigator); Jose Aurelio Vivancos Mora, MD, PhD (Hospital Universitario La Princesa, PI); Ana Beatriz Gago Veiga (Hospital Universitario La Princesa, Sub-investigator); Antonio Ortiz Pascual (Hospital Universitario La Princesa, Sub-investigator); Teresa Carreras Rodriguez (Hospital Universitario La Princesa, Sub-investigator); Virginia Meca Lallana (Hospital Universitario La Princesa, Sub-investigator); Miguel Ruiz Muñoz-Torrero (Hospital Universitario La Princesa, Sub-investigator); Carmen Onsurbe Dominguez (Hospital Universitario La Princesa, Sub-investigator); Jordi Alom, MD, PhD (Hospital General Universitario de Elche, PI); Maria Alvarez (Hospital General Universitario de Elche, Sub-investigator); Miguel Angel Garcia Quesada (Hospital General Universitario de Elche, Sub-investigator); Isabel Llinares (Hospital General Universitario de Elche, Sub-investigator); Carmen Antunez, MD (Hospital Universitario Virgen de la Arrixaca, PI); Juan Marin Muñoz (Hospital Universitario Virgen de la Arrixaca, Sub-investigator); Maria Fuensanta Noguera (Hospital Universitario Virgen de la Arrixaca, Sub-investigator); Begona Martinez (Hospital Universitario Virgen de la Arrixaca, Sub-investigator); Laura Vivancos (Hospital Universitario Virgen de la Arrixaca, Sub-investigator); Salvadora Manzanares (Hospital Universitario Virgen de la Arrixaca, Sub-investigator); Maria Martirio Antequera (Hospital Universitario Virgen de la Arrixaca, Sub-investigator); Fernando Castellanos Pinedo, MD (Hospital Virgen del Puerto, PI); Beatriz Rodriguez Funez (Hospital Virgen del Puerto, Sub-investigator); Jose Maria Hernandez Perez (Hospital Virgen del Puerto, Sub-investigator); Jose Martin Zurdo Hernandez (Hospital Virgen del Puerto, Sub-investigator); Ciara Garcia Fernandez (Hospital Virgen del Puerto, Sub-investigator); Justo Garcia de Yebenes Prous, MD (Hospital Universitario Ramón y Cajal, PI); Juan Garcia Caldentey (Hospital Universitario Ramón y Cajal, Sub-investigator); Guillermo Garcia-Ribas (Hospital Universitario Ramón y Cajal, Sub-investigator); Jose Luis Lopez-Sendon Moreno (Hospital Universitario Ramón y Cajal, Sub-investigator); Marta Fatas (Hospital Universitario Ramón y Cajal, Sub-investigator); Monica Bascunana Garde (Hospital Universitario Ramón y Cajal, Sub-investigator); Patricia Esther Trigo Cubillo (Hospital Universitario Ramón y Cajal, Sub-investigator); Lucia Esteban (Hospital Universitario Ramón y Cajal, Sub-investigator); Adolfo López de Munain, MD, PhD (Hospital Universitario Donostia, PI); Ana Belen Asensio (Hospital Universitario Donostia, Sub-investigator); Myriam Barandiaran (Hospital Universitario Donostia, Sub-investigator); Ainara Estanga (Hospital Universitario Donostia, Sub-investigator); Begona Indacoechea (Hospital Universitario Donostia, Sub-investigator); Fermin Moreno Izco (Hospital Universitario Donostia, Sub-investigator); Andone Sistiaga (Hospital Universitario Donostia, Sub-investigator); Guillermo Amer Ferrer, MD (Hospital Universitario Son Espases, PI); Anna Maria Pujol Nuez (Hospital Universitario Son Espases, Sub-investigator); Vanessa Nunez Gutierrez (Hospital Universitario Son Espases, Sub-investigator); Ana Maria Garcia Martin (Hospital Universitario Son Espases, Sub-investigator); Francisco Javier Robles Mateos (Hospital Universitario Son Espases, Sub-investigator); Serafin Jimenez Rodriguez (Hospital Universitario Son Espases, Sub-investigator); Barbara Vives Pastor (Hospital Universitario Son Espases, Sub-investigator); Catalina Llompart Mateu (Hospital Universitario Son Espases, Sub-investigator); Helena Vico Bondia (Hospital Universitario Son Espases, Sub-investigator); Susana Tarongi Sanchez (Hospital Universitario Son Espases, Sub-investigator)

**Sweden**

Henrik Östlund, MD (Skånes Universitetssjukhus, PI); Cecilia Persson (Skånes Universitetssjukhus, Sub-investigator); Lena Ek (Skånes Universitetssjukhus, Sub-investigator); Mikael Nornholm (Skånes Universitetssjukhus, Sub-investigator); Susanne Svensson (Skånes Universitetssjukhus, Sub-investigator); Eva Bergstrom Mandl (Skånes Universitetssjukhus, Sub-investigator); Gunnar Kol (Skånes Universitetssjukhus, Sub-investigator); Martin Ingelsson, MD, PhD (Uppsala Universitet, PI); Birgitta Ausen (Uppsala Universitet, Sub-investigator); Elisabet Henley (Uppsala Universitet, Sub-investigator); Lena Kilander (Uppsala Universitet, Sub-investigator); Lena Propst (Uppsala Universitet, Sub-investigator); Malin Degerman Gunnarsson (Uppsala Universitet, Sub-investigator)

**Switzerland**

Reto Kressig, MD (University Hospital of Basel, PI); Manuel Haschke (University Hospital of Basel, Sub-investigator); Sibylle Bertoli (University Hospital of Basel, Sub-investigator); Viviane Steiner-Monard (University Hospital of Basel, Sub-investigator); Marc Baer (University Hospital of Basel, Sub-investigator); Francesca Lazzari (University Hospital of Basel, Sub-investigator); Simone Egli (University Hospital of Basel, Sub-investigator); Andreas Monsch (University Hospital of Basel, Sub-investigator); Christoph Stippich (University Hospital of Basel, Sub-investigator); Gabriel Gold, MD (Hôpitaux Universitaires de Genève, PI); Sven Haller (Hôpitaux Universitaires de Genève, Sub-investigator); Anne-Claude Juillerat Van der Linden (Hôpitaux Universitaires de Genève, Sub-investigator); Carine Bech (Hôpitaux Universitaires de Genève, Sub-investigator); Karl-Olof Lovblad (Hôpitaux Universitaires de Genève, Sub-investigator); Dina Zekry (Hôpitaux Universitaires de Genève, Sub-investigator); Catherine Prelaz (Hôpitaux Universitaires de Genève, Sub-investigator); Joelle Emmenegger (Hôpitaux Universitaires de Genève, Sub-investigator); Panteleimon Giannakopoulos, MD (Hôpitaux Universitaires de Genève, PI); Armin von Gunten (Hôpitaux Universitaires de Genève, Sub-investigator); Laura Rothuizen (Hôpitaux Universitaires de Genève, Sub-investigator); Monika Rybisar Van Dyke (Hôpitaux Universitaires de Genève, Sub-investigator); Beatrice Pellet (Hôpitaux Universitaires de Genève, Sub-investigator); Leilah Chouiter (Hôpitaux Universitaires de Genève, Sub-investigator); Louise Reichler (Hôpitaux Universitaires de Genève, Sub-investigator); Marzanna Wiechetek Ostos (Hôpitaux Universitaires de Genève, Sub-investigator); Melanie Favre (Hôpitaux Universitaires de Genève, Sub-investigator); Jerome Biollaz (Hôpitaux Universitaires de Genève, Sub-investigator); Reto Meuli (Hôpitaux Universitaires de Genève, Sub-investigator)

**United Kingdom**

Peter Bowie, MD (Grenoside Grange Hospital, PI); Janet Katherine Hutchison (Grenoside Grange Hospital, Sub-investigator); Barathy Kandhasamy (Grenoside Grange Hospital, Sub-investigator); Helen Linnington (Grenoside Grange Hospital, Sub-investigator); Antony Bayer, MBChB (Cardiff University, PI); Victoria Rabago (Cardiff University, Sub-investigator); Dennis Chan, MBChB, PhD (Royal Sussex County Hospital, PI); Rebecca Cooper (Royal Sussex County Hospital, Sub-investigator); Romi Saha (Royal Sussex County Hospital, Sub-investigator); Ruth Trimble (Royal Sussex County Hospital, Sub-investigator); Kuven Moodley (Royal Sussex County Hospital, Sub-investigator); Roger Alan Bullock, MBBS (Kingshill Research Centre, PI); Avinash Narayan (Kingshill Research Centre, Sub-investigator); Louise Patricia Keatings (Kingshill Research Centre, Sub-investigator); Fraser George Inglis, MBChB (Glasgow Memory Clinic Ltd, PI); Claire Sutherland (Glasgow Memory Clinic Ltd, Sub-investigator); Emma Chapman (Glasgow Memory Clinic Ltd, Sub-investigator); Erika Yvonne Letson (Glasgow Memory Clinic Ltd, Sub-investigator); Fiona Kinnon (Glasgow Memory Clinic Ltd, Sub-investigator); Jennifer Lynch (Glasgow Memory Clinic Ltd, Sub-investigator); Anne Turnbull (Glasgow Memory Clinic Ltd, Sub-investigator); Jennifer Morrice (Glasgow Memory Clinic Ltd, Sub-investigator); Sarah Smith (Glasgow Memory Clinic Ltd, Sub-investigator); Mark Christopher Dale, MBChB (Pollard Park Health Centre, PI); Arvind Gunput (Pollard Park Health Centre, Sub-investigator); Nigel Walker (Pollard Park Health Centre, Sub-investigator); Stuart H.R. Ratcliffe (Pollard Park Health Centre, Sub-investigator); Christopher McWilliam, MBChB (MAC UK Neuroscience Ltd., PI); Stuart Ratcliffe, MBChB (MAC UK Neuroscience Ltd., PI); Stephen Jackson, MBChB, MD (King’s College Hospital, PI); Omar Mukhtar (King’s College Hospital, Sub-investigator); Dag Aarsland (King’s College Hospital, Sub-investigator); Richard James Perry, MBBS, MD (Charing Cross Hospital, PI); Anil Ramlackhansingh (Charing Cross Hospital, Sub-investigator); Daniela Riano Baros (Charing Cross Hospital, Sub-investigator); Marios Politis (Charing Cross Hospital, Sub-investigator); Nicola Pavese (Charing Cross Hospital, Sub-investigator); Angus Michael David Kennedy (Charing Cross Hospital, Sub-investigator); Giorgio Gelosa (Charing Cross Hospital, Sub-investigator); Ann Donnelly (Charing Cross Hospital, Sub-investigator); Mateusz Pucek (Charing Cross Hospital, Sub-investigator); Melanie Wood (Charing Cross Hospital, Sub-investigator); Robert Barber, MBBS, MD (Newcastle University, PI); Noby Mathew George (Newcastle University, Sub-investigator); Sreenath Sangahalli (Newcastle University, Sub-investigator); Andrew Byrne (Newcastle University, Sub-investigator); Christopher Mark Davison (Newcastle University, Sub-investigator); Ian Grant McKeith (Newcastle University, Sub-investigator); Rosie Watson (Newcastle University, Sub-investigator); John O'Brien (Newcastle University, Sub-investigator); Paul Davies, MBChB, MD (Northampton General Hospital, PI); Subramanya Kumar (Northampton General Hospital, Sub-investigator); Paul Koranteng (Northampton General Hospital, Sub-investigator); Guruprasada Rao Nayani (Northampton General Hospital, Sub-investigator); Meriel Caroline Hollway (Northampton General Hospital, Sub-investigator); Sanjeev Shakhapur (Northampton General Hospital, Sub-investigator); John Starr, MD (Western General Hospital, PI);

**United States**

Eric Dale Kramer, MD (JEM Research Institute, PI); James Nathan Goldenberg (JEM Research Institute, Sub-investigator); Kyle Gordon (JEM Research Institute, Sub-investigator); Marco Barron (JEM Research Institute, Sub-investigator); Mark Allen Goldstein (JEM Research Institute, Sub-investigator); Jeanna Baker (JEM Research Institute, Sub-investigator); Rosemarie De Manna (JEM Research Institute, Sub-investigator); Shaffina Calideen (JEM Research Institute, Sub-investigator); Victor Biton, MD (Arkansas Research Program Clinical Trials, Inc., PI); Dusty L. Hollis-Holderfield (Arkansas Research Program Clinical Trials, Inc., Sub-investigator); Kristin Miller (Arkansas Research Program Clinical Trials, Inc., Sub-investigator); Teri Walters (Arkansas Research Program Clinical Trials, Inc., Sub-investigator); Gordon Leldon Gibson (Arkansas Research Program Clinical Trials, Inc., Sub-investigator); Jan Retherford Sullivan (Arkansas Research Program Clinical Trials, Inc., Sub-investigator); Kimberly Hagar (Arkansas Research Program Clinical Trials, Inc., Sub-investigator); Donna M. Hemphill (Arkansas Research Program Clinical Trials, Inc., Sub-investigator); Lauren L. Landers (Arkansas Research Program Clinical Trials, Inc., Sub-investigator); Jerome Goldstein, MD (San Francisco Clinical Research Center, PI); Bernadette A. Gabriel (San Francisco Clinical Research Center, Sub-investigator); Mary Gomes Tindle (San Francisco Clinical Research Center, Sub-investigator); Nadia M. Aissi (San Francisco Clinical Research Center, Sub-investigator); Rachael A. Henderson (San Francisco Clinical Research Center, Sub-investigator); Guy Robert Engelmann (San Francisco Clinical Research Center, Sub-investigator); Elliot Henderson (San Francisco Clinical Research Center, Sub-investigator); Callum James Rowe (San Francisco Clinical Research Center, Sub-investigator); Philip Yee (San Francisco Clinical Research Center, Sub-investigator); Tatiana Marcal (San Francisco Clinical Research Center, Sub-investigator); Pamela S. Gok (San Francisco Clinical Research Center, Sub-investigator); Rebecca E Brennan (San Francisco Clinical Research Center, Sub-investigator); Smita Dilip Kittur, MD (Neurological Care of Central New York, PI); Charity Cowley (Neurological Care of Central New York, Sub-investigator); Davina Robinson (Neurological Care of Central New York, Sub-investigator); Karen S. Pulvino (Neurological Care of Central New York, Sub-investigator); Leslie P. Golwitzer (Neurological Care of Central New York, Sub-investigator); Linda M. Schad (Neurological Care of Central New York, Sub-investigator); Mahender R. Goriganti (Neurological Care of Central New York, Sub-investigator); Mark Alphonse McConn (Neurological Care of Central New York, Sub-investigator); Robert Dracker (Neurological Care of Central New York, Sub-investigator); Amanda Sprague (Neurological Care of Central New York, Sub-investigator); Jennifer Chapman (Neurological Care of Central New York, Sub-investigator); Samantha Stratton (Neurological Care of Central New York, Sub-investigator); Liliana Montoya, MD (Neurostudies, Inc., PI); Lynn Marie Stengel (Neurostudies, Inc., Sub-investigator); Saeed Shazad (Neurostudies, Inc., Sub-investigator); Roy W. King (Neurostudies, Inc., Sub-investigator); George Li (Neurostudies, Inc., Sub-investigator); Robert B. Henegar (Neurostudies, Inc., Sub-investigator); Vicky Thursby (Neurostudies, Inc., Sub-investigator); Craig Thomas Curtis, MD (Compass Research, LLC, PI); Katy Smith (Compass Research, LLC, Sub-investigator); Marianne Ripley (Compass Research, LLC, Sub-investigator); Sigrid (Sandi) McMahon (Compass Research, LLC, Sub-investigator); Timothy William Schubert (Compass Research, LLC, Sub-investigator); Eva-Maria Heurich (Compass Research, LLC, Sub-investigator); James Samir McDonough (Compass Research, LLC, Sub-investigator); Kelly Nanette Taylor (Compass Research, LLC, Sub-investigator); Michael Joseph Creamer (Compass Research, LLC, Sub-investigator); William Kelly Bowman (Compass Research, LLC, Sub-investigator); Victoria L. Marsh (Compass Research, LLC, Sub-investigator); Jay Ellis, DO (Neuroscience Research of the Berkshires, PI); Barbara M. Bashara (Neuroscience Research of the Berkshires, Sub-investigator); Luanne T. Bednar (Neuroscience Research of the Berkshires, Sub-investigator); Bonnie J. Hess (Neuroscience Research of the Berkshires, Sub-investigator); Elaine Kalinowski (Neuroscience Research of the Berkshires, Sub-investigator); Susan McCauley (Neuroscience Research of the Berkshires, Sub-investigator); Kathleen K. Noto (Neuroscience Research of the Berkshires, Sub-investigator); Arlene Shreefter (Neuroscience Research of the Berkshires, Sub-investigator); Julie M. Steinman (Neuroscience Research of the Berkshires, Sub-investigator); Kimberly A. Weslowski (Neuroscience Research of the Berkshires, Sub-investigator); Tammy K. Willis Kisselbrock (Neuroscience Research of the Berkshires, Sub-investigator); Corey Anderson, MD (Dedicated Clinical Research, PI); Cedar Bennett (Dedicated Clinical Research, Sub-investigator); Clarence Jason Vastine (Dedicated Clinical Research, Sub-investigator); Dane A. Higgins (Dedicated Clinical Research, Sub-investigator); Daniel Torzala (Dedicated Clinical Research, Sub-investigator); Emese C. Torok (Dedicated Clinical Research, Sub-investigator); Jillian M. Hurlbut-Styke (Dedicated Clinical Research, Sub-investigator); Joseph B. Earl (Dedicated Clinical Research, Sub-investigator); Katie Mungari (Dedicated Clinical Research, Sub-investigator); Katrina Nelson (Dedicated Clinical Research, Sub-investigator); Kelli A. Bingham (Dedicated Clinical Research, Sub-investigator); Shanna Nixon (Dedicated Clinical Research, Sub-investigator); Susan A. Scott (Dedicated Clinical Research, Sub-investigator); Ann Marie Sun (Dedicated Clinical Research, Sub-investigator); David Engstrom (Dedicated Clinical Research, Sub-investigator); Mark R. Lonquist (Dedicated Clinical Research, Sub-investigator); Troy G. Anderson (Dedicated Clinical Research, Sub-investigator); Hazel Lam (Dedicated Clinical Research, Sub-investigator); Laurie Becerra (Dedicated Clinical Research, Sub-investigator); Rhiannon Strasser (Dedicated Clinical Research, Sub-investigator); Virginia Ramirez (Dedicated Clinical Research, Sub-investigator); Carly G. Carhill (Dedicated Clinical Research, Sub-investigator); Judith C. Engelman (Dedicated Clinical Research, Sub-investigator); James Garrison, MD (Innovative Clinical Trials, PI); Amy Damon (Innovative Clinical Trials, Sub-investigator); Elizabeth A. Hendrix (Innovative Clinical Trials, Sub-investigator); Rhonda Yeatts (Innovative Clinical Trials, Sub-investigator); Gregory Duncan McCarroll (Innovative Clinical Trials, Sub-investigator); Stephen Francis Hoffman (Innovative Clinical Trials, Sub-investigator); Kerry L. deJesus (Innovative Clinical Trials, Sub-investigator); Jeffrey Burns, MD, MS (University of Kansas Medical Center, PI); Heather S. Anderson (University of Kansas Medical Center, Sub-investigator); Anne K. Arthur (University of Kansas Medical Center, Sub-investigator); Jami Lynn Goodwin (University of Kansas Medical Center, Sub-investigator); Pat Laubinger (University of Kansas Medical Center, Sub-investigator); Cherie Marie Parker (University of Kansas Medical Center, Sub-investigator); Russell H. Swerdlow (University of Kansas Medical Center, Sub-investigator); Phyllis A. Switzer (University of Kansas Medical Center, Sub-investigator); John Ervin, MD (The Center for Pharmaceutical Research, PC, PI); Amy Gorsuch (The Center for Pharmaceutical Research, PC, Sub-investigator); Jan Bedord (The Center for Pharmaceutical Research, PC, Sub-investigator); Kelly Moen (The Center for Pharmaceutical Research, PC, Sub-investigator); Laurenze L. Ducatte (The Center for Pharmaceutical Research, PC, Sub-investigator); Leslyn Q. Brouillette (The Center for Pharmaceutical Research, PC, Sub-investigator); Melissa Sandefur-Lander (The Center for Pharmaceutical Research, PC, Sub-investigator); Pamela D. Dubin (The Center for Pharmaceutical Research, PC, Sub-investigator); Patricia Ann Wyatt (The Center for Pharmaceutical Research, PC, Sub-investigator); Sandra C. Edwards (The Center for Pharmaceutical Research, PC, Sub-investigator); Sarah E. Jokerst (The Center for Pharmaceutical Research, PC, Sub-investigator); Shannon Hunt (The Center for Pharmaceutical Research, PC, Sub-investigator); Susan C. Pomeroy (The Center for Pharmaceutical Research, PC, Sub-investigator); Asim Ulusarac (The Center for Pharmaceutical Research, PC, Sub-investigator); Matthew D Tyson (The Center for Pharmaceutical Research, PC, Sub-investigator); Linda C. Irwin (The Center for Pharmaceutical Research, PC, Sub-investigator); Sharon E. Ervin (The Center for Pharmaceutical Research, PC, Sub-investigator); Jennifer N. Lindgren (The Center for Pharmaceutical Research, PC, Sub-investigator); Dario Zagar, MD (Associated Neurologists of Southern CT, PI); Angelo Termine (Associated Neurologists of Southern CT, Sub-investigator); Bozena Czapka (Associated Neurologists of Southern CT, Sub-investigator); Diana E. Miner (Associated Neurologists of Southern CT, Sub-investigator); Grace Castaneda (Associated Neurologists of Southern CT, Sub-investigator); Karen Brown (Associated Neurologists of Southern CT, Sub-investigator); Nick Adams (Associated Neurologists of Southern CT, Sub-investigator); Amy B. Palmer (Associated Neurologists of Southern CT, Sub-investigator); Christine McCarthy (Associated Neurologists of Southern CT, Sub-investigator); Jeffrey L. Gross (Associated Neurologists of Southern CT, Sub-investigator); Kenneth Colman Siegel (Associated Neurologists of Southern CT, Sub-investigator); Srinath Kadimi (Associated Neurologists of Southern CT, Sub-investigator); Thomas B. Toothaker (Associated Neurologists of Southern CT, Sub-investigator); John Stoukides, MD, DSc, RPh (Rhode Island Mood & Memory Research Institute, PI); Holly Adams (Rhode Island Mood & Memory Research Institute, Sub-investigator); Anne Louise Cerullo (Rhode Island Mood & Memory Research Institute, Sub-investigator); Donna Cimini (Rhode Island Mood & Memory Research Institute, Sub-investigator); Jennifer M. Ritzau (Rhode Island Mood & Memory Research Institute, Sub-investigator); Cheryl Stoukides (Rhode Island Mood & Memory Research Institute, Sub-investigator); Gilbert M. Teixeira (Rhode Island Mood & Memory Research Institute, Sub-investigator); Marvin Kalafer, MD, FACOG (The Clinical Trial Center, LLC, PI); Alketa Dobi (The Clinical Trial Center, LLC, Sub-investigator); Anita Cummins (The Clinical Trial Center, LLC, Sub-investigator); Anna R. Wilkening (The Clinical Trial Center, LLC, Sub-investigator); Mary Charlotte Lyman (The Clinical Trial Center, LLC, Sub-investigator); Peter Pressman (The Clinical Trial Center, LLC, Sub-investigator); Gene R. Corbman (The Clinical Trial Center, LLC, Sub-investigator); Steven Edward Arnold (The Clinical Trial Center, LLC, Sub-investigator); Jacob Dubroff (The Clinical Trial Center, LLC, Sub-investigator); Lori Martin (The Clinical Trial Center, LLC, Sub-investigator); David C. Weisman (The Clinical Trial Center, LLC, Sub-investigator); Scott Losk, PhD (Summit Research Network (Oregon) Inc., PI); Ramy H. Adamowski (Summit Research Network (Oregon) Inc., Sub-investigator); James Harold Bergthold (Summit Research Network (Oregon) Inc., Sub-investigator); MaryAnn Conrad (Summit Research Network (Oregon) Inc., Sub-investigator); Carrie Crider (Summit Research Network (Oregon) Inc., Sub-investigator); Linda K. Marambe (Summit Research Network (Oregon) Inc., Sub-investigator); Lawrence H. Moore (Summit Research Network (Oregon) Inc., Sub-investigator); Marcella Ottum (Summit Research Network (Oregon) Inc., Sub-investigator); Ward Tolbert Smith (Summit Research Network (Oregon) Inc., Sub-investigator); Karen R. Wilson (Summit Research Network (Oregon) Inc., Sub-investigator); Alan S. Yeo (Summit Research Network (Oregon) Inc., Sub-investigator); Balebail Ashok Raj, MD (University of South Florida, PI); Jill Ardila (University of South Florida, Sub-investigator); Kristin O'Dell Fargher (University of South Florida, Sub-investigator); Barbara M. Luhn (University of South Florida, Sub-investigator); Beth A. Major (University of South Florida, Sub-investigator); Laura Murray (University of South Florida, Sub-investigator); Amanda Grant Smith (University of South Florida, Sub-investigator); Nancy Teten (University of South Florida, Sub-investigator); Glenn Whelan (University of South Florida, Sub-investigator); Concetta Maria Forchetti, MD, PhD (Alexian Brothers Neurosciences Institute, PI); Brian Leahy (Alexian Brothers Neurosciences Institute, Sub-investigator); Christina D. Kay (Alexian Brothers Neurosciences Institute, Sub-investigator); Donnell M. Carmichael (Alexian Brothers Neurosciences Institute, Sub-investigator); Robert Frech (Alexian Brothers Neurosciences Institute, Sub-investigator); Jeanine M. Pilat (Alexian Brothers Neurosciences Institute, Sub-investigator); Julia A. Valerio (Alexian Brothers Neurosciences Institute, Sub-investigator); Lynette K. Chism (Alexian Brothers Neurosciences Institute, Sub-investigator); Michelle L. Sanfilippo (Alexian Brothers Neurosciences Institute, Sub-investigator); Stephanie Blair (Alexian Brothers Neurosciences Institute, Sub-investigator); Maria E. Kyanka (Alexian Brothers Neurosciences Institute, Sub-investigator); Michael Rossen, MD, PhD (Springfield Neurology Associates, PI); Adam Lichtenstein (Springfield Neurology Associates, Sub-investigator); Carin A. Nelson (Springfield Neurology Associates, Sub-investigator); Taryn Lynn Lapponese (Springfield Neurology Associates, Sub-investigator); Emilio M. Melchionna (Springfield Neurology Associates, Sub-investigator); Marcello Fernando Di Carli (Springfield Neurology Associates, Sub-investigator); Michael R. Sorrell (Springfield Neurology Associates, Sub-investigator); Keith A. Johnson (Springfield Neurology Associates, Sub-investigator); Robert Stern, PhD (Boston University, ADCRP, PI); Carol J. Rossi (Boston University, ADCRP, Sub-investigator); Jane G. Mwicigi (Boston University, ADCRP, Sub-investigator); Marcello Fernando Di Carli (Boston University, ADCRP, Sub-investigator); Wendy W. Qiu (Boston University, ADCRP, Sub-investigator); Eric G. Steinberg (Boston University, ADCRP, Sub-investigator); Keith A. Johnson (Boston University, ADCRP, Sub-investigator); Patricia L. Johnson (Boston University, ADCRP, Sub-investigator); Sumati V. Raghavan (Boston University, ADCRP, Sub-investigator); Theresa A. McGowan (Boston University, ADCRP, Sub-investigator); Gary Tunell, MD, PhD (Texas Neurology PA, PI); Johnathan Brent Spears (Texas Neurology PA, Sub-investigator); Margaret E. Hastings (Texas Neurology PA, Sub-investigator); Quanetta L. Davis (Texas Neurology PA, Sub-investigator); Charles Fish Greenfield (Texas Neurology PA, Sub-investigator); Greg S. Wood (Texas Neurology PA, Sub-investigator); Joanne Crosley (Texas Neurology PA, Sub-investigator); LuAnn S. Tunell (Texas Neurology PA, Sub-investigator); Christi L. Kelly (Texas Neurology PA, Sub-investigator); Sheilah Harned-Storch (Texas Neurology PA, Sub-investigator); Debra Kulchycki (Palm Beach Neurological Center, Sub-investigator); Justin M. Pratt (Palm Beach Neurological Center, Sub-investigator); Karen L. McCallister (Palm Beach Neurological Center, Sub-investigator); Laura R. Punckowski (Palm Beach Neurological Center, Sub-investigator); Lisa S. Montgomery (Palm Beach Neurological Center, Sub-investigator); Meredith D. Iwasz (Palm Beach Neurological Center, Sub-investigator); Michelle Bender (Palm Beach Neurological Center, Sub-investigator); Rhonda L. Skiles (Palm Beach Neurological Center, Sub-investigator); Vivian Carta-Sanchez (Palm Beach Neurological Center, Sub-investigator); Jeffrey Barnett Brown (Palm Beach Neurological Center, Sub-investigator); Carol Tuchman (Palm Beach Neurological Center, Sub-investigator); MaryAnn Rahe (Palm Beach Neurological Center, Sub-investigator); Thomas Andrew Ala, MD (Southern Illinois University School of Medicine, PI); Barbara Ann Cray Lokaitis (Southern Illinois University School of Medicine, Sub-investigator); Charlene J. Young (Southern Illinois University School of Medicine, Sub-investigator); Ronald Zec (Southern Illinois University School of Medicine, Sub-investigator); Ann Popovich (Southern Illinois University School of Medicine, Sub-investigator); Stephanie Kohlrus (Southern Illinois University School of Medicine, Sub-investigator); Amy L. Richey (Southern Illinois University School of Medicine, Sub-investigator); Sally A.H. Fritz (Southern Illinois University School of Medicine, Sub-investigator); Joel Ross, MD, FACP (Memory Enhancement Center of America, Inc., PI); Barry B. Leskowitz (Memory Enhancement Center of America, Inc., Sub-investigator); Patricia A. Arnold (Memory Enhancement Center of America, Inc., Sub-investigator); Victoria Dioguardi (Memory Enhancement Center of America, Inc., Sub-investigator); Lois M. Donovan (Memory Enhancement Center of America, Inc., Sub-investigator); Kaycee C. Doyle (Memory Enhancement Center of America, Inc., Sub-investigator); Jose F. Gomez (Memory Enhancement Center of America, Inc., Sub-investigator); Tatiana Kundla (Memory Enhancement Center of America, Inc., Sub-investigator); Mark Ornstein (Memory Enhancement Center of America, Inc., Sub-investigator); Debra Ross (Memory Enhancement Center of America, Inc., Sub-investigator); Kelly Wilder-Willis (Memory Enhancement Center of America, Inc., Sub-investigator); Marshall Nash, MD, FAHA, CPI (Dekalb Neurology Associates, LLC/NeuroStudies.net, LLC/Neuroscience Research Institute, LLC at Gwinnett Medical Center, PI); Celina Hazelrig (Dekalb Neurology Associates, LLC/NeuroStudies.net, LLC/Neuroscience Research Institute, LLC at Gwinnett Medical Center, Sub-investigator); Glenda Yarbrough (Dekalb Neurology Associates, LLC/NeuroStudies.net, LLC/Neuroscience Research Institute, LLC at Gwinnett Medical Center, Sub-investigator); Lavanya Vedanarayanan (Dekalb Neurology Associates, LLC/NeuroStudies.net, LLC/Neuroscience Research Institute, LLC at Gwinnett Medical Center, Sub-investigator); Robin R. Jackson (NeuroStudies.net, LLC, Sub-investigator); Scott H. Chandler (Dekalb Neurology Associates, LLC/NeuroStudies.net, LLC/Neuroscience Research Institute, LLC at Gwinnett Medical Center, Sub-investigator); David Albert Olson (Dekalb Neurology Associates, LLC/NeuroStudies.net, LLC/Neuroscience Research Institute, LLC at Gwinnett Medical Center, Sub-investigator); Joshua Allen Turknett (Dekalb Neurology Associates, LLC/NeuroStudies.net, LLC/Neuroscience Research Institute, LLC at Gwinnett Medical Center, Sub-investigator); Christina Flemister (Dekalb Neurology Associates, LLC/NeuroStudies.net, LLC, Sub-investigator); Sherry L. Myers (Dekalb Neurology Associates, LLC/NeuroStudies.net, LLC/Neuroscience Research Institute, LLC at Gwinnett Medical Center, Sub-investigator); Karen N. Young (NeuroStudies.net, LLC/Neuroscience Research Institute, LLC at Gwinnett Medical Center, Sub-investigator); Leah Previto Craft (Dekalb Neurology Associates, LLC/NeuroStudies.net, LLC/Neuroscience Research Institute, LLC at Gwinnett Medical Center, Sub-investigator); Myrna L. Whiting (Dekalb Neurology Associates, LLC/NeuroStudies.net, LLC, Sub-investigator); Shanna Bruflodt (Dekalb Neurology Associates, LLC, Sub-investigator); Arthur D. Schiff (NeuroStudies.net, LLC, Sub-investigator); Anthony M. Cutsuries (Neuroscience Research Institute, LLC at Gwinnett Medical Center, Sub-investigator); Spencer I. Rozin (Neuroscience Research Institute, LLC at Gwinnett Medical Center, Sub-investigator); Karen N. Young (NeuroStudies.net, LLC/Neuroscience Research Institute, LLC at Gwinnett Medical Center, Sub-investigator); Bart Sloan, MD (Research Center for Clinical Studies, Inc., PI); Danilo de la Pena (Research Center for Clinical Studies, Inc., Sub-investigator); Jesselle J. de la Pena (Research Center for Clinical Studies, Inc., Sub-investigator); Mihirkumar H. Patel (Research Center for Clinical Studies, Inc., Sub-investigator); Kinjalben Y. Patel (Research Center for Clinical Studies, Inc., Sub-investigator); Donna Phanumas (Research Center for Clinical Studies, Inc., Sub-investigator); Mahesh B. Rai (Research Center for Clinical Studies, Inc., Sub-investigator); Sabin Kumar Shrestha (Research Center for Clinical Studies, Inc., Sub-investigator); David Weisman, MD (Abington Neurological Associates, PI); Allyson M. Blue-Schaller (Abington Neurological Associates, Sub-investigator); Vicki Boruta (Abington Neurological Associates, Sub-investigator); Brad Klein (Abington Neurological Associates, Sub-investigator); B. Franklin Diamond (Abington Neurological Associates, Sub-investigator); Steven Edward Arnold (Abington Neurological Associates, Sub-investigator); Keith A. Johnson (Abington Neurological Associates, Sub-investigator); Andrea L. Years (Abington Neurological Associates, Sub-investigator); Jennifer McGowan (Abington Neurological Associates, Sub-investigator); Andrea J. Casher (Abington Neurological Associates, Sub-investigator); Cynthia D. Oliva (Abington Neurological Associates, Sub-investigator); Michael Mega, MD, PhD (Providence St. Vincent Medical Center, PI); Barbara Siaroff (Providence St. Vincent Medical Center, Sub-investigator); Lynette Rogers (Providence St. Vincent Medical Center, Sub-investigator); Mary Huddleston (Providence St. Vincent Medical Center, Sub-investigator); Stanley Lawrence Cohan (Providence St. Vincent Medical Center, Sub-investigator); Kyle E. Smoot (Providence St. Vincent Medical Center, Sub-investigator); Caitlin B. Curtis (Providence St. Vincent Medical Center, Sub-investigator); Paul Solomon, PhD (The Memory Clinic, PI); Rita L. Burgher (The Memory Clinic, Sub-investigator); Megan Casey (The Memory Clinic, Sub-investigator); Lisa Karen Catapano-Friedman (The Memory Clinic, Sub-investigator); Michael Dufort (The Memory Clinic, Sub-investigator); Paula D. Levin (The Memory Clinic, Sub-investigator); Diana Michalczuk (The Memory Clinic, Sub-investigator); Cynthia A. Murphy (The Memory Clinic, Sub-investigator); Roger Paro (The Memory Clinic, Sub-investigator); Martha Stitelman (The Memory Clinic, Sub-investigator); Mary K. Horton (The Memory Clinic, Sub-investigator); Mary Pat Mazzola (The Memory Clinic, Sub-investigator); Joseph Hendizadeh Rodd, MD (AVI Institute, Inc, PI); Helen Chen (AVI Institute, Inc, Sub-investigator); Phyllis Huang (AVI Institute, Inc, Sub-investigator); Maelaine Rodero (AVI Institute, Inc, Sub-investigator); Carlos E. Valdes (AVI Institute, Inc, Sub-investigator); Elizabeth Zarate-Rowell (AVI Institute, Inc, Sub-investigator); Omid Omidvar, MD (Collaborative Neuroscience Network, Inc., PI); Candice M. Britton (Collaborative Neuroscience Network, Inc., Sub-investigator); Christine Tanimura (Collaborative Neuroscience Network, Inc., Sub-investigator); Cynthia Keenan (Collaborative Neuroscience Network, Inc., Sub-investigator); Donna Bui (Collaborative Neuroscience Network, Inc., Sub-investigator); Pennie H. Lam (Collaborative Neuroscience Network, Inc., Sub-investigator); Alan Howard Cohen (Collaborative Neuroscience Network, Inc., Sub-investigator); Armen Krikor Goenjian (Collaborative Neuroscience Network, Inc., Sub-investigator); Christopher Webb (Collaborative Neuroscience Network, Inc., Sub-investigator); Jonathan Leonard Brand (Collaborative Neuroscience Network, Inc., Sub-investigator); Karen L. Spoor (Collaborative Neuroscience Network, Inc., Sub-investigator); Kim Lorine (Collaborative Neuroscience Network, Inc., Sub-investigator); Melissa P. Huy (Collaborative Neuroscience Network, Inc., Sub-investigator); Nima Ramezan-Arab (Collaborative Neuroscience Network, Inc., Sub-investigator); Nirav S. Patel (Collaborative Neuroscience Network, Inc., Sub-investigator); Sheida A. Kashani (Collaborative Neuroscience Network, Inc., Sub-investigator); Steven H. Reynolds (Collaborative Neuroscience Network, Inc., Sub-investigator); Thanh Ho (Collaborative Neuroscience Network, Inc., Sub-investigator); Gregory Ralph Bonomo (Collaborative Neuroscience Network, Inc., Sub-investigator); Jill E. Schmidt (Collaborative Neuroscience Network, Inc., Sub-investigator); Anne M. Cabral (Collaborative Neuroscience Network, Inc., Sub-investigator); Suzanne Gazda, MD, PharmD (Integra Clinical Research, LLC, PI); Cheryl A. Collins (Integra Clinical Research, LLC, Sub-investigator); Delia Munoz (Integra Clinical Research, LLC, Sub-investigator); Mireya R Garza (Integra Clinical Research, LLC, Sub-investigator); Gilbert Martinez (Integra Clinical Research, LLC, Sub-investigator); Ellen Swann Van Delden (Integra Clinical Research, LLC, Sub-investigator); Richard Braden Neiman (Integra Clinical Research, LLC, Sub-investigator); Romana Sibyl Kleinguenther (Integra Clinical Research, LLC, Sub-investigator); Carrie Lunceford (Integra Clinical Research, LLC, Sub-investigator); Alisa Zinsmeyer Young (Integra Clinical Research, LLC, Sub-investigator); Johanna N. Villasenor (Integra Clinical Research, LLC, Sub-investigator); Olga P. Dib (Integra Clinical Research, LLC, Sub-investigator); Joshua Shua-Haim, MD, FACP (Alzheimer’s Research Corporation, PI); Mira Ahuja (Alzheimer’s Research Corporation, Sub-investigator); Zhanetta Cheshun (Alzheimer’s Research Corporation, Sub-investigator); Suhas R. Patel (Alzheimer’s Research Corporation, Sub-investigator); Pamela Poole (Alzheimer’s Research Corporation, Sub-investigator); Vered Shua-Haim (Alzheimer’s Research Corporation, Sub-investigator); Juanita M. Smith (Alzheimer’s Research Corporation, Sub-investigator); Mark Brody, MD (Brain Matters Research, PI); Amanda Hackebeil (Brain Matters Research, Sub-investigator); Ana Scolari-Fuquay (Brain Matters Research, Sub-investigator); Yara Ramirez-King (Brain Matters Research, Sub-investigator); David Watson (Brain Matters Research, Sub-investigator); Joseph M. Mauceri (Brain Matters Research, Sub-investigator); Lisa Wahlestedt (Brain Matters Research, Sub-investigator); Gila Barbati (Brain Matters Research, Sub-investigator); Jessica Espinoza (Brain Matters Research, Sub-investigator); Jack Klapper, MD (Mile High Research Center, PI); Amy Greaves (Mile High Research Center, Sub-investigator); Hannah Rolph (Mile High Research Center, Sub-investigator); Julia Waldman (Mile High Research Center, Sub-investigator); Kent A. Wilson (Mile High Research Center, Sub-investigator); Francis Kinney, MD, PhD (Callahan Eye Foundation Hospital, PI); Hollis E. Reeves (Callahan Eye Foundation Hospital, Sub-investigator); Nancy Kirby (Callahan Eye Foundation Hospital, Sub-investigator); Roberta Sokol May (Callahan Eye Foundation Hospital, Sub-investigator); Samantha White (Callahan Eye Foundation Hospital, Sub-investigator); Sherer Boswell Thomson (Callahan Eye Foundation Hospital, Sub-investigator); Zahra A. Rahman (Callahan Eye Foundation Hospital, Sub-investigator); Lelland C. Tolbert (Callahan Eye Foundation Hospital, Sub-investigator); Rebecca Quinn (Callahan Eye Foundation Hospital, Sub-investigator); Richard Shelton (Callahan Eye Foundation Hospital, Sub-investigator); Dan Dahl (Callahan Eye Foundation Hospital, Sub-investigator); Robert Savage (Callahan Eye Foundation Hospital, Sub-investigator); Madison Bates Redwine (Callahan Eye Foundation Hospital, Sub-investigator); Terri Steele (Callahan Eye Foundation Hospital, Sub-investigator); Alan Zacharias, MD (Associated Neurologists, PC, PI); Beverly C. Solis (Associated Neurologists, PC, Sub-investigator); Janice A. Miller (Associated Neurologists, PC, Sub-investigator); Margaret E. Sharp (Associated Neurologists, PC, Sub-investigator); Paul Kenneth Brownstone (Associated Neurologists, PC, Sub-investigator); Anita Teague (Associated Neurologists, PC, Sub-investigator); Deb Shioshita (Associated Neurologists, PC, Sub-investigator); Steven Trent DeKosky, MD (University of Virginia Health System, PI); Anita Thompson-Heisterman (University of Virginia Health System, Sub-investigator); Colleen Napier Webber (University of Virginia Health System, Sub-investigator); Daniel Claassen (University of Virginia Health System, Sub-investigator); Erin Foff (University of Virginia Health System, Sub-investigator); Julie A. Matsumoto (University of Virginia Health System, Sub-investigator); Karen D. Pollard (University of Virginia Health System, Sub-investigator); Paula Damgaard (University of Virginia Health System, Sub-investigator); Scott Sperling (University of Virginia Health System, Sub-investigator); Carol A. Manning (University of Virginia Health System, Sub-investigator); Nancy McLinskey (University of Virginia Health System, Sub-investigator); Robert Stephen Davis (University of Virginia Health System, Sub-investigator); Adam McDermott (University of Virginia Health System, Sub-investigator); Guillermo Solorzano (University of Virginia Health System, Sub-investigator); John Christopher (University of Virginia Health System, Sub-investigator); Stephen Zieman Jr. (University of Virginia Health System, Sub-investigator); Rosetta Opie (University of Virginia Health System, Sub-investigator); David Stephen Geldmacher, MD (University of Virginia Health System, PI); Patricia Naslund, MD (Raleigh Neurology Associates, PI); Holly Hall Starnes (Raleigh Neurology Associates, Sub-investigator); Rhonda Winstead Gabr (Raleigh Neurology Associates, Sub-investigator); Theresa B. Keiser (Raleigh Neurology Associates, Sub-investigator); David Aslan Konanc (Raleigh Neurology Associates, Sub-investigator); Keith Lowell Hull Jr. (Raleigh Neurology Associates, Sub-investigator); Kenneth Michael Carnes (Raleigh Neurology Associates, Sub-investigator); Michael Higgins Bowman (Raleigh Neurology Associates, Sub-investigator); Susan Annette Glenn (Raleigh Neurology Associates, Sub-investigator); William Gregory Ferrell (Raleigh Neurology Associates, Sub-investigator); Susan Elizabeth Broadway (Raleigh Neurology Associates, Sub-investigator); Christopher van Dyck, MD (Yale University School of Medicine, PI); Allison F. Wagner (Yale University School of Medicine, Sub-investigator); David W. Cheng (Yale University School of Medicine, Sub-investigator); Diana L. Ricitelli (Yale University School of Medicine, Sub-investigator); Garrett S. Bowen (Yale University School of Medicine, Sub-investigator); Haakon Nygaard (Yale University School of Medicine, Sub-investigator); Ilse Wiechers (Yale University School of Medicine, Sub-investigator); Katherine L. Paturzo (Yale University School of Medicine, Sub-investigator); Marc P. Nespoli (Yale University School of Medicine, Sub-investigator); Michelle D. Vinci (Yale University School of Medicine, Sub-investigator); Moshe Hasbani (Yale University School of Medicine, Sub-investigator); Nicole M. Barcelos (Yale University School of Medicine, Sub-investigator); Norman S. Werdiger (Yale University School of Medicine, Sub-investigator); Pilar Laborde-Lahoz (Yale University School of Medicine, Sub-investigator); Sarah C. Taylor (Yale University School of Medicine, Sub-investigator); Sarah Jane C. De Asis (Yale University School of Medicine, Sub-investigator); Satish Vallabhanei (Yale University School of Medicine, Sub-investigator); Shriti Patel (Yale University School of Medicine, Sub-investigator); Sigrid D. Wiemers (Yale University School of Medicine, Sub-investigator); Susan P. Good (Yale University School of Medicine, Sub-investigator); Teide Brisibe (Yale University School of Medicine, Sub-investigator); Juan Carlos Cleves-Bayon (Yale University School of Medicine, Sub-investigator); Martha G. MacAvoy (Yale University School of Medicine, Sub-investigator); Samantha K. Henry (Yale University School of Medicine, Sub-investigator); Kashinath Gangadhara Yadalam, MBBS (Lake Charles Clinical Trials, PI); Allyson D. Bennett (Lake Charles Clinical Trials, Sub-investigator); Charles Murphy (Lake Charles Clinical Trials, Sub-investigator); Colleen Smith (Lake Charles Clinical Trials, Sub-investigator); Jamie R. Saucier (Lake Charles Clinical Trials, Sub-investigator); Lenie T. Doucet (Lake Charles Clinical Trials, Sub-investigator); Mary Long (Lake Charles Clinical Trials, Sub-investigator); Sean F. Gardiner (Lake Charles Clinical Trials, Sub-investigator); Sharon Garrett (Lake Charles Clinical Trials, Sub-investigator); Therese A. Novak (Lake Charles Clinical Trials, Sub-investigator); Charles Boyd Woodard (Lake Charles Clinical Trials, Sub-investigator); Connie Campbell (Lake Charles Clinical Trials, Sub-investigator); Kami L. Fruge (Lake Charles Clinical Trials, Sub-investigator); Leenora Lohr (Lake Charles Clinical Trials, Sub-investigator); Melissa Thibodeaux (Lake Charles Clinical Trials, Sub-investigator); Danette Beard (Lake Charles Clinical Trials, Sub-investigator); Patty M. Victory (Lake Charles Clinical Trials, Sub-investigator); Andrea Bozoki, MD (Michigan State University, PI); Stuart L. Doneson (Michigan State University, Sub-investigator); Igor O. Korolev (Michigan State University, Sub-investigator); John Lester Goudreau (Michigan State University, Sub-investigator); Kevin Thomas Foley (Michigan State University, Sub-investigator); Tashia McGhee Helmy (Michigan State University, Sub-investigator); Theresa Doerr (Michigan State University, Sub-investigator); Doozie M.L. Russell (Michigan State University, Sub-investigator); Krista R. Leiter (Michigan State University, Sub-investigator); Nancy Barbas, MSW, MD (University of Michigan Health System, PI); Bruno Giordani (University of Michigan Health System, Sub-investigator); Judith L. Heidebrink (University of Michigan Health System, Sub-investigator); Mike Kilbourn (University of Michigan Health System, Sub-investigator); Joanne L. Lord (University of Michigan Health System, Sub-investigator); Tara L. Riddle (University of Michigan Health System, Sub-investigator); Gaurange Shah (University of Michigan Health System, Sub-investigator); Fu Lye Woon (University of Michigan Health System, Sub-investigator); Barry Rovner, MD (Thomas Jefferson University, PI); Janice M. Carsello (Thomas Jefferson University, Sub-investigator); Daniel Kremens (Thomas Jefferson University, Sub-investigator); Eileen K Maloney (Thomas Jefferson University, Sub-investigator); Melissa McElwaine (Thomas Jefferson University, Sub-investigator); Rania Sadaka (Thomas Jefferson University, Sub-investigator); Linda A. Sailor (Thomas Jefferson University, Sub-investigator); Amanda J. Shaffer (Thomas Jefferson University, Sub-investigator); Christopher Thomas Skidmore (Thomas Jefferson University, Sub-investigator); Ryan T. Tisera (Thomas Jefferson University, Sub-investigator); Jacobo Mintzer, MD, MBA (Medical University of South Carolina, PI); Amanda Watts (Medical University of South Carolina, Sub-investigator); Arthur L. Williams (Medical University of South Carolina, Sub-investigator); Bonnie K. Muntz-Pope (Medical University of South Carolina, Sub-investigator); Elizabeth Hamilton (Medical University of South Carolina, Sub-investigator); Erin E. Jarrett (Medical University of South Carolina, Sub-investigator); Jan M. Watts (Medical University of South Carolina, Sub-investigator); Jessica Broadway (Medical University of South Carolina, Sub-investigator); Marilyn Kay Stuckey (Medical University of South Carolina, Sub-investigator); Mary Elizabeth Safrit (Medical University of South Carolina, Sub-investigator); Thad Modlin (Medical University of South Carolina, Sub-investigator); William Pursley (Medical University of South Carolina, Sub-investigator); David Louis Bachman (Medical University of South Carolina, Sub-investigator); Kenneth McRae Spicer (Medical University of South Carolina, Sub-investigator); Mary Craig (Medical University of South Carolina, Sub-investigator); Olga Brawman-Mintzer (Medical University of South Carolina, Sub-investigator); Richard Snider (Medical University of South Carolina, Sub-investigator); Crystal Flynn Longmire (Medical University of South Carolina, Sub-investigator); Jessica Holycross (Medical University of South Carolina, Sub-investigator); Robin Cherry (Medical University of South Carolina, Sub-investigator); Sara Speakman (Medical University of South Carolina, Sub-investigator); Lisa M. Dempsey (Medical University of South Carolina, Sub-investigator); Stephanie N. Ennis (Medical University of South Carolina, Sub-investigator); Vibhor Krishna (Medical University of South Carolina, Sub-investigator); Steven Arnold, MD (University of Pennsylvania School of Medicine, PI); Jessica Nunez (University of Pennsylvania School of Medicine, Sub-investigator); Roy Hamilton (University of Pennsylvania School of Medicine, Sub-investigator); Steven F. Huege (University of Pennsylvania School of Medicine, Sub-investigator); Hannah McCoubrey (University of Pennsylvania School of Medicine, Sub-investigator); Peter Janis (University of Pennsylvania School of Medicine, Sub-investigator); Rich Freifelder (University of Pennsylvania School of Medicine, Sub-investigator); David A. Wolk (University of Pennsylvania School of Medicine, Sub-investigator); Jason H.T. Karlawish (University of Pennsylvania School of Medicine, Sub-investigator); Jacob Dubroff (University of Pennsylvania School of Medicine, Sub-investigator); Paige H. Brookstein (University of Pennsylvania School of Medicine, Sub-investigator); Douglas Scharre, MD (The Ohio State University, PI); Allison Seward (The Ohio State University, Sub-investigator); Barbara Eason Himes (The Ohio State University, Sub-investigator); Meredith Wessner (The Ohio State University, Sub-investigator); Nicole Vrettos (The Ohio State University, Sub-investigator); Punit Agrawal (The Ohio State University, Sub-investigator); Rebecca A. Davis (The Ohio State University, Sub-investigator); Sandra Kostyk (The Ohio State University, Sub-investigator); Maria Kataki (The Ohio State University, Sub-investigator); Jennifer A. Icenhour (The Ohio State University, Sub-investigator); Nicoleta Stoicea (The Ohio State University, Sub-investigator); Erin Keddie Quinn (The Ohio State University, Sub-investigator); Jennifer L. Grothause (The Ohio State University, Sub-investigator); Madelyn M. Deroche (The Ohio State University, Sub-investigator); Renee L. Kovesci (The Ohio State University, Sub-investigator); Stephen Salloway, MD, MS (Butler Hospital, PI); Ann E. Mikos (Butler Hospital, Sub-investigator); Betty Blackham (Butler Hospital, Sub-investigator); Cheryl Kechichian (Butler Hospital, Sub-investigator); Denise Jerue (Butler Hospital, Sub-investigator); Diane Medeiros (Butler Hospital, Sub-investigator); Diane Monast (Butler Hospital, Sub-investigator); Kenneth C. Rickler (Butler Hospital, Sub-investigator); Michelle L. Gardner (Butler Hospital, Sub-investigator); Nicole C. Robbins Mclaughlin (Butler Hospital, Sub-investigator); Patricia S. Read (Butler Hospital, Sub-investigator); Paul Malloy (Butler Hospital, Sub-investigator); Richard T. Marsland (Butler Hospital, Sub-investigator); Virginia J. Sofios (Butler Hospital, Sub-investigator); Wendy Fennelly (Butler Hospital, Sub-investigator); Marcello Fernando Di Carli (Butler Hospital, Sub-investigator); Lincoln P. Tirpaeck (Butler Hospital, Sub-investigator); Christopher J. Maxwell (Butler Hospital, Sub-investigator); Keith A. Johnson (Butler Hospital, Sub-investigator); Irene Piryatinsky (Butler Hospital, Sub-investigator); Jennifer M. Primack (Butler Hospital, Sub-investigator); Kerry L. Mello (Butler Hospital, Sub-investigator); Ronald Schwartz, MD (Hattiesburg Clinic, PI); C. Scott Lynn (Hattiesburg Clinic, Sub-investigator); Cheryl T. Warren (Hattiesburg Clinic, Sub-investigator); Danielle L. Burks (Hattiesburg Clinic, Sub-investigator); Jeffrey J. Schneider (Hattiesburg Clinic, Sub-investigator); Julia G. Starrett (Hattiesburg Clinic, Sub-investigator); Sarah S. Crimmins (Hattiesburg Clinic, Sub-investigator); Tiffany May (Hattiesburg Clinic, Sub-investigator); Wendell R. Helveston (Hattiesburg Clinic, Sub-investigator); Jeffrey Gitt, DO (HOPE Research Institute, PI); Brandy J. Carothers (HOPE Research Institute, Sub-investigator); Lynn C. Bessette (HOPE Research Institute, Sub-investigator); Justin Xavier Mussomeli (HOPE Research Institute, Sub-investigator); Goran Stankovic (HOPE Research Institute, Sub-investigator); Iram Khan (HOPE Research Institute, Sub-investigator); Jonathon Hamre (HOPE Research Institute, Sub-investigator); Nadira Trncic (HOPE Research Institute, Sub-investigator); Nicole Pawenski (HOPE Research Institute, Sub-investigator); Andrew Keegan, MD (The Roskamp Institute, Inc., PI); Anne C. McMearty (The Roskamp Institute, Inc., Sub-investigator); Julia A. Parrish (The Roskamp Institute, Inc., Sub-investigator); Yahdinah Alvarez (The Roskamp Institute, Inc., Sub-investigator); Crawford T. Hawkins (The Roskamp Institute, Inc., Sub-investigator); Deborah Anne Burke (The Roskamp Institute, Inc., Sub-investigator); Carol Rickard (The Roskamp Institute, Inc., Sub-investigator); Cheryl L. Rindfleisch (The Roskamp Institute, Inc., Sub-investigator); Marianne Koepf (The Roskamp Institute, Inc., Sub-investigator); Sylvia S. Goodman Baker (The Roskamp Institute, Inc., Sub-investigator); Janette M. Girard (The Roskamp Institute, Inc., Sub-investigator); Carl Howard Sadowsky, MD (Premiere Research Institute, PI); Jose Antonio Zuniga (Premiere Research Institute, Sub-investigator); Paul K. Winner (Premiere Research Institute, Sub-investigator); Walter C. Martinez (Premiere Research Institute, Sub-investigator); Pierre Tariot, MD (Banner Alzheimer’s Institute, PI); Anna Danuta Burke (Banner Alzheimer’s Institute, Sub-investigator); Elizabeth Watson Salomon (Banner Alzheimer’s Institute, Sub-investigator); Helle Brand (Banner Alzheimer’s Institute, Sub-investigator); Lisa Cooper, RN (Banner Alzheimer’s Institute, Sub-investigator); Marcella Baker (Banner Alzheimer’s Institute, Sub-investigator); Adam S. Fleisher (Banner Alzheimer’s Institute, Sub-investigator); Roy Yaari (Banner Alzheimer’s Institute, Sub-investigator); Sheila Z. Vadovicky (Banner Alzheimer’s Institute, Sub-investigator); Daniel J. Bandy (Banner Alzheimer’s Institute, Sub-investigator); Carolyn Langlois (Banner Alzheimer’s Institute, Sub-investigator); Jessica Z. Langbaum (Banner Alzheimer’s Institute, Sub-investigator); Nellie M. High (Banner Alzheimer’s Institute, Sub-investigator); Benny Lynn Barnhart, MD (Grayline Clinical Drug Trials, PI); Ayres Robert Cermin (Grayline Clinical Drug Trials, Sub-investigator); Daniel C. Elbaum (Grayline Clinical Drug Trials, Sub-investigator); Paul C. Guthrie (Grayline Clinical Drug Trials, Sub-investigator); Susan E. Toole (Grayline Clinical Drug Trials, Sub-investigator); Garland R. Dean (Grayline Clinical Drug Trials, Sub-investigator); Jennifer N. Sharer (Grayline Clinical Drug Trials, Sub-investigator); Kathy M. Castro (Grayline Clinical Drug Trials, Sub-investigator); Mary J. Ross (Grayline Clinical Drug Trials, Sub-investigator); Millicent A. McDonald (Grayline Clinical Drug Trials, Sub-investigator); Tonya E. Rinehart (Grayline Clinical Drug Trials, Sub-investigator); John Paul Nardandrea, Jr., MD (Renstar Medical Research, PI); Deanna L. Buffill (Renstar Medical Research, Sub-investigator); Lynn Sharon Sorrentino (Renstar Medical Research, Sub-investigator); Mary C. Fischer Standley (Renstar Medical Research, Sub-investigator); Patricia Jane Riddle (Renstar Medical Research, Sub-investigator); Stephanie A. Wisser (Renstar Medical Research, Sub-investigator); Juan C. Yordan (Renstar Medical Research, Sub-investigator); Lance Y. Kim (Renstar Medical Research, Sub-investigator); Mark Lewis Patterson (Renstar Medical Research, Sub-investigator); Sidney Ernest Clevinger (Renstar Medical Research, Sub-investigator); Lynn M. Craggs (Renstar Medical Research, Sub-investigator); Michael James Biunno, MD (Louisiana Research Associates, Inc., PI); Nisha Talwar (Louisiana Research Associates, Inc., Sub-investigator); Timothy D. Kemery (Louisiana Research Associates, Inc., Sub-investigator); Angela N. Traylor (Louisiana Research Associates, Inc., Sub-investigator); Bruno Jubelin (Louisiana Research Associates, Inc., Sub-investigator); Ramon Vargas (Louisiana Research Associates, Inc., Sub-investigator); Robert Allan Dahmes (Louisiana Research Associates, Inc., Sub-investigator); Marie Siegler (Louisiana Research Associates, Inc., Sub-investigator); James Gary Booker, MD (J. Gary Booker, MD, APMC, PI); Diane E. Cox (J. Gary Booker, MD, APMC, Sub-investigator); Michelle Harrison (J. Gary Booker, MD, APMC, Sub-investigator); Keith Bradford Kessel (J. Gary Booker, MD, APMC, Sub-investigator); Scott Seawright (J. Gary Booker, MD, APMC, Sub-investigator); Viola Burr (J. Gary Booker, MD, APMC, Sub-investigator); Thomas W. Arnold, MD (Neurology Clinic, PC, PI); Lee S. Stein (Neurology Clinic, PC, Sub-investigator); Barbara Cape O'Brien (Neurology Clinic, PC, Sub-investigator); Kendrick Knoll Henderson (Neurology Clinic, PC, Sub-investigator); Robert H. Segal (Neurology Clinic, PC, Sub-investigator); Ronald J. Bradley (Neurology Clinic, PC, Sub-investigator); Gregory L. Johnson, MD (Methodist Center for Senior Health, PI); Charles W. Lagoski (Methodist Center for Senior Health, Sub-investigator); David A. Priddy (Methodist Center for Senior Health, Sub-investigator); Ishani S. Ali (Methodist Center for Senior Health, Sub-investigator); Tracy L. Rennie (Methodist Center for Senior Health, Sub-investigator); Anita J. Coon (Methodist Center for Senior Health, Sub-investigator); Janet E. Madden (Methodist Center for Senior Health, Sub-investigator); Linda S. Kunz (Methodist Center for Senior Health, Sub-investigator); Susan L. Copp (Methodist Center for Senior Health, Sub-investigator); Joseph Allen Kwentus, MD (Precise Research Centers, PI); Alanna Wright (Precise Research Centers, Sub-investigator); Danny R. Rochelle II (Precise Research Centers, Sub-investigator); Mandy L. Wright (Precise Research Centers, Sub-investigator); Sara L. Hall (Precise Research Centers, Sub-investigator); Shungu Nhema (Precise Research Centers, Sub-investigator); Karen S. Richardson (Precise Research Centers, Sub-investigator); Kristen M. Bevill (Precise Research Centers, Sub-investigator); Connie N. Jenkins (Precise Research Centers, Sub-investigator); David Ira Margolin, MD, PhD (Margolin Brain Institute, PI); Heidi Hamel (Margolin Brain Institute, Sub-investigator); Lori San Agustin (Margolin Brain Institute, Sub-investigator); Shannon Birges (Margolin Brain Institute, Sub-investigator); Tamanjit Basi (Margolin Brain Institute, Sub-investigator); Jann J. Margolin (Margolin Brain Institute, Sub-investigator); Jean H. Wall (Margolin Brain Institute, Sub-investigator); Gary Gerard, MD (Neurology and Neuroscience Center of Ohio, PI); Daniel J. Sanza (Neurology and Neuroscience Center of Ohio, Sub-investigator); Julie Bukowski (Neurology and Neuroscience Center of Ohio, Sub-investigator); Pauline J. Gerard (Neurology and Neuroscience Center of Ohio, Sub-investigator); James H. Long, Jr., MD (Four Rivers Clinical Research, Inc., PI); Cynthia A. Bowman-Stroud (Four Rivers Clinical Research, Inc., Sub-investigator); Dana M. Sowash (Four Rivers Clinical Research, Inc., Sub-investigator); Darrell B. Leslie (Four Rivers Clinical Research, Inc., Sub-investigator); Erin Parrott (Four Rivers Clinical Research, Inc., Sub-investigator); Kristy Harris (Four Rivers Clinical Research, Inc., Sub-investigator); Melissa Wentworth (Four Rivers Clinical Research, Inc., Sub-investigator); Sarah E. Van Meter (Four Rivers Clinical Research, Inc., Sub-investigator); Shanna M. Leslie (Four Rivers Clinical Research, Inc., Sub-investigator); Susan Rames (Four Rivers Clinical Research, Inc., Sub-investigator); Thesha A. Jones (Four Rivers Clinical Research, Inc., Sub-investigator); Jesse Wallace (Four Rivers Clinical Research, Inc., Sub-investigator); John Edward Grubbs (Four Rivers Clinical Research, Inc., Sub-investigator); Joseph Matt Pittard (Four Rivers Clinical Research, Inc., Sub-investigator); Paul J. Grumley (Four Rivers Clinical Research, Inc., Sub-investigator); Anton P. Porsteinsson, MD (University of Rochester Medical Center, PI); Andrew J. Porter (University of Rochester Medical Center, Sub-investigator); Asa A. Widman (University of Rochester Medical Center, Sub-investigator); Bonnie S. Goldstein (University of Rochester Medical Center, Sub-investigator); Carol A. Cole (University of Rochester Medical Center, Sub-investigator); Connie M. Brand (University of Rochester Medical Center, Sub-investigator); Kelly C. Stear (University of Rochester Medical Center, Sub-investigator); Kelly Cosman (University of Rochester Medical Center, Sub-investigator); Kimberly S. Martin (University of Rochester Medical Center, Sub-investigator); Michael Hasselberg (University of Rochester Medical Center, Sub-investigator); Nancy A. Kowalski (University of Rochester Medical Center, Sub-investigator); Stephen A. Bean (University of Rochester Medical Center, Sub-investigator); Saleem Ismail (University of Rochester Medical Center, Sub-investigator); Susan Salem-Spencer (University of Rochester Medical Center, Sub-investigator); Sanjay Asthana, MD (University of Wisconsin Medical School, PI); Cynthia McDonnell Carlsson (University of Wisconsin Medical School, Sub-investigator); LeAnn M. DeRungs (University of Wisconsin Medical School, Sub-investigator); J. Jay Fruehling (University of Wisconsin Medical School, Sub-investigator); Carey E. Gleason (University of Wisconsin Medical School, Sub-investigator); Sandra J. Harding (University of Wisconsin Medical School, Sub-investigator); Amy A. Hawley (University of Wisconsin Medical School, Sub-investigator); Sterling C. Johnson (University of Wisconsin Medical School, Sub-investigator); Charles K. Stone (University of Wisconsin Medical School, Sub-investigator); Heidi P. Walaski (University of Wisconsin Medical School, Sub-investigator); Zachary C. Zugin (University of Wisconsin Medical School, Sub-investigator); Gad Marshall, MD (Brigham and Women’s Hospital, PI); Brendon Phillip Boot (Brigham and Women’s Hospital, Sub-investigator); Dorene M. Rentz (Brigham and Women’s Hospital, Sub-investigator); Mykol Larvie (Brigham and Women’s Hospital, Sub-investigator); Nancy J. Donovan (Brigham and Women’s Hospital, Sub-investigator); Rebecca E. Amariglio (Brigham and Women’s Hospital, Sub-investigator); Laura L. Horky (Brigham and Women’s Hospital, Sub-investigator); Marcello Fernando Di Carli (Brigham and Women’s Hospital, Sub-investigator); Reisa A. Sperling (Brigham and Women’s Hospital, Sub-investigator); Scott M. McGinnis (Brigham and Women’s Hospital, Sub-investigator); Keith A. Johnson(Brigham and Women’s Hospital, Sub-investigator); Gustavo Alva, MD (ATP Clinical Research, Inc., PI); Bobby Shih (ATP Clinical Research, Inc., Sub-investigator); Marissa Rabe (ATP Clinical Research, Inc., Sub-investigator); Poonam Nina Banerjee (ATP Clinical Research, Inc., Sub-investigator); Sanjai Thankachen (ATP Clinical Research, Inc., Sub-investigator); Alejandro Alva (ATP Clinical Research, Inc., Sub-investigator); Jason Kellogg (ATP Clinical Research, Inc., Sub-investigator); Janice Ann Knebl, DO, MBA (University of North Texas Health Science Center at Fort Worth, PI); Barbara L. Harty (University of North Texas Health Science Center at Fort Worth, Sub-investigator); Charles Dan Hooper (University of North Texas Health Science Center at Fort Worth, Sub-investigator); Hedieh Davanloo (University of North Texas Health Science Center at Fort Worth, Sub-investigator); James R. Hall (University of North Texas Health Science Center at Fort Worth, Sub-investigator); Lisa M. Alvarez (University of North Texas Health Science Center at Fort Worth, Sub-investigator); Deepti Patki (University of North Texas Health Science Center at Fort Worth, Sub-investigator); Jonathan M. Licht, MD (Coordinated Clinical Research, PI); Adelina Matevosyan (Coordinated Clinical Research, Sub-investigator); Gilda M. Tafreshi (Coordinated Clinical Research, Sub-investigator); Isaac Bakst (Coordinated Clinical Research, Sub-investigator); Lynn De Paur (Coordinated Clinical Research, Sub-investigator); Nozomi Yagi (Coordinated Clinical Research, Sub-investigator); Sayka Ross (Coordinated Clinical Research, Sub-investigator); Guillermo A. Cantu-Reyna (Coordinated Clinical Research, Sub-investigator); Naira Kocharian (Coordinated Clinical Research, Sub-investigator); Luci Barbie (Coordinated Clinical Research, Sub-investigator); Matthew Macaluso, DO (KU-Wichita Clinical Trail Unit, PI); Carla J. Jones (KU-Wichita Clinical Trail Unit, Sub-investigator); Elizabeth B. Campbell (KU-Wichita Clinical Trail Unit, Sub-investigator); Gregory A. Rockers (KU-Wichita Clinical Trail Unit, Sub-investigator); Jessica M. Rudick (KU-Wichita Clinical Trail Unit, Sub-investigator); Kelli A. Omo (KU-Wichita Clinical Trail Unit, Sub-investigator); Lyle E. Baade (KU-Wichita Clinical Trail Unit, Sub-investigator); Russell E. Scheffer (KU-Wichita Clinical Trail Unit, Sub-investigator); Sheldon H. Preskorn (KU-Wichita Clinical Trail Unit, Sub-investigator); Michael Gregory Plopper, MD (Sharp Mesa Vista Hospital, PI); Christopher L. LoCascio (Sharp Mesa Vista Hospital, Sub-investigator); Alan Lee Berkowitz (Sharp Mesa Vista Hospital, Sub-investigator); Claudia A. Prilliman (Sharp Mesa Vista Hospital, Sub-investigator); Fadi Joseph Nicolas (Sharp Mesa Vista Hospital, Sub-investigator); Thomas Anthony Hessling (Sharp Mesa Vista Hospital, Sub-investigator); Mark Dobrine (Sharp Mesa Vista Hospital, Sub-investigator); Robert Gabriel Feldman, MD (Senior Clinical Trials, Inc., PI); Barbara A. Knapp-Colston (Senior Clinical Trials, Inc., Sub-investigator); Kendra Kiehl Feldman (Senior Clinical Trials, Inc., Sub-investigator); Mary Frances Burt (Senior Clinical Trials, Inc., Sub-investigator); Kent Peppard (Senior Clinical Trials, Inc., Sub-investigator); Sanjiv Kumar Sharma, MBBS (Memory Enhancement Center of New Jersey, Inc., PI); Ashley M. Clayton (Memory Enhancement Center of New Jersey, Inc., Sub-investigator); Divya Singh (Memory Enhancement Center of New Jersey, Inc., Sub-investigator); Ed Austin (Memory Enhancement Center of New Jersey, Inc., Sub-investigator); Joan A. Otranto (Memory Enhancement Center of New Jersey, Inc., Sub-investigator); Kaycee C. Doyle (Memory Enhancement Center of New Jersey, Inc., Sub-investigator); Joel Steven Ross (Memory Enhancement Center of New Jersey, Inc., Sub-investigator); Mohammad Reza Bolouri, MD (Alzheimer’s Memory Center, PI); Brandi N. Gladden (Alzheimer’s Memory Center, Sub-investigator); Esther M. Gregg (Alzheimer’s Memory Center, Sub-investigator); Shervin Eshraghi (Alzheimer’s Memory Center, Sub-investigator); Mary Esther M. Gregg (Alzheimer’s Memory Center, Sub-investigator); Susan K. Williams (Alzheimer’s Memory Center, Sub-investigator); Laura P. Wood (Alzheimer’s Memory Center, Sub-investigator); Dennis W. Dietrich, MD (Advanced Neurology Specialists, PI); Mary M. Frank (Advanced Neurology Specialists, Sub-investigator); William E. Henning (Advanced Neurology Specialists, Sub-investigator); Robert Enoch Litman, MD (CBH Health, LLC, PI); Brima Cherif (CBH Health, LLC, Sub-investigator); Deborah Vickerie (CBH Health, LLC, Sub-investigator); Elisa C. Conrad (CBH Health, LLC, Sub-investigator); Florence Gee (CBH Health, LLC, Sub-investigator); Khaled Abdelghany Khalafallah (CBH Health, LLC, Sub-investigator); Lewis C. Wilson (CBH Health, LLC, Sub-investigator); Megan Y. Maiorca (CBH Health, LLC, Sub-investigator); Shobha Latha Solomon (CBH Health, LLC, Sub-investigator); Monica D. Franklin (CBH Health, LLC, Sub-investigator); Michael J. McCartney, MD (ActivMed Practices and Research, Inc., PI); Sandra Jutras (ActivMed Practices and Research, Inc., Sub-investigator); Marc A. Shay (ActivMed Practices and Research, Inc., Sub-investigator); Peter L. Rees (ActivMed Practices and Research, Inc., Sub-investigator); Jonathan Leonard Liss, MD (Medical Research and Health Education Foundation, Inc., PI); Jagdish Sidhpura (Medical Research and Health Education Foundation, Inc., Sub-investigator); Jose Alfonso Canedo (Medical Research and Health Education Foundation, Inc., Sub-investigator); Samuel Harris Markind, MD (Associated Neurologists, PC, PI); Ann Marie Engelman (Associated Neurologists, PC, Sub-investigator); Behzad Habibi Khameneh (Associated Neurologists, PC, Sub-investigator); Courtney E. Kennedy (Associated Neurologists, PC, Sub-investigator); Dawn Marie Onofrio Morsey (Associated Neurologists, PC, Sub-investigator); Dawn Murphy (Associated Neurologists, PC, Sub-investigator); Ellen Torabi (Associated Neurologists, PC, Sub-investigator); Janet Mauro (Associated Neurologists, PC, Sub-investigator); Jonathan Woodhouse (Associated Neurologists, PC, Sub-investigator); Linda P. Mulvihill (Associated Neurologists, PC, Sub-investigator); Melisa Pelikan (Associated Neurologists, PC, Sub-investigator); Erin Lasher Jacobstein (Associated Neurologists, PC, Sub-investigator); Jennifer Prewitt, PsyD (Associated Neurologists, PC, Sub-investigator); Robert Bonwetsch (Associated Neurologists, PC, Sub-investigator); Joseph Soufer, MD (Chase Medical Research, LLC, PI); Carole Torello (Chase Medical Research, LLC, Sub-investigator); Diane F. Rabideau (Chase Medical Research, LLC, Sub-investigator); Leonard I. Goldstein (Chase Medical Research, LLC, Sub-investigator); Staci Berardi (Chase Medical Research, LLC, Sub-investigator); Neil J. Miller (Chase Medical Research, LLC, Sub-investigator); Cindy Tuten, MD (Clinical Study Center of Asheville, LLC, PI); Christine C. Messino (Clinical Study Center of Asheville, LLC, Sub-investigator); Daniel F. Cannon (Clinical Study Center of Asheville, LLC, Sub-investigator); John Newell Dean (Clinical Study Center of Asheville, LLC, Sub-investigator); Juliana R. Turner (Clinical Study Center of Asheville, LLC, Sub-investigator); Krystal Worley (Clinical Study Center of Asheville, LLC, Sub-investigator); Lucian C. Rice, Jr. (Clinical Study Center of Asheville, LLC, Sub-investigator); Peggy Deitz (Clinical Study Center of Asheville, LLC, Sub-investigator); Roberta Naimark (Clinical Study Center of Asheville, LLC, Sub-investigator); Abdullah Dean Sherzai, MD, PhD (Faculty Physicians and Surgeons of Loma Linda University School of Medicine, PI); Ayesha Z. Sherzai (Faculty Physicians and Surgeons of Loma Linda University School of Medicine, Sub-investigator); Sandra E.D. Estrada (Faculty Physicians and Surgeons of Loma Linda University School of Medicine, Sub-investigator); Jerold Mikszewski, MD (Pacific Medical Centers, PI); Lidiya A. Kosmin (Pacific Medical Centers, Sub-investigator); Lisa Olsson (Pacific Medical Centers, Sub-investigator); Scott Malone (Pacific Medical Centers, Sub-investigator); Anne Mai (Pacific Medical Centers, Sub-investigator); Elizabeth A. Vogt (Pacific Medical Centers, Sub-investigator); Danna Lee Jennings, MD (Institute for Neurodegenerative Disorders, PI); David Stewart Russell (Institute for Neurodegenerative Disorders, Sub-investigator); William Travis Ellison, MD (Radiant Research, Inc., PI); John F. Pilch (Radiant Research, Inc., Sub-investigator); Michael Robert Billings (Radiant Research, Inc., Sub-investigator); Michael Christopher DeSantis, MD (Clinical Trials of America, Inc., PI); Joel Carl Kunze (Clinical Trials of America, Inc., Sub-investigator); Larry Alan Dobkin, MD (Clinical Trials Research Services, LLC, PI); Catherine D. Ravella (Clinical Trials Research Services, LLC, Sub-investigator); Clara H. Gautier (Clinical Trials Research Services, LLC, Sub-investigator); Lindsay K. Champ (Clinical Trials Research Services, LLC, Sub-investigator); Norman J. Belt (Clinical Trials Research Services, LLC, Sub-investigator); David Leland Katz (Clinical Trials Research Services, LLC, Sub-investigator); George Larry Rosenberg (Clinical Trials Research Services, LLC, Sub-investigator); Saul R. Berg (Clinical Trials Research Services, LLC, Sub-investigator); Daniel E. Grosz, MD (Pharmacology Research Institute, PI); Clifford R. Feldman (Pharmacology Research Institute, Sub-investigator); Judy L. Morrissey (Pharmacology Research Institute, Sub-investigator); Michelle T. Panlilio (Pharmacology Research Institute, Sub-investigator); Wendy N. Belden (Pharmacology Research Institute, Sub-investigator); Charles S. Wilcox (Pharmacology Research Institute, Sub-investigator); Nader Oskooilar (Pharmacology Research Institute, Sub-investigator); Jonathan Oren Harris, MD (Neurologic Consultants, P.A., PI); Mark E. Todd (Neurologic Consultants, P.A., Sub-investigator); Randi L. Weitz (Neurologic Consultants, P.A., Sub-investigator); Marc A. Swerdloff (Neurologic Consultants, P.A., Sub-investigator); Scott M. Pearlman (Neurologic Consultants, P.A., Sub-investigator); Seth Craig Tarras (Neurologic Consultants, P.A., Sub-investigator); Thomas Craig Hammond (Neurologic Consultants, P.A., Sub-investigator); Todd Alan Rosenzweig (Neurologic Consultants, P.A., Sub-investigator); Margaret V. Scott (Neurologic Consultants, P.A., Sub-investigator); William Alvin McElveen, MD (Bradenton Research Center, Inc., PI); Alison Bradshaw (Bradenton Research Center, Inc., Sub-investigator); Katherine Byerly (Bradenton Research Center, Inc., Sub-investigator); Kathryn Joyce Robertson (Bradenton Research Center, Inc., Sub-investigator); Michelle L. Scott (Bradenton Research Center, Inc., Sub-investigator); Vickie Carol Roman (Bradenton Research Center, Inc., Sub-investigator); Ralph F. Gonzalez (Bradenton Research Center, Inc., Sub-investigator); Stephen Stiles (Bradenton Research Center, Inc., Sub-investigator); Michael Peter Biber, MD (Neurocare, Inc., PI); Vicky Barrios (Neurocare, Inc., Sub-investigator); Martha W. Gilpatrick (Neurocare, Inc., Sub-investigator); Keith A. Johnson (Neurocare, Inc., Sub-investigator); Thomas Raymond Vidic, MD (Elkhart Clinic, LLC, PI); Anne Siatczynski (Elkhart Clinic, LLC, Sub-investigator); Julie Pelletier (Elkhart Clinic, LLC, Sub-investigator); Suying Wu (Elkhart Clinic, LLC, Sub-investigator); Gary W. Elliot (Elkhart Clinic, LLC, Sub-investigator); Jaron Lerner Winston, MD (Senior Adults Specialty Research, PI); Cliff Whitehead (Senior Adults Specialty Research, Sub-investigator); Dorothy Y. Walker (Senior Adults Specialty Research, Sub-investigator); Laura L. Eisenberg (Senior Adults Specialty Research, Sub-investigator); Meredith L. Draper (Senior Adults Specialty Research, Sub-investigator); Amal Chakraburtty, MBBS (Red River Medical Research Center, LLC, PI); Beverly D. Jerden (Red River Medical Research Center, LLC, Sub-investigator); Donna Smela (Red River Medical Research Center, LLC, Sub-investigator); Jane Ottis Teague (Red River Medical Research Center, LLC, Sub-investigator); Jerrod L. Roberts (Red River Medical Research Center, LLC, Sub-investigator); John Christopher Andrus (Red River Medical Research Center, LLC, Sub-investigator); Melanie Aven (Red River Medical Research Center, LLC, Sub-investigator); Melissa W. Hayes (Red River Medical Research Center, LLC, Sub-investigator); Surinder Kaur Randhawa (Red River Medical Research Center, LLC, Sub-investigator); Michael Adam Hassman, DO (Comprehensive Clinical Research, PI); Krista R. Hill (Comprehensive Clinical Research, Sub-investigator); David Ross Hassman (Comprehensive Clinical Research, Sub-investigator); Mary Louise Stedman, MD (Stedman Clinical Trials, LLC, PI); Eugene M. Dagon (Stedman Clinical Trials, LLC, Sub-investigator); Justin Wiedeman (Stedman Clinical Trials, LLC, Sub-investigator); Leslie J. Correa (Stedman Clinical Trials, LLC, Sub-investigator); Ronald E. DeMao (Stedman Clinical Trials, LLC, Sub-investigator); Sally A. Goff (Stedman Clinical Trials, LLC, Sub-investigator); Belen Maria Herrero (Stedman Clinical Trials, LLC, Sub-investigator); Carletta J. Sanders (Stedman Clinical Trials, LLC, Sub-investigator); Lori M. Drum (Stedman Clinical Trials, LLC, Sub-investigator); Kerri Louise Wilks, MD (MD Clinical, PI); Beth Emmie Safirstein (MD Clinical, Sub-investigator); Matthews Weber Gwynn, MD (NeuroTrials Research, Inc., PI); Christina Lynn Mayville (NeuroTrials Research, Inc., Sub-investigator); Joan T. Burk (NeuroTrials Research, Inc., Sub-investigator); Dennis Michael Lacey (NeuroTrials Research, Inc., Sub-investigator); Russell P. Rosenberg (NeuroTrials Research, Inc., Sub-investigator); Nader Oskooilar, MD, PhD (Pharmacology Research Institute, PI); Jeffrey D. Litzinger (Pharmacology Research Institute, Sub-investigator); Jennifer L. Pack (Pharmacology Research Institute, Sub-investigator); Kimberly A. Guevarra (Pharmacology Research Institute, Sub-investigator); Mellissa M. Henry (Pharmacology Research Institute, Sub-investigator); P. Bacon (Pharmacology Research Institute, Sub-investigator); Charles S. Wilcox (Pharmacology Research Institute, Sub-investigator); Don Fredric DeFrancisco (Pharmacology Research Institute, Sub-investigator); Barbara A. Katz (Pharmacology Research Institute, Sub-investigator); My-Linh Tong (Pharmacology Research Institute, Sub-investigator)
