# Supplementary material for: Bapineuzumab for mild to moderate Alzheimer’s disease in two global, randomized, phase 3 trials
Source: Alzheimers Res Ther. 2016 May 12;8:18. doi: 10.1186/s13195-016-0189-7 (PMC4866415; doi:10.1186/s13195-016-0189-7)
Supplement: Additional file 2: — Inclusion and exclusion criteria. Inclusion and exclusion criteria, similar to those used in the 301/302 studies. (DOCX 18 kb) [file 13195_2016_189_MOESM2_ESM.docx]

**Inclusion Criteria**

- Signed and dated written informed consent. The subject’s caregiver must also consent to participate in the study
- Man or surgically sterile or postmenopausal woman, aged ≥50 to <89 years
- Diagnosis of probable Alzheimer’s disease (AD) according to the National Institute of Neurological and Communicative Disorders and Stroke and Alzheimer’s Disease and Related Disorders Association (NINCDS-ADRDA) criteria
- Mini–Mental State Examination score of 16 to 26, inclusive
- Rosen Modified Hachinski Ischemic score ≤4
- Lives at home with appropriate caregiver capable of accompanying the subject on all clinic visits, or community dwelling with caregiver capable of accompanying the subject on all clinic visits and visiting with the subject approximately 5 times per week for the duration of the study
- Screening visit brain magnetic resonance imaging (MRI) scan consistent with the diagnosis of AD
- Fluency in local language and evidence of adequate premorbid intellectual functioning
- Adequate visual and auditory abilities to perform all aspects of the cognitive and functional assessments
- Receiving stable doses of medication(s) for the treatment of nonexcluded medical condition(s) for at least 30 days prior to screening, and, if treated with cholinesterase inhibitors and/or memantine, the subject is maintained on a stable dose regimen for at least 120 days prior to screening and is free of any clinically important side effects attributable to the drug and the subject and caregiver agree that, barring unforeseen circumstances, they will continue the same regimen for the duration of the trial
- The subject and caregiver are likely to be able to participate in all scheduled evaluations and complete all required tests
- Study 3001: Carrier of apolipoprotein E (ApoE) ε4 allele according to genotyping at screening (ie, has 1 or 2 copies of ApoE ε4); Study 3000: Noncarrier of ApoE ε4 according to genotyping at screening (ie, has zero copies of ApoE ε4)

**Exclusion Criteria**

- Significant neurologic disease, other than AD, that may affect cognition
- History of or screening visit brain MRI scan indicative of any other significant abnormality, including but not limited to multiple microhemorrhages (2 or more), history or evidence of a single prior hemorrhage >1 cm^3^, multiple lacunar infarcts (2 or more) or evidence of a single prior infarct >1 cm^3^, evidence of a cerebral contusion, encephalomalacia, aneurysms, vascular malformations, subdural hematoma, or space-occupying lesions (eg, arachnoid cysts or brain tumors, such as meningioma)
- Current presence of a clinically important major psychiatric disorder or symptom
- Current clinically important systemic illness
- History of clinically evident stroke or history of clinically important carotid or vertebrobasilar stenosis or plaque
- History of seizures, excluding febrile seizure in childhood
- History or evidence of any clinically important autoimmune disease or disorder of the immune system
- Clinically important infection within the last 30 days
- Treatment with immunosuppressive medications within the last 90 days or chemotherapeutic agents for malignancy within the last 3 years
- Myocardial infarction within the last 2 years
- History of cancer within the last 5 years, with the exception of nonmetastatic basal cell carcinoma and squamous cell carcinoma of the skin
- Uncontrolled hypertension within 6 months prior to screening
- Other clinically important abnormality
- Current use of anticonvulsant agents for seizures, antiparkinson agents, or anticoagulant medications, or regular use of opioid pain relievers
- Current use of prescription or nonprescription medication for cognitive enhancement other than cholinesterase inhibitors and memantine
- Discontinuation of cholinesterase inhibitors, memantine, cognitive-enhancing agents, or drugs that potentially affect cognition in the 60 days prior to screening
- Unless the subject is maintained on a stable dose regimen for at least 30 days prior to screening, use of any other medications with the potential to affect cognition other than cholinesterase inhibitors or memantine
- Use of experimental medications for AD or any other investigational medications or devices for treatment of indications other than AD within 60 days prior to screening or within 5 half-lives of use of such a medication prior to screening, whichever is longer
- Any prior experimental treatment with AN1792, bapineuzumab, ACC-001, or other experimental immunotherapeutic or vaccine for AD
- Any prior treatment with a biological product other than for the treatment of AD within the last 3 years, with the exception of routine vaccines that are commercially available
- Woman of childbearing potential
- Any contraindications to a brain MRI scan
- Inadequate venous access to allow intravenous drug delivery or multiple blood draws
