# Supplementary material for: Bapineuzumab for mild to moderate Alzheimer’s disease in two global, randomized, phase 3 trials
Source: Alzheimers Res Ther. 2016 May 12;8:18. doi: 10.1186/s13195-016-0189-7 (PMC4866415; doi:10.1186/s13195-016-0189-7)
Supplement: Additional file 3: — Prespecified outcomes in apolipoprotein E ε4 carrier and noncarrier trials. All prespecified endpoints are listed. (DOCX 18 kb) [file 13195_2016_189_MOESM3_ESM.docx]

**Prespecified outcomes in apolipoprotein E (ApoE) ε4 carrier and noncarrier trials**

| Co-primary | - Change from baseline to week 78 for ADAS-Cog/11 total score - Change from baseline to week 78 for DAD total score |
| --- | --- |
| Key secondary biomarker | - Change from baseline to week 71 in brain amyloid burden (average standardized uptake value ratio in prespecified ROI), assessed by PiB-PET imaging in a subset of subjects - Change from baseline to week 71 in p-tau levels in the CSF in a subset of subjects - Change from baseline to week 71 in brain volume, assessed by MRI BBSI in a subset of subjects |
| Key secondary divergence of effect | - Divergence of effect on ADAS-Cog/11 total scores from week 39 to 78 between bapineuzumab and placebo - Divergence of effect on DAD total scores from week 39 to 78 between bapineuzumab and placebo |
| Key secondary clinical and health outcomes | - Time to median placebo deterioration on ADAS-Cog/11 total score - Time to median placebo deterioration on DAD total score |
| Other secondary outcomes | - Proportions of subjects with worsening from baseline to week 78 of at most 0, 3, and 7 points on ADAS-Cog/11 total score - Proportions of subjects with worsening from baseline to week 78 of at most 0, 6, and 12 points on DAD total score - ADAS-Cog/11 total score cumulative response curves (reverse cumulative distribution functions) - DAD total score cumulative response curves (reverse cumulative distribution functions) |
| Exploratory efficacy: Other neuropsychiatric or cognitive | - Change from baseline to week 78 in Neuropsychological Test Battery Z-score - Change from baseline to week 78 for Neuropsychiatric Inventory total score - Change from baseline to week 78 for Mini–Mental State Examination total score |
| Exploratory efficacy: Additional biomarker | - Change from baseline to week 71 in CSF total tau levels in a subset of subjects - Change from screening to week 71 in whole-brain volume, ventricular volume, ventricular boundary shift integral, and hippocampal boundary shift integral, as assessed by MRI in a subset of subjects - Change from baseline to week 71 in brain amyloid burden in individual ROI as assessed by PiB-PET imaging in a subset of subjects |
| Exploratory efficacy: Correlations | - Correlations between change from baseline to week 78 in ADAS-Cog/11 total score and DAD total score versus change from baseline to week 71 in key secondary biomarkers (PiB-PET ROI average, CSF p-tau, and BBSI) |
| Exploratory efficacy: Pharmacokinetic and pharmacodynamic | - Serum bapineuzumab concentrations at specified weeks - Serum bapineuzumab population PK parameters, clearance, and volume of distribution, and the percentage coefficient of variation of these parameters - Extrinsic and intrinsic factors that may contribute to the variability observed in the population PK parameters - CSF concentrations of bapineuzumab at week 71 for a subset of subjects - Change from baseline to week 71 in amyloid beta concentrations in the CSF and plasma for a subset of subjects |
| Exploratory efficacy: Immunogenicity | - Serum anti-bapineuzumab antibody levels in all subjects - CSF anti-bapineuzumab antibody levels in a subset of subjects |
| Exploratory efficacy: Additional health outcomes | - Subject resource utilization and needed caregiver time using RUD-Lite v2.4 in subjects with mild to moderate AD, including change from baseline scores in the primary and secondary caregiver time component of RUD-Lite v2.4 to week 78 - Health-related QoL in subjects with mild to moderate AD, determined by the change from baseline scores in QoL AD scores to week 78 - Health Utilities Index total score |
| Safety | - Incidence and severity of treatment-emergent adverse events - Clinically important changes in safety assessment results, including, as appropriate, vital signs, weight, clinical laboratory tests, electrocardiograms, brain MRI scans, and physical and neurologic examinations |

AD = Alzheimer’s disease; ADAS-Cog/11 = 11-item Alzheimer’s Disease Assessment Scale–Cognitive subscale; BBSI = brain boundary shift integral; CSF = cerebrospinal fluid; DAD = Disability Assessment for Dementia; MRI = magnetic resonance imaging; PET = positron emission tomography; PiB = ^11^C-Pittsburgh compound B; PK = pharmacokinetic; p-tau = phosphorylated tau; QoL = quality of life; ROI = region of interest; RUD-Lite v2.4 = Resource Utilization in Dementia, version 2.4.
