# Supplementary material for: Bapineuzumab for mild to moderate Alzheimer’s disease in two global, randomized, phase 3 trials
Source: Alzheimers Res Ther. 2016 May 12;8:18. doi: 10.1186/s13195-016-0189-7 (PMC4866415; doi:10.1186/s13195-016-0189-7)
Supplement: Additional file 4: Table S1. — Change from baseline to week 78 by MMSE score at baseline (ApoE ε4 carriers and noncarriers). Table showing analysis of ADAS-Cog/11 and DAD scores by disease severity. (DOCX 14 kb) [file 13195_2016_189_MOESM4_ESM.docx]

**Change from baseline to week 78 by MMSE score at baseline (ApoE ε4 carriers and non-carriers)**

|  | PBO | BAP 0.5 | | | BAP 1.0 | | |
| --- | --- | --- | --- | --- | --- | --- | --- |
|  | LS mean (SE) | LS mean (SE) | Difference of LS means  (95% CI) | *p* value | LS mean (SE) | Difference of LS means  (95% CI) | *p* value |
| **ApoE ε4 carriers: Mild patients, MMSE ≥22** | | | | | | | |
| ADAS-Cog/11 total score (n = 145, 202) | 4.2 (0.62) | 5.0 (0.51) | 0.81 (–0.77, 2.39) | 0.313 | n/a | n/a | n/a |
| DAD total score (n = 146, 198) | –10.7 (1.28) | –10.1 (1.07) | 0.6 (–2.69, 3.88) | 0.721 | n/a | n/a | n/a |
| **ApoE ε4 carriers: Moderate patients, MMSE <22** | | | | | | | |
| ADAS-Cog/11 total score (n = 49, 40, 36) | 4.5 (0.89) | 3.0 (1.00) | –1.54  (–4.18, 1.10) | 0.251 | 5.2 (1.06) | 0.70  (–2.04, 3.43) | 0.615 |
| DAD total score  (n = 49, 39, 36) | –7.6 (1.75) | –9.8 (1.97) | –2.23  (–7.42, 2.96) | 0.397 | –13.6 (2.06) | –6.01  (–11.34, –0.68) | 0.027 |
| **ApoE ε4 noncarriers: Mild patients, MMSE ≥22** | | | | | | | |
| ADAS-Cog/11 total score (n = 155, 212) | 10.0 (0.69) | 9.40 (0.58) | –0.61 (–2.40, 1.17) | 0.498 | n/a | n/a | n/a |
| DAD total score (n=155, 213) | –18.8 (1.47) | –19.1 (1.24) | –0.31 (–4.08, 3.47) | 0.873 | n/a | n/a | n/a |
| **ApoE ε4 noncarriers: Moderate patients, MMSE <22** | | | | | | | |
| ADAS-Cog/11 total score (n = 75, 67, 64) | 10.5 (0.90) | 8.2 (0.97) | –2.39  (–4.98, 0.21) | 0.071 | 9.7 (0.99) | –0.85  (–3.48, 1.78) | 0.525 |
| DAD total score  (n = 76, 67, 66) | –22.0 (1.89) | –18.1 (2.05) | 3.95  (–1.54, 9.44) | 0.158 | –15.8 (2.08) | 6.20  (0.67, 11.72) | 0.028 |

ADAS-Cog/11 = 11-item Alzheimer’s Disease Assessment Scale–Cognitive subscale; ApoE = apolipoprotein E; BAP = bapineuzumab; DAD = Disability Assessment for Dementia; LS = least squares; MMSE = Mini–Mental State Examination; PBO = placebo.

^a^ADAS-Cog/11 total score range is 0 (least impairment) to 70 (most impairment); a negative change from baseline indicates an improvement.

^b^DAD total score range is 0 to 100, with higher scores indicating better function; a positive change from baseline indicates an improvement.
