# Supplementary material for: Bapineuzumab for mild to moderate Alzheimer’s disease in two global, randomized, phase 3 trials
Source: Alzheimers Res Ther. 2016 May 12;8:18. doi: 10.1186/s13195-016-0189-7 (PMC4866415; doi:10.1186/s13195-016-0189-7)
Supplement: Additional file 5: — List of independent ethics committees or institutional review boards. Complete list of independent ethics committees for all sites that screened subjects for the ApoE ε4 carrier study. (PDF 134 kb) [file 13195_2016_189_MOESM5_ESM.pdf]

16.1.3 List of Independent Ethics Committees or Institutional Review Boards – Independent Ethics Committees

A list of the following is included:

- The List of Independent Ethics Committees, sorted by country and site, for sites that screened subjects.

090177e184765be1\Approved\Approved On: 16-Aug-2013 07:04

090177e184765be1\Approved\Approved On: 16-Aug-2013 07:04

Independent Ethics Committees

Site number	Name and Address of Committee
**Argentina**	
169	Comite de Etica de la Investigacion del CEMIC<br>Galvan 4102,<br>Buenos Aires C1431FWO
172	Comite de Etica de Protocolos de Investigacion (C.E.P.I.) Hospital Italiano,<br>Gascon 450 (C1181ACH),<br>Buenos Aires
173	Comite de Etica Independiente en Investigacion<br>Clinica Larrea 1381- 3°A<br>Buenos Aires C1117ABK
174	Comité de Ética, Investigación y Docencia, Sanatorio Prof. León S. Morra S.A Av. Sagrada Familia esq. Nazaret,<br>Córdoba, Córdoba X5009BIN
**Australia**	
52<br>56	Northern Sydney / Central Coast Area Health Service HREC Human Research Ethics Committee,<br>Royal North Shore Hospital,<br>Level 2 Building 51,<br>Pacific Hwy,<br>St Leonards, NSW 2065
53	Austin Health Human Research Ethics Committee<br>145 Studley Road<br>Heidelberg, Vic 3084
55	Hollywood Private Hospital Research Ethics Committee 101 Monash Avenue,<br>Nedlands, WA 6009

Site number	Name and Address of Committee
**Australia**	
57	Central Northern Adelaide Health Service Ethics of Human Research Committee (TQEH & LMH) The Queen Elizabeth Hospital,<br>28 Woodville Road,<br>Woodville South, SA 5011
58	Royal Adelaide Hospital Research Ethics Committee<br>Royal Adelaide Hospital,<br>Level 3, Hanson Centre,<br>North Terrace,<br>Adelaide, SA 5000
267	BHS & SJOG Human Research Ethics Committee<br>PO Box 577,<br>Drummond Street North,<br>Ballarat, VIC 3353
**Austria**	
140<br>141<br>142<br>151	Ethik Kommission der Medizinischen Universitaet Wien und des Allgemeinen Krankenhauses der Stadt Wien/AKH,<br>Borschkegasse 8b/E 06,<br>(Dienstzimmergebaude, BT 68),<br>Wien A-1090
**Belgium**	
46	Commissie Medische Ethiek Toetsingscommissie,<br>Herestraat 49 3000 Leuven
47<br>48<br>49<br>265<br>272	Commissie Medische Ethiek van de Universitaire Ziekenhuizen KU Campus Gasthuisberg E330<br>Herestraat 49,<br>Leuven 3000

090177e184765be1\Approved\Approved On: 16-Aug-2013 07:04

Site number	Name and Address of Committee
**Chile**	
126<br>176	Comite Etico Cientifico, Servicio de Salud<br>Metropolitano Oriente Av. Salvador 364<br>Providencia,<br>Santiago, RM 7500922
**Croatia**	
248	Central Ethics Committee Agency for Medicinal<br>Products and Medical Devices<br>Ksaverska c. 4,<br>Zagreb 10000
**Finland**	
3<br>6	Pohjois-Savon sairaanhoitopiirin ky Tutkimuseettinen<br>toimikunta<br>Rakennus 10 (4. krs)<br>PL 1777,<br>Kuopio 70211

090177e184765be1\Approved\Approved On: 16-Aug-2013 07:04

Site number	Name and Address of Committee
**France**	
109<br>110<br>111<br>112<br>114<br>115<br>116<br>117<br>118<br>119<br>120<br>121<br>122<br>123<br>124<br>125<br>157<br>158<br>291	CPP Ile de France 3 Hôpital Tarnier Cochin<br>89, rue d'Assas,<br>Paris 75006
**Germany**	
97<br>98<br>101<br>103<br>292<br>315	Ethik-Kommission der Fakultaet fuer Medizin der Technischen Universitaet Muenchen Ismaninger Str. 22,<br>Muenchen 81675
**Italy**	
29	Comitato Etico Indipendente dell'IRCCS Fondazione S. Lucia di Roma<br>Via Ardeatina, 306,<br>Roma 00179

090177e184765be1\Approved\Approved On: 16-Aug-2013 07:04

Site number	Name and Address of Committee
**Italy**	
33	Comitato Etico della Fondazione IRCCS Istituto Neurologico Carlo Besta di Milano<br>Via Celoria, 11,<br>Milano 20133
34	Comitato Etico Comitato Etico dell'Azienda Ospedaliero Universitaria<br>Ospedali Riuniti Umberto I - G.M. Lancisi - G. Salesi di ancona<br>Via Conca, 71<br>Ancona 60126
37	Comitato Etico Locale per la Sperimentazione Clinica dei Medicinali dell'Azienda Ospedaliera Universitaria Senese di Siena<br>c/o UOC Farmacia AOUS<br>Viale Bracci,<br>Siena 53100
38	Comitato Etico dell'Universita' degli Studi Gabriele D'Annunzio e della ASL 2 Lanciano-Vasto-Chieti di Chieti<br>Via dei Vestini, 31,<br>Chieti 66100
39	Comitato Etico Azienda Spedali Civili di Brescia<br>Piazzale Spedali Civili, 1<br>Brescia 25123
40	Comitato Etico Dell'azienda<br>Ospedaliera Cannizzaro Di Catania Via<br>Messina, 829<br>Catania 95126

090177e184765be1\Approved\Approved On: 16-Aug-2013 07:04

Site number	Name and Address of Committee
**Italy**	
41	Comitato Etico dell'Azienda Ospedaliero Universitaria Policlinico Vittorio Emanuele di Catania Via Santa Sofia, 78<br>Catania 95123
90	Comitato Etico Azienda Ospedaliera San Gerardo di Monza Via Pergolesi, 33<br>Monza (MI) 20052
211	Comitato Etico dell'Universita' Campus Bio-Medico di Roma Via Alvaro del Portilllo, 21<br>Roma 00128
**Japan**	
177	National Hospital Organization Kokura Medical Center 10-1 Harugaoka<br>Kokuraminami-ku<br>Kitakyusyu-shi,<br>Fukuoka 802-8533
178	Yokohama City University Medical Center IRB 4-57 Urafunecho<br>Minami-ku,<br>Yokohama, Kanagawa 232-0024
179	National Hospital Organization Minami-Okayama Medical Center IRB 4066<br>Hayashima, Hayashima-cho,<br>Tukubo-Gun, Okayama 701-0304

090177e184765be1\Approved\Approved On: 16-Aug-2013 07:04

Site number	Name and Address of Committee
**Japan**	
180<br>204	Juntendo University Hospital IRB 3-1-3<br>Hongo<br>Bunkyo-ku,<br>Tokyo 113-8431
181	National Hospital Organization Niigata National Hospital IRB 3-52 Akasaka-cho<br>kashiwazaki,<br>Niigata 945-8585
182	National Hospital Organization Chiba-East Hospital 673<br>Nitona-chou<br>Chuou-ku<br>Chiba,<br>Chiba 260-8712
183	Kagawa University Hospital IRB 1750-1<br>Ikenobe<br>Miki-cho<br>Kita-gun,<br>Kagawa 761-0793
184	National Hospital Organization Shizuoka Institute of Epilepsy and Neurological Disorders 886<br>Urushiyama<br>Aoi-ku<br>Shizuoka,<br>Shizuoka 420-8688
185	Osaka City University Hospital 1-5-7,<br>Asahi-machi,<br>Abeno-ku,<br>Osaka,<br>Osaka 545-8586
186	Juntendo Tokyo Koto Geriatric Medical Center IRB 3-3-20<br>Shinsuna<br>Koto-ku,<br>Tokyo 136-0075

090177e184765be1\Approved\Approved On: 16-Aug-2013 07:04

Site number	Name and Address of Committee
**Japan**	
187	Tokyo Medical University Hospital 6-7-1<br>Nishi-Shinjuku<br>Shinjuku-ku,<br>Tokyo 160-0023
188	Rakuwakai Otowa Hospital IRB 2 Chinji-cho,<br>Otowa,<br>Yamashina-ku<br>Kyoto-shi,<br>Kyoto 607-8062
189	Okayama University Hospital IRB 2-5-1 Shikata-cho,<br>Kita-ku<br>Okayama,<br>Okayama 700-8558
190	National Hospital Organization Minami-Kyoto<br>Hospital IRB 11 Ashihara, Naka,<br>Jouyou-shi,<br>Kyoto 610-0113
191	National Hospital Organization Hiroshima-nishi<br>Medical Center 4-1-1<br>Kuba<br>Otake city,<br>Hiroshima 739-0696
192	National Hospital Organization Tokyo National<br>Hospital IRB 3-1-1 Takeoka,<br>Kiyose-shi,<br>Tokyo 204-8585
193	National Hospital Organization Maizuru Medical<br>Center IRB 2410 Aza Yukinaga,<br>Maizuru,<br>Kyoto 625-8502
194	Kansai Medical University Takii Hospital 10-15<br>Fujizono-cho<br>Moriguchi,<br>Osaka 570-8507

090177e184765be1\Approved\Approved On: 16-Aug-2013 07:04

Site number	Name and Address of Committee
**Japan**	
195	Gunma University Hospital 3-39-15<br>Showa-machi<br>Maebashi,<br>Gunma 371-8511
196	Kobe University Hospital IRB 7-5-2<br>kusunoki-cho, Chuo-ku<br>Kobe,<br>Hyogo 650-0017
197	Nagoya City University Hospital IRB Nagoya City University Hospital IRB<br>1<br>Kawasumi Mizuho-cho,<br>Mizuho-ku, Nagoya,<br>Aichi 467-8602
198	Osaka University Hospital IRB Institutional Review Board<br>2-15<br>Yamadaoka<br>Suita,<br>Osaka 565-0871
200	Tokyo Medical University Hachioji Medical Center<br>1163<br>Tatemachi<br>Hachioji,<br>Tokyo 193-09440998
201	Tokyo Metropolitan Health and Medical Treatment Corporation Ebara Hospital 4-5-10 Higashi-Yukigaya<br>Ota-ku,<br>Tokyo 145-0065
202	Nippon Medical School Chiba Hokusoh Hospital IRB<br>1715, Kamagari, Inzaishi<br>Chiba 270-1694

090177e184765be1\Approved\Approved On: 16-Aug-2013 07:04

Site number	Name and Address of Committee
**Japan**	
203	Maebashi Red Cross Hospital IRB 3-21-36<br>Asahi Chou<br>Maebashi,<br>Gunma 371-0014
207	Iwate Medical University Hospital IRB 19-1<br>Uchimaru<br>Morioka-shi,<br>Iwate 020-8505
208	Yachiyo Hospital 2-2-7 Sumiyoshi-cho,<br>Anjo-city,<br>Aichi 446-8510
274	National Hospital Organization Matsumoto Medical<br>Center IRB 811, Kotobukitoyooka,<br>Matsumoto,<br>Nagano 399-0021
275	Tokusyukai Group IRB 1-8-7<br>Kojimachi<br>Chiyoda-ku,<br>Tokyo 102-0083
276	Nippon Medical School Musashi Kosugi Hospital<br>IRB 1-396, Kosugimachi, Nakaharaku,<br>kawasaki,<br>kanagawa 211-8533
277	Shinozuka Hospital IRB 105-1 Shinozuka,<br>Fujioka-city,<br>Gunma 375-0017
281	Kashiwado Hospital IRB 2-21-8 Nagazu, Chuo-ku,<br>Chiba-shi,<br>Chiba 260-86560854
308	Himorogi Psychiatric Institute IRB 1-20-10<br>Sugamo<br>Toshima-ku,<br>Tokyo 170-0002

090177e184765be1\Approved\Approved On: 16-Aug-2013 07:04

Site number	Name and Address of Committee
**Japan**	
309	Kobe City Medical Center West Hospital 2-4<br>Ichiban-cho, Ngata-ku,<br>kobe city,<br>Hyogo 653-0013
**Mexico**	
154	Comite de Etica, Investigacion y Bioseguridad<br>Privada de Aguascalientes,<br>SC Sierra Fria 218<br>Fraccionamiento Bosques del Prado Norte<br>Aguascalientes,<br>Aguascalientes 20127
283	Comite de Ética e Investigación del Hospital y<br>Clínica OCA ,<br>S.A. de C.V Pino Suarez 645 Nte<br>Monterrey,<br>Nuevo Leon 64000
**Netherlands**	
70<br>72<br>73<br>76<br>77<br>80<br>81<br>160<br>285	Vrije Universiteit Medisch Centrum Medisch<br>Ethische Toetsingscommissie<br>Postbus 7057<br>Amsterdam, NH 1007 MB
226	Front Office Vumc (Vumc Ethics Committee) Bureau<br>Medische Zaken<br>Polikliniekgebouw 6Z 196 VU Medisch Centrum -<br>Postbus 7057<br>Amsterdam 1007 MB

090177e184765be1\Approved\Approved On: 16-Aug-2013 07:04

Site number	Name and Address of Committee
**New Zealand**	
54<br>107	Multi Region Ethics Committee Ministry of Health<br>PO Box 5013<br>No. 1 The Terrace,<br>Wellington 6145
**Poland**	
143<br>146<br>147<br>150<br>282	Komisja Bioetyczna przy Warszawskim Uniwersytecie Medycznym w Warszawie ul. Zwirki i Wigury 61,<br>Warszawa 02-091
**Portugal**	
161<br>162<br>163	Comissao de Etica Para a Investigaçao Clinica<br>Parque Da Saude De Lisboa.<br>Av. Do Brasil,<br>53 PAV. 17-A,<br>LISBOA 1749-004
**Serbia**	
42	Ethics Committee of CCS Pasterova 2,<br>Belgrade 11000
44	Ethics Committee of CC Kragujevac Zmaj Jovina 32,<br>Kragujevac 34000
266	Ethics Committe Clinical Centre of Vojvodina<br>Hajduk Veljkova 1-9,<br>Novi Sad 21000

090177e184765be1\Approved\Approved On: 16-Aug-2013 07:04

Site number	Name and Address of Committee
**Slovakia**	
135<br>136<br>137<br>138	Eticka komisia Vseobecnej nemocnice Rimavska Sobota Eticka komisia Vseobecnej nemocnice Rimavska Sobota<br>Srobarova 1,<br>Rimavska Sobota 979 12
**Slovakia**	
136	Eticka komisia UN Bratislava Eticka komisia UN Bratislava<br>Nemocnica Ruzinov<br>Ruzinovska 6,<br>Bratislava 826 06
137	Eticka komisia Psychiatricka nemocnica Michalovce, n.o.<br>Stranany,<br>Michalovce 071 01
138	Eticka komisia Bratislavskeho samospravneho kraja<br>Sabinovska 16<br>P.O.Box 106,<br>Bratislava 820 05
**South Africa**	
83<br>84<br>86<br>87<br>88<br>89	Pharma Ethics 123 Amcor Road<br>Lyttelton Manor,<br>Centurion 0157

090177e184765be1\Approved\Approved On: 16-Aug-2013 07:04

Site number	Name and Address of Committee
**Spain**	
14	Comite Etico de Ensayos Clinicos Agencia de Ensayos Clínicos<br>Hospital Clinic de Barcelona<br>Servicio de Farmacia<br>C/ Villarroel,<br>170 Esc 6B sótano<br>08036 Barcelona
15	Comite Etico De Investigacion Clinica<br>Parc de Salut MAR Comité Ético de Investigación Clínica Parc de Salut MAR<br>Consorci Mar Parc de Salut de Barcelona<br>Parc de Recerca Biomèdica de Barcelona<br>(dcho. 163.03),<br>C/ Doctor Aiguader, 88, 1ª planta<br>08003 Barcelona
16	Hospital de la Santa Creu i Sant Pau Comité Ético de Investigación Clínica<br>Hospital de la Santa Creu i Sant Pau<br>Servicio de Farmacología Clínica<br>Edificio HC,<br>planta 1ª - izquierda.<br>Av. Sant Antoni Mª Claret, 167<br>08025 Barcelona
18	Hospital Mutua de Terrassa Comité Ético de Investigación Clínica<br>Hospital Mutua de Terrassa<br>Servicio de Farmacia<br>Plaça Dr. Robert, 5, Sótano -1<br>08221 Terrassa - Barcelona
19	Hospital Universitario La Paz Secretaría Técnica del Comité Ético de Investigación Clínica - Área 5<br>Hospital Universitario La Paz<br>Edificio Hospital General<br>Pº de la Castellana,<br>261 Plta. 8ª Despacho 818 - 819<br>28046 Madrid

090177e184765be1\Approved\Approved On: 16-Aug-2013 07:04

Site number	Name and Address of Committee
**Spain**	
20	Ceic De Burgos Y Soria Comité Ético de Investigación Clínica<br>Complejo Asistencial Universitario de Burgos<br>Hospital Universitario de Burgos<br>Unidad de Investigación<br>Avda. del Cid, 96<br>09005 Burgos
21	Comite Etico de Investigacion Clinica Comité Ético de Investigación Clínica<br>Area de Gestion de Proyectos - Unidad Administrativa<br>Hospital Universitario 12 de Octubre<br>Instituto de Investigación Hospital 12 de Octubre (i+12)<br>Centro de Actividades Ambulatorias, 6ª Planta - Bloque D<br>Avda. de Córdoba, s/n<br>28041 Madrid
22	Comite Etico de Investigacion Clinica de Euskadi Comité Ético de Investigación Clínica<br>Hospital de Cruces<br>Unidad de Epidemiología Clínica<br>Pabellón de Investigación<br>(detrás del Pabellón de Administración)<br>Plaza de Cruces, 12<br>48903 Baracaldo - Vizcaya

090177e184765be1\Approved\Approved On: 16-Aug-2013 07:04

Site number	Name and Address of Committee
**Spain**	
23	Comité Ético de Investigación Clínica - Área 7<br>Hospital Clínico San Carlos<br>Servicio de Farmacología Clinica<br>Unidad de Coordinación de Ensayos Clínicos<br>1ª planta, Ala norte,<br>puerta G<br>C/ Prof. Martín Lagos, s/n<br>28040 Madrid
24	Comité Ético de Investigación Clínica - Área 2<br>Hospital Universitario de La Princesa<br>C/ Diego de León, 62<br>28006 Madrid
25	Comité Ético de Investigación Clínica<br>Hospital General Universitario de Elche<br>Edificio de Salud Mental 3ª planta<br>C/ Camí de L'Almazara, 11<br>03203 Elche – Alicante
26	Comité Ético de Investigación Clínica<br>Hospital Universitario Virgen de la Arrixaca<br>Planta 0 de Policlínico<br>Ctra. de Madrid - Cartagena, s/n<br>30120 El Palmar - Murcia
51	Complejo Hospitalario de Caceres Comité Ético de Investigación Clínica de Cáceres<br>Hospital Ntra. Sra. de la Montaña<br>Avda. de España, 2<br>10004 Cáceres

090177e184765be1\Approved\Approved On: 16-Aug-2013 07:04

Site number	Name and Address of Committee
**Spain**	
212	Hospital Universitario Ramon y Cajal Comité Ético de Investigación Clínica - Área 4<br>Hospital Universitario Ramón y Cajal<br>Planta menos 2 - dcha.<br>Ctra. de Colmenar Viejo,<br>Km. 9,100<br>28034 Madrid
213	Comite Etico De Investigacion Clinica De Euskadi, CEI-E Comité Ético de Investigación Clínica de la Comunidad Autónoma del País Vasco. CEIC-E<br>Dirección de Farmacia del Departamento de Sanidad<br>C/ Donostia - San Sebastián, 1<br>01010 Vitoria - Alava
214	Comite Etico de Investigacion Clinica de Las Islas Baleares Comité Etico de Investigación Clinica de las Islas Baleares (CEIC-IB)<br>Consellería de Salut I Consum<br>Camino de Jesús, 38<br>07011 Palma de Mallorca - Baleares
**Sweden**	
4<br>215	Regionala etikprovningsnamnden i Stockholm FE 289<br>Stockholm 171 77
**Switzerland**	
127	Ethikkommission beider Basel EKBB Hebelstrasse 53<br>Basel CH-4056
128	University Hospital Geneva Ethic Committee of Internal Medicine<br>Geneva 1211

090177e184765be1\Approved\Approved On: 16-Aug-2013 07:04

Site number	Name and Address of Committee
**Switzerland**	
164	Centre Hospitalier Universitaire Vaudois Comission d'ethique de la Recherche Clinique<br>Rue du Bugnon 21<br>Lausanne 1011
**United Kingdom**	
60	NRES Committee London ¿ South East Room 4W/10, 4th Floor West,<br>Charing Cross Hospital,<br>Fulham Palace Road,<br>London, W6 8RF<br><br>Sheffield Health and Social Care NHS Research Development Unit,<br>Fulwood House,<br>Old Fulwood Road,<br>Sheffield S10 3TH<br><br>STH NHS Foundation Trust Research Department 1st Floor,<br>11 Broomfield Road,<br>Sheffield S10 2SE
61	NRES Committee London ¿ South East Room 4W/10, 4th Floor West,<br>Charing Cross Hospital,<br>Fulham Palace Road,<br>London, W6 8RF<br><br>Cardiff and Vale University Health Board University Hospital of Wales,<br>Commercial Clinical Trials,<br>Research & Development Office,<br>Second Floor, Tower Block 2,<br>Room 3 (2TB2 R3),<br>Heath Park,<br>Cardiff CF14 4XW

090177e184765be1\Approved\Approved On: 16-Aug-2013 07:04

Site number	Name and Address of Committee
**United Kingdom**	
62<br>67<br>68<br>206	NRES Committee London ¿ South East Room<br>4W/10, 4th Floor West,<br>Charing Cross Hospital,<br>Fulham Palace Road,<br>London, W6 8RF
65	Avon and Wiltshire Mental Health Partnership NHS Trust Research and Development,<br>The Blackberry Centre,<br>Blackberry Hill Hospital,<br>Manor Road, Fishponds,<br>Bristol BS16 2EW<br><br>NRES Committee London,<br>South East Health Research Authority,<br>Ground Floor, Skipton House<br>80 London Road<br>London SE1 6LH
66	NHS Greater Glasgow and Community Primary Care, Community & Mental Health LREC South Glasgow & Clyde REC,<br>R&D Directorate,<br>1st Floor - The Tennent Institute,<br>Western Infirmary,<br>38 Church Street,<br>Glasgow G11 6NT<br><br>NRES Committee London ¿ South East Room<br>4W/10, 4th Floor West,<br>Charing Cross Hospital,<br>Fulham Palace Road,<br>London, W6 8RF

090177e184765be1\Approved\Approved On: 16-Aug-2013 07:04

Site number	Name and Address of Committee
**United Kingdom**	
108	Salford and Trafford Local Research Ethics<br>Committee Room 181,1st Floor,<br>Gateway House,<br>Piccadilly South,<br>Manchester M60 7LP<br><br>NRES Committee London ¿ South East Room<br>4W/10, 4th Floor West,<br>Charing Cross Hospital,<br>Fulham Palace Road,<br>London, W6 8RF
159	Kings Health Partners Joint Clinical Trials Office,<br>Floor 16, Tower Wing,<br>Guy's Hospital,<br>Great Maze Pond,<br>London SE1 9RT<br><br>NRES Committee London ¿ South East Room<br>4W/10, 4th Floor West,<br>Charing Cross Hospital,<br>Fulham Palace Road,<br>London, W6 8RF
166	Imperial College London and Imperial College<br>Healthcare NHS Trust AHSC Joint Research Office,<br>Room GM14,<br>St.Mary 's Hospital, Faculty of Medicine<br>Ground Mezzanine Floor,<br>Praed Street Wing,<br>London W2 1PG<br><br>NRES Committee London ¿ South East Room<br>4W/10, 4th Floor West,<br>Charing Cross Hospital,<br>Fulham Palace Road,<br>London, W6 8RF

090177e184765be1\Approved\Approved On: 16-Aug-2013 07:04

Site number	Name and Address of Committee
**United Kingdom**	
168	Northumberland, Tyne and Wear NHS Trust<br>Research Department,<br>St Nicholas Hospital,<br>Jubilee Road,<br>Gosforth,<br>Newcastle upon Tyne NE3 3XT<br><br>NRES Committee London ¿ South East Room<br>4W/10, 4th Floor West,<br>Charing Cross Hospital,<br>Fulham Palace Road,<br>London, W6 8RF<br><br>The Newcastle upon Tyne Hospitals NHS Foundation<br>Trust Royal Victoria Infirmary,<br>Queen Victoria Road,<br>Newcastle upon Tyne NE1 4LP
175	Northampton General Hopsital NHS Trust Research<br>& Development Centre,<br>Cliftonville,<br>Northampton NN1 5BD<br><br>NRES Committee London ¿ South East Room<br>4W/10, 4th Floor West,<br>Charing Cross Hospital,<br>Fulham Palace Road,<br>London, W6 8RF

090177e184765be1\Approved\Approved On: 16-Aug-2013 07:04

Site number	Name and Address of Committee
**United States**	
216<br>217<br>218<br>219<br>220<br>221<br>222<br>223<br>224<br>227<br>228<br>229<br>230<br>232<br>233<br>235<br>236<br>237<br>238<br>239<br>241<br>242<br>243<br>247<br>249<br>250<br>251<br>252<br>253<br>254<br>255<br>256<br>258<br>260<br>279<br>280<br>286<br>289<br>307	Western Institutional Review Board 3535 7th Ave. SW,<br>Olympia, WA 98502

090177e184765be1\Approved\Approved On: 16-Aug-2013 07:04

Site number	Name and Address of Committee
**United States**	
225	University of Kansas Medical Center Human Subjects Committee,<br>3901 Rainbow Boulevard,<br>Kansas City, KS 66160
240	Springfield Committee for Research Involving Human Subjects P.O. Box 19616<br>801 North Rutledge Street,<br>Springfield, IL 62702
244	Greenwich Hosptial Institutional Review Board 5 Perryridge Road,<br>Greenwich, CT 06830
245	Abington Memorial Hospital Institutional Review Board,<br>1200 Old York Road,<br>Abington, PA 19001-3788
246	Providence Health & Services Institutional Review Board,<br>Building A,<br>5251 NE Glisan Street,<br>3rd Floor,<br>Portland, OR 97213
259	Human Investigation Committee 55 College Street<br>New Haven, CT 06510
261	Michigan State<br>University Biomedical and Health IRB,<br>207 Olds Hall,<br>East Lansing, MI 48824
262	University of Michigan Medical School Institutional Review Board,<br>Building 200 - Room 2086<br>2800 Plymouth Road,<br>Ann Arbor, MI 48109

090177e184765be1\Approved\Approved On: 16-Aug-2013 07:04

Site number	Name and Address of Committee
**United States**	
263	Thomas Jefferson University Office of Scientific Affairs,<br>Division of Human Subjects Protection<br>Suite 1100<br>1015 Chestnut Street,<br>Philadelphia, PA 19107
264	Medical University of South Carolina Office of Research Integrity,<br>19 Hagood Avenue, Suite 601<br>MSC857<br>Charleston, SC 29425
268	University of Pennsylvania 19<br>Hagood Avenue,<br>Suite 601
269	Western Institutional Review Board<br>MSC857
271	Butler Hospital IRB Charleston,<br>SC 29425
355	Partners Research Committee Suite 1002<br>116 Huntington Avenue,<br>Boston, MA 02116

090177e184765be1\Approved\Approved On: 16-Aug-2013 07:04
