# Supplementary material for: Bapineuzumab for mild to moderate Alzheimer’s disease in two global, randomized, phase 3 trials
Source: Alzheimers Res Ther. 2016 May 12;8:18. doi: 10.1186/s13195-016-0189-7 (PMC4866415; doi:10.1186/s13195-016-0189-7)
Supplement: Additional file 6: — List of independent ethics committees or institutional review boards. Complete list of independent ethics committees for all sites that screened subjects for the ApoE ε4 noncarrier study. (PDF 339 kb) [file 13195_2016_189_MOESM6_ESM.pdf]

16.1.3 List of Independent Ethics Committees or Institutional Review Boards

A list of the following is included:

- The List of Independent Ethics Committees, sorted by country and site, for sites that screened subjects.

090177e184835a0b\Approved\Approved On: 07-Sep-2013 01:35

Independent Ethics Committees

Site Number	Name and Address of Committee
**Argentina**	
169	Comite de Etica de la Investigacion del CEMIC<br>Galvan 4102<br>Buenos Aires C1431FWO
173	Comite de Etica de Protocolos de Investigacion (C.E.P.I.)<br>Hospital Italiano<br>Gascon 450 (C1181ACH)<br>Buenos Aires
**Australia**	
52	The Queen Elizabeth Hospital Clinical Pharmacology Laboratory<br>28 Woodville Road<br>Woodville, SA 5011<br><br>Northern Sydney / Central Coast Area Health Service HREC<br>Human Research Ethics Committee, Royal North Shore Hospital<br>Pacific Hwy, St Leonards, NSW 2065<br><br>Human Research Ethics Committee<br>The Queen Elizabeth Hospital<br>28 Woodville Road<br>Woodville South<br>South Australia 5011<br><br>Northern Sydney / Central Coast Area Health Service HREC<br>Human Research Ethics Committee, Royal North Shore Hospital<br>Level 2 Building 51<br>Pacific Hwy<br>St Leonards, NSW 2065

090177e184835a0b\Approved\Approved On: 07-Sep-2013 01:35

Site Number	Name and Address of Committee
**Australia**	
53	Austin Health Human Research Ethics Committee<br>145 Studley Road<br>Heidelberg, Vic 3084
55	Hollywood Private Hospital Research Ethics Committee<br>Monash Avenue,<br>Nedlands, WA 6009
56	Northern Sydney / Central Coast Area Health Service HREC<br>Human Research Ethics Committee, Royal North Shore Hospital<br>Pacific Hwy<br>St Leonards, NSW 2065
57	Central Northern Adelaide Health Service Ethics of Human Research Committee (TQEH & LMH)<br>The Queen Elizabeth Hospital<br>28 Woodville Road<br>Woodville South, SA 5011
58	Royal Adelaide Hospital Research Ethics Committee<br>Royal Adelaide Hospital<br>Level 3, Hanson Centre<br>North Terrace<br>Adelaide, SA 5000
267	BHS & SJOG Human Research Ethics Committee<br>PO Box 577<br>Drummond Street North Ballarat, VIC 3353

090177e184835a0b\Approved\Approved On: 07-Sep-2013 01:35

Site Number	Name and Address of Committee
**Belgium**	
46<br>47<br>48<br>49<br>265<br>272	Commissie Medische Ethiek van de Universitaire Ziekenhuizen KU<br>Campus Gasthuisberg E330<br>Herestraat 49<br>Leuven 3000
**Canada**	
293	Sunnybrook Research Ethics Board<br>Research Ethics Office<br>2075 Bayview Avenue<br>Toronto, ON M4N 3M5
**Canada**	
295<br>296<br>321<br>329<br>373	IRB Services<br>Suite 300<br>372 Hollandview Trail<br>Aurora, ON L4G 0A5
328	Bruyere Continuing Care Research Ethics Board<br>43 Bruyere Street<br>Ottawa, ON K1N 5C8
330	Comite D'Ethique de la Recherche de l'Hopital Maisonneuve-Rosemont<br>5415 Boulevard De l'Assomption<br>Montreal, QC H1T 2M4

090177e184835a0b\Approved\Approved On: 07-Sep-2013 01:35

Site Number	Name and Address of Committee
**Chile**	
126<br>176	Comite Etico Cientifico, Servicio de Salud Metropolitano Oriente<br>Av. Salvador 364<br>Providencia Santiago, RM 7500922
**Croatia**	
43<br>248	Central Ethics Committee<br>Agency for Medicinal Products and Medical Devices<br>Ksaverska c. 4<br>Zagreb 10000
**Finland**	
3<br>6	Pohjois-Savon sairaanhoitopiirin ky<br>Tutkimuseettinen toimikunta<br>Rakennus 10 (4. krs)<br>PL 1777Kuopio 70211

090177e184835a0b\Approved\Approved On: 07-Sep-2013 01:35

Site Number	Name and Address of Committee
**France**	
109<br>110<br>111<br>112<br>113<br>114<br>115<br>117<br>118<br>119<br>120<br>121<br>122<br>123<br>124<br>125<br>157<br>158<br>291	CPP Ile de France 3<br>Hôpital TARNIER<br>89, rue dAssas<br>PARIS 75006
**Germany**	
96<br>101<br>292<br>315	Ethik-Kommission der Fakultaet fuer Medizin der Technischen Universitaet Muenchen<br>Ismaninger Str. 22<br>Muenchen 81675

090177e184835a0b\Approved\Approved On: 07-Sep-2013 01:35

Site Number	Name and Address of Committee
**Italy**	
29	Comitato Etico Indipendente<br>dell'IRCCS Fondazione S. Lucia di Roma<br>Via Ardeatina, 306, Roma 00179
31	Comitato Etico dell'IRCCS Fondazione San Raffaele del Monte Tabor di Milano<br>Via Olgettina, 60 Milano 20132
33	Comitato Etico<br>della Fondazione IRCCS Istituto Neurologico Carlo Besta di Milano<br>Via Celoria, 11 Milano 20133
34	Comitato Etico dell'Azienda Ospedaliero Universitaria<br>Ospedali Riuniti Umberto I - G.M. Lancisi - G. Salesi di ancona<br>Via Conca, 71<br>Ancona 60126
36	Comitato Etico per la Sperimentazione Clinica dei Medicinali<br>dell'Azienda Ospedaliero-Universitaria Careggi di Firenze<br>C.T.O.<br>Largo Palagi, 1<br>Firenze 50139
37	Comitato Etico Locale per la Sperimentazione Clinica dei Medicinali<br>dell'Azienda Ospedaliera Universitaria Senese di Siena<br>c/o UOC Farmacia AOUS<br>Viale Bracci<br>Siena 53100

090177e184835a0b\Approved\Approved On: 07-Sep-2013 01:35

Site Number	Name and Address of Committee
**Italy**	
38	Comitato Etico dell'Universita' degli Studi Gabriele D'Annunzio<br>e della ASL 2 Lanciano-Vasto-Chieti di Chieti<br>Via dei Vestini, 31<br>Chieti 66100
39	Comitato Etico Azienda Ospedaliera-Spedali Civili di Brescia<br>P.le Spedali Civili 1<br>Brescia 25123
40	Comitato Etico Dell'azienda Ospedaliera Cannizzaro Di Catania<br>Via messina, 829<br>Catania 95126
90	Comitato Etico Azienda Ospedaliera San Gerardo di Monza<br>Via Pergolesi, 33<br>Monza (MI) 20052
211	Comitato Etico dell'Universita' Campus Bio-Medico di Roma<br>Via Alvaro del Portilllo, 21<br>Roma 00128
**Japan**	
177	National Hospital Organization Kokura Medical Center<br>10-1 Harugaoka Kokuraminami-ku<br>Kitakyusyu-shiFukuoka 802-8533
178	Yokohama City University Medical Center IRB<br>4-57 Urafunecho Minami-kuYokohama, Kanagawa 232-0024

090177e184835a0b\Approved\Approved On: 07-Sep-2013 01:35

Site Number	Name and Address of Committee
**Japan**	
179	National Hospital Organization Minami-Okayama Medical Center IRB<br>4066 Hayashima, Hayashima-cho, Tsukubo-gun<br>Okayama 701-0304
180	Juntendo University Hospital IRB<br>3-1-3 HongoBunkyo-ku, Tokyo 113-8431
181	National Hospital Organization Niigata National Hospital IRB<br>3-52 Akasaka-cho kashiwazaki, Niigata 945-8585
182	National Hospital Organization Chiba-East Hospital<br>673 Nitona-chou Chuou-kuChiba, Chiba 260-8712
183	Kagawa University Hospital IRB<br>1750-1 Ikenobe Miki-choKita-gun, Kagawa 761-0793
184	National Hospital Organization Shizuoka Institute of Epilepsy and Neurological Disorders<br>886 Urushiyama Aoi-kuShizuoka, Shizuoka 420-8688
185	Osaka City University Hospital<br>1-5-7, Asahi-machi, Abeno-ku,Osaka, Osaka, 545-8586
186	Juntendo Tokyo Koto Geriatric Medical Center IRB<br>3-3-20, Shinsuna<br>Koto-ku, Tokyo 136-0075
187	Tokyo Medical University Hospital<br>6-7-1 Nishi-ShinjukuShinjuku-ku, Tokyo 160-0023
188	Rakuwakai Otowa Hospital IRB<br>2 Chinji-cho, Otowa,<br>Yamashina-kuKyoto-shi, Kyoto 607-8062

090177e184835a0b\Approved\Approved On: 07-Sep-2013 01:35

Site Number	Name and Address of Committee
**Japan**	
189	Okayama University Hospital IRB<br>2-5-1 Shikata-cho, Kita-ku Okayama, Okayama 700-8558
190	National Hospital Organization Minami-Kyoto Hospital IRB<br>11 Ashihara, Naka,Jouyou-shi, Kyoto 610-0113
191	National Hospital Organization Hiroshima-nishi Medical Center<br>4-1-1 KubaOtake city, Hiroshima 739-0696
192	National Hospital Organization Tokyo National Hospital IRB<br>3-1-1 Takeoka, Kiyose-shi, Tokyo 204-8585
193	National Hospital Organization Maizuru Medical Center IRB<br>2410 Aza Yukinaga, Maizuru, Kyoto 625-8502
194	Kansai Medical University Takii Hospital<br>10-15 Fujizono-cho Moriguchi, Osaka 570-8507
195	Gunma University Hospital<br>3-39-15 Showa-machiMaebashi, Gunma 371-8511
196	Kobe University Hospital IRB<br>7-5-2 kusunoki-cho, Chuo-kuKobe, Hyogo 650-0017
197	Nagoya City University Hospital IRB<br>1 Kawasumi Mizuho-cho, Mizuho-ku,<br>Nagoya, Aichi, 467-8602
198	Osaka University Hospital IRB<br>Institutional Review Board<br>2-15 YamadaokaSuita, Osaka 565-0871
200	Tokyo Medical University Hachioji Medical Center<br>1163 TatemachiHachioji, Tokyo 193-0944

090177e184835a0b\Approved\Approved On: 07-Sep-2013 01:35

Site Number	Name and Address of Committee
**Japan**	
201	Tokyo Metropolitan Health and Medical Treatment Corporation Ebara Hospital<br>4-5-10 Higashi-YukigayaOta-ku, Tokyo 145-0065
202	Nippon Medical School Chiba Hokusoh Hospital IRB<br>1715, Kamagari, Inzaishi Chiba 270-1694
203	Maebashi Red Cross Hospital IRB<br>3-21-36 ASAHI CHOUMAEBASHI, GUNMA 371-0014
204	Juntendo University Hospital IRB<br>3-1-3 HongoBunkyo-ku, Tokyo 113-8431
207	Iwate Medical University Hospital IRB<br>19-1 UchimaruMorioka-shi, Iwate 020-8505
208	Yachiyo Hospital<br>2-2-7 Sumiyoshi-cho, Anjo-city, Aichi 446-8510
274	National Hospital Organization Matsumoto Medical Center IRB<br>811, Kotobukitoyooka, Matsumoto, Nagano 399-0021
275	Tokusyukai Group IRB<br>1-8-7 KojimachiChiyoda-ku, Tokyo 102-0083
276	Nippon Medical School Musashi Kosugi Hospital IRB<br>1-396, Kosugimachi, Nakaharaku,<br>kawasaki, kanagawa 211-8533
277	Shinozuka Hospital IRB<br>105-1 Shinozuka, Fujioka-city, Gunma 375-0017
281	Kashiwado Hospital IRB<br>2-21-8 Nagazu, Chuo-ku, Chiba-shi, Chiba 260-8656

090177e184835a0b\Approved\Approved On: 07-Sep-2013 01:35

Site Number	Name and Address of Committee
**Japan**	
308	Himorogi Psychiatric Institute IRB<br>1-20-10 SugamoToshima-ku, Tokyo 170-0002
309	Kobe City Hospital Organization Kobe City Medical Center West Hospital IRB<br>2-4 Ichiban-cho, Ngata-ku, kobe city, Hyogo 653-0013
**Korea**	
472	Seoul National University Bundang Hospital IRB<br>166 gumi-ro, Bundang-gu,<br>Seongnam-si, Gyeonggi-do 463-707
474	Institutional Review Board for Human Research, Konkuk University Medical Center<br>4-12, Hwayang-dong, Gwangjin-gu Seoul 143-729
**Mexico**	
154	Comite de Etica, Investigacion y Bioseguridad Privada de Aguascalientes, SC<br>Sierra Fria 218<br>Fraccionamiento Bosques del Prado NorteAguascalientes, Aguascalientes 20127
283	Hospital y Clinical OCA<br>Pino Suarez 640 Nte. Colonia Centro<br>Monterrey, Nuevo Leon 64000

090177e184835a0b\Approved\Approved On: 07-Sep-2013 01:35

Site Number	Name and Address of Committee
**Netherlands**	
70<br>72<br>77<br>80<br>160<br>226<br>285	Vrije Universiteit Medisch Centrum<br>Medisch Ethische Toetsingscommissie<br>Postbus 7057<br>Amsterdam, NH 1007 MB
**New Zealand**	
54<br>107	Multi Region Ethics Committee<br>Ministry of Health<br>PO Box 5013<br>Wellington
**Poland**	
143<br>146<br>147<br>150<br>282<br>306	Komisja Bioetyczna przy Warszawskim Uniwersytecie Medycznym w Warszawie<br>ul. Zwirki i Wigury 61<br>Warszawa 02-091
**Portugal**	
161<br>162<br>163	Comissao de Etica Para a Investigaçao Clinica<br>PARQUE DA SAUDE DE LISBOA.<br>AV. DO BRASIL, 53 PAV. 17-A<br>LISBOA, LISBOA 1749-004

090177e184835a0b\Approved\Approved On: 07-Sep-2013 01:35

Site Number	Name and Address of Committee
**Russia**	
298	Local Ethics Committee<br>GU St-Petersburg scientific-research psychoneurological institute named after V.M. Bekhterev<br>of Roszdrav<br>3 ulitsa Bekhtereva<br>Saint-Petersburg 192019<br><br>Ethics Committee Affiliated<br>with the Federal Services on Surveillance in Healthcare and Social Development<br>Building 2, No. 8 Petrovskiy Boulevard<br>Moscow 127051<br><br>State Institution St-Petersburg scientific research psychoneurological V.M. Bekhterev of Roszdrav<br>3, Bekhtereva ul<br>Saint Petersburg 192019<br><br>Ethical council under responsibility of department of state regulation of circulation of medicines<br>Ministry of public health and social development of Russian federation<br>3, Rakhmanovkiy per. City service post office<br>4 Moscow, 127994<br><br>Ethics Council at the Ministry of Healthcare of Russian Federation<br>3, Rakhmanovskij per<br>Moscow 127994.

090177e184835a0b\Approved\Approved On: 07-Sep-2013 01:35

Site Number	Name and Address of Committee
**Russia**	
300	Local Ethics Committee at Scientific Research Medical Complex Vashe Zdorovie<br>7 ulitsa Zinina<br>Kazan 420097<br><br>Ethics Council at the Ministry of Healthcare of Russian Federation<br>3, Rakhmanovskij per.<br>Moscow 127994
301	Local Ethics Committee<br>GU St-Petersburg scientific-research psychoneurological institute named after V.M. Bekhterev of Roszdrav<br>3 ulitsa Bekhtereva<br>Saint-Petersburg 192019<br><br>Ethics Committee Affiliated<br>Ethics Committee Affiliated with the Federal Services on Surveillance<br>in Healthcare and Social Development<br>Building 2, No. 8 Petrovskiy Boulevard<br>Moscow 127051<br><br>Ethics Council at the Ministry of Healthcare of Russian Federation<br>3, Rakhmanovskij per.<br>Moscow 127994

090177e184835a0b\Approved\Approved On: 07-Sep-2013 01:35

Site Number	Name and Address of Committee
**Russia**	
302	Local Ethics Committee at Military medical academy n.a. S.M. Kirov<br>6 ulitsa Akademika Lebedeva<br>Saint-Petersburg 194044<br><br>LEC Mailitary medical academy n.a. S.M. Kirov of Ministry of Defense of Russia<br>6, akad Lebedeva ulSt Petersburg 194044<br><br>Ethics Council at the Ministry of Healthcare of Russian Federation<br>3, Rakhmanovskij per.<br>Moscow 127994<br><br>Ethics Committee Affiliated<br>Ethics Committee Affiliated with the Federal Services on Surveillance<br>in Healthcare and Social Development<br>Building 2, No. 8 Petrovskiy Boulevard<br>Moscow 127051

090177e184835a0b\Approved\Approved On: 07-Sep-2013 01:35

Site Number	Name and Address of Committee
**Russia**	
303	Local Ethics Committee at City geriatric medical social center<br>148 nab reki Fontanki<br>Saint-Petersburg 190103<br><br>GUZ Cite Geriatric medical-social center<br>148, Fontanka emb<br>St Petersburg 198103<br><br>Ethics Committee Affiliated<br>Ethics Committee Affiliated with the Federal Services on Surveillance<br>in Healthcare and Social Development<br>Building 2, No. 8 Petrovskiy Boulevard<br>Moscow 127051<br><br>Ethics Council at the Ministry of Healthcare of Russian Federation<br>3, Rakhmanovskij per.<br>Moscow 127994

090177e184835a0b\Approved\Approved On: 07-Sep-2013 01:35

Site Number	Name and Address of Committee
**Russia**	
304	Local Ethics Committee at Nizhny Novgorod regional clinical hospital n.a. N.A.Semashko<br>190 ulitsa Rodionova<br>Nizhny Novgorod 603126<br><br>Ethics Council at the Ministry of Healthcare of Russian Federation<br>3, Rakhmanovskij per.<br>Moscow 127994<br><br>Ethics Committee Affiliated<br>Ethics Committee Affiliated with the Federal Services on Surveillance<br>in Healthcare and Social Development<br>Building 2, No. 8 Petrovskiy Boulevard<br>Moscow 127051
**Serbia**	
42	Ethics Committee of CCS<br>Pasterova 2<br>Belgrade 11000
44	Ethics Committee of CC Kragujevac<br>Zmaj Jovina 32<br>Kragujevac, Serbia 34000
266	Ethics Committee<br>Clinical Centre of Vojvodina<br>Hajduk Veljkova 1-9<br>Novi Sad 21000

090177e184835a0b\Approved\Approved On: 07-Sep-2013 01:35

Site Number	Name and Address of Committee
**Slovakia**	
135	Eticka komisia Vseobecnej nemocnice Rimavska Sobota<br>Srobarova 1<br>Rimavska Sobota 979 12
136<br>310	Eticka komisia Vseobecnej nemocnice Rimavska Sobota<br>Srobarova 1<br>Rimavska Sobota 979 12<br>Slovakia<br><br>Eticka komisia UN Bratislava<br>Nemocnica Ruzinov<br>Ruzinovska 6<br>Bratislava 826 06
137	Eticka komisia Vseobecnej nemocnice Rimavska Sobota<br>Srobarova 1<br>Rimavska Sobota 979 12<br><br>Eticka komisia<br>Psychiatricka nemocnica Michalovce, n.o.<br>Stranany<br>Michalovce 071 01
138	Eticka komisia Bratislavskeho samospravneho kraja<br>Sabinovska 16<br>P.O.Box 106<br>Bratislava 820 05

090177e184835a0b\Approved\Approved On: 07-Sep-2013 01:35

Site Number	Name and Address of Committee
**Slovakia**	
311	Eticka komisia Vseobecnej nemocnice Rimavska Sobota<br>Srobarova 1 Rimavska Sobota 979 12<br><br>Eticka komisia<br>Univerzitna nemocnica Martin<br>Kollarova 2<br>Martin, Slovakia 036 59
313	Eticka komisia Vseobecnej nemocnice Rimavska Sobota<br>Srobarova 1 Rimavska Sobota 979 12<br><br>Eticka komisia UN Bratislava a LF UK<br>Nemocnica Stare Mesto<br>Mickiewiczova 13Bratislava 813 69
314	Eticka komisia Vseobecnej nemocnice Rimavska Sobota<br>Srobarova 1 Rimavska Sobota 979 12<br><br>Eticka komisia<br>Fakultna nemocnica s poliklinikou Zilina<br>Vojtecha Spanyola 43<br>Zilina 012 07
**South Africa**	
83<br>84<br>86<br>89	Pharma Ethics<br>123 Amcor Road<br>Lyttelton Manor,<br>Centurion 0157

090177e184835a0b\Approved\Approved On: 07-Sep-2013 01:35

Site Number	Name and Address of Committee
**Spain**	
88	Pharma Ethics (Pty) Ltd<br>Lyttleton Manor<br>123 Amcor Road<br>Pretoria 0157
14	Hospital Clinic i Provincial de Barcelona<br>Comite Etico de Investigacion Clinica<br>Villarroel, 170<br>Barcelona, Barcelona 08036<br><br>COMITE ETICO DE INVESTIGACION CLINICA Parc de Salut MAR, COMITE ETICO DE INVESTIGACION CLINICA, EDIFICIO PRBB<br>C/ DOCTOR AIGUADER, Nº 88<br>BARCELONA, BARCELONA 08003
15	COMITE ETICO DE INVESTIGACION CLINICA Parc de Salut MAR<br>COMITE ETICO DE INVESTIGACION CLINICA<br>EDIFICIO PRBB<br>C/ DOCTOR AIGUADER, Nº 88<br>BARCELONA, BARCELONA 08003<br><br>CEIC Parc de Salut Mar<br>Comite Etico de Investigacion Clinica<br>Secretaria Tecnica. Parc de Recerca Biomedica de Barcelona (Dcho. 163.03)<br>C/ Doctor Aiguader, 88<br>Barcelona, Barcelona 08003

090177e184835a0b\Approved\Approved On: 07-Sep-2013 01:35

Site Number	Name and Address of Committee
**Spain**	
16	Hospital de la Santa Creu i Sant Pau<br>COMITE ETICO DE INVESTIGACION CLINICA<br>AVDA. SANT ANTONI MARIA CLARET, 167<br>BARCELONA, BARCELONA 08025<br><br>COMITE ETICO DE INVESTIGACION CLINICA Parc de Salut MAR<br>COMITE ETICO DE INVESTIGACION CLINICA<br>EDIFICIO PRBB<br>C/ DOCTOR AIGUADER, Nº 88<br>BARCELONA, BARCELONA 08003
18	Hospital Mutua de Terrassa<br>Comite Etico de Investigacion Clinica<br>Pl. Dr. Robert, 5<br>Terrassa, Barcelona 08221<br><br>COMITE ETICO DE INVESTIGACION CLINICA Parc de Salut MAR<br>COMITE ETICO DE INVESTIGACION CLINICA<br>EDIFICIO PRBB<br>C/ DOCTOR AIGUADER, Nº 88<br>BARCELONA, BARCELONA 08003

090177e184835a0b\Approved\Approved On: 07-Sep-2013 01:35

Site Number	Name and Address of Committee
**Spain**	
19	Hospital Universitario La Paz<br>Comite Etico de Investigacion Clinica<br>Hospital General Planta 8ª<br>Paseo de la Castellana, 261<br>Madrid, Madrid 28046<br><br>COMITE ETICO DE INVESTIGACION CLINICA Parc de Salut MAR<br>COMITE ETICO DE INVESTIGACION CLINICA<br>EDIFICIO PRBB<br>C/ DOCTOR AIGUADER, Nº 88<br>BARCELONA, BARCELONA 08003
20	COMITE ETICO DE INVESTIGACION CLINICA Parc de Salut MAR<br>COMITE ETICO DE INVESTIGACION CLINICA<br>EDIFICIO PRBB, C/ DOCTOR AIGUADER, Nº 88<br>BARCELONA, BARCELONA 08003<br><br>CEIC DE BURGOS Y SORIA, Hospital General Yague<br>Avda del Cid, 96BURGOS 09005

090177e184835a0b\Approved\Approved On: 07-Sep-2013 01:35

Site Number	Name and Address of Committee
**Spain**	
21	Hospital 12 de Octubre<br>Instituto de Investigacion Hospital 12 de Octubre (i+12)<br>Area de Gestion de Proyectos - Unidad Administrativa CEIC<br>Centro de Actividades Ambulatorias, Bloque D - Planta 6ª<br>Avda de Cordoba s/nMadrid, Madrid 28041<br><br>COMITE ETICO DE INVESTIGACION CLINICA Parc de Salut MAR<br>COMITE ETICO DE INVESTIGACION CLINICA<br>EDIFICIO PRBB<br>C/ DOCTOR AIGUADER, Nº 88<br>BARCELONA, BARCELONA 08003
22	Hospital de Cruces<br>Comite Etico de Investigacion Clinica<br>Plaza de Cruces, s/n<br>Barakaldo, Vizcaya 48903<br><br>COMITE ETICO DE INVESTIGACION CLINICA Parc de Salut MAR<br>COMITE ETICO DE INVESTIGACION CLINICA<br>EDIFICIO PRBB<br>C/ DOCTOR AIGUADER, Nº 88<br>BARCELONA, BARCELONA 08003

090177e184835a0b\Approved\Approved On: 07-Sep-2013 01:35

Site Number	Name and Address of Committee
**Spain**	
23	Hospital Clinico San Carlos<br>Comite Etico de Investigacion Clinica<br>Ciudad Universitaria. Planta 1ª - Ala Norte - Puerta G<br>C/ Doctor Martin Lagos s/n<br>Madrid, Madrid 28040<br><br>COMITE ETICO DE INVESTIGACION CLINICA Parc de Salut MAR<br>COMITE ETICO DE INVESTIGACION CLINICA<br>EDIFICIO PRBB<br>C/ DOCTOR AIGUADER, Nº 88<br>BARCELONA, BARCELONA 08003
24	Hospital Universitario de la Princesa<br>Comite Etico de Investigacion Clinica<br>C/ Diego de Leon 62<br>Madrid 28006<br><br>COMITE ETICO DE INVESTIGACION CLINICA Parc de Salut MAR<br>COMITE ETICO DE INVESTIGACION CLINICA<br>EDIFICIO PRBB<br>C/ DOCTOR AIGUADER, Nº 88<br>BARCELONA, BARCELONA 08003

090177e184835a0b\Approved\Approved On: 07-Sep-2013 01:35

Site Number	Name and Address of Committee
**Spain**	
25	Hospital General de Elche<br>Comite Etico de Investigacion Clinica<br>Cami de l'Almazara, 11<br>Elche, Alicante 03203<br><br>COMITE ETICO DE INVESTIGACION CLINICA Parc de Salut MAR<br>COMITE ETICO DE INVESTIGACION CLINICA<br>EDIFICIO PRBB<br>C/ DOCTOR AIGUADER, Nº 88<br>BARCELONA, BARCELONA 08003
26	COMITE ETICO DE INVESTIGACION CLINICA Parc de Salut MAR<br>COMITE ETICO DE INVESTIGACION CLINICA<br>EDIFICIO PRBB<br>C/ DOCTOR AIGUADER, Nº 88<br>BARCELONA, BARCELONA 08003

090177e184835a0b\Approved\Approved On: 07-Sep-2013 01:35

Site Number	Name and Address of Committee
**Spain**	
51	Complejo Hospitalario de Caceres<br>Comite Etico de Investigacion Clinica<br>Avda. Pablo Naranjo, s/n<br>Caceres, Caceres 10003<br><br>COMITE ETICO DE INVESTIGACION CLINICA Parc de Salut MAR<br>COMITE ETICO DE INVESTIGACION CLINICA<br>EDIFICIO PRBB<br>C/ DOCTOR AIGUADER, Nº 88<br>BARCELONA, BARCELONA 08003
212	Hospital Universitario Ramon y Cajal<br>Comite Etico de Investigacion Clinica<br>Secretaria del Comite - Planta 2ª Dcha.<br>Ctra. de Colmenar Viejo, Km. 9.100<br>Madrid, Madrid 28034<br><br>COMITE ETICO DE INVESTIGACION CLINICA Parc de Salut MAR<br>COMITE ETICO DE INVESTIGACION CLINICA<br>EDIFICIO PRBB<br>C/ DOCTOR AIGUADER, Nº 88<br>BARCELONA, BARCELONA 08003

090177e184835a0b\Approved\Approved On: 07-Sep-2013 01:35

Site Number	Name and Address of Committee
**Spain**	
214	COMITE ETICO DE INVESTIGACION CLINICA Parc de Salut MAR<br>COMITE ETICO DE INVESTIGACION CLINICA<br>EDIFICIO PRBB<br>C/ DOCTOR AIGUADER, Nº 88<br>BARCELONA, BARCELONA 08003<br><br>Comite Etico de Investigacion Clinica de Las Islas Baleares<br>Conselleria de Salut i Consum<br>Camino de Jesus, 38<br>Palma de Mallorca, Islas Baleares 07011
**Sweden**	
005<br>215	Regionala etikprovningsnamnden i Stockholm<br>FE 289<br>Stockholm 171 77
**Switzerland**	
127	Ethikkommission beider Basel EKBB<br>Hebelstrasse 53 Basel CH-4056
128	University Hospital Geneva<br>Ethic Committee of Internal Medicine<br>Geneva 1211
164	Commission d'Ethique de la Recherche Clinique de la faculte de Biologie et Medecine<br>Faculte de Biologie et de Medecine<br>Decanat Rue du Bugnon 21Lausanne 1005

090177e184835a0b\Approved\Approved On: 07-Sep-2013 01:35

Site Number	Name and Address of Committee
**United Kingdom**	
60	NRES Committee London-South East<br>South East Coast Strategic Health Authority<br>Preston Hall<br>Aylesford, Kent ME20 7NJ<br><br>Sheffield Health and Social Care NHS<br>Research Development Unit<br>Fulwood House<br>Old Fulwood Road<br>Sheffield S10 3TH<br><br>NRES Committee London South East<br>Room 4W/10, 4th Floor West<br>Charing Cross Hospital<br>Fulham Palace Road<br>London, UK W6 8RF<br><br>STH NHS Foundation Trust Research Department<br>1st Floor<br>11 Broomfield Road<br>Sheffield S10 2SE

090177e184835a0b\Approved\Approved On: 07-Sep-2013 01:35

Site Number	Name and Address of Committee
**United Kingdom**	
61	NRES Committee London South East<br>Room 4W/10, 4th Floor West<br>Charing Cross Hospital<br>Fulham Palace Road<br>London, UK W6 8RF<br><br>NRES Committee London-South East<br>South East Coast Strategic Health Authority<br>Preston Hall<br>Aylesford, Kent ME20 7NJ<br><br>Cardiff and Vale University Health Board<br>University Hospital of Wales<br>Commercial Clinical Trials, Research & Development Office<br>Second Floor, Tower Block 2, Room 3 (2TB2 R3)<br>Heath ParkCardiff CF14 4XW

090177e184835a0b\Approved\Approved On: 07-Sep-2013 01:35

Site Number	Name and Address of Committee
**United Kingdom**	
62	NRES Committee London South East<br>Room 4W/10, 4th Floor West<br>Charing Cross Hospital, Fulham Palace Road<br>London, UK W6 8RF<br><br>NRES Committee London-South East<br>South East Coast Strategic Health Authority<br>Preston Hall<br>Aylesford, Kent ME20 7NJ<br><br>Research & Development Directorate, Brighton & Sussex University Hospitals NHS Trust, Clinical Investigaton & Research Unit, Royal Sussex County Hospital, Level 5 Thomas Kemp Tower<br>Eastern Road Brighton BN25BE

090177e184835a0b\Approved\Approved On: 07-Sep-2013 01:35

Site Number	Name and Address of Committee
**United Kingdom**	
65	NRES Committee London South East<br>Room 4W/10, 4th Floor West<br>Charing Cross Hospital<br>Fulham Palace Road<br>London, UK W6 8RF<br><br>NRES Committee London-South East<br>South East Coast Strategic Health Authority<br>Preston Hall<br>Aylesford, Kent ME20 7NJ<br><br>NRES Committee London South East<br>Health Research Authority<br>Ground Floor, Skipton House<br>80 London Road<br>London SE1 6LH<br><br>Avon and Wiltshire Mental Health Partnership NHS Trust<br>Research and Development<br>The Blackberry Centre<br>Blackberry Hill Hospital<br>Manor Road, Fishponds Bristol BS16 2EW

090177e184835a0b\Approved\Approved On: 07-Sep-2013 01:35

Site Number	Name and Address of Committee
**United Kingdom**	
66	NRES Committee London South East<br>Room 4W/10, 4th Floor West<br>Charing Cross Hospital<br>Fulham Palace Road<br>London, UK W6 8RF<br><br>NRES Committee London-South East<br>South East Coast Strategic Health Authority<br>Preston Hall<br>Aylesford, Kent ME20 7NJ<br><br>NHS Greater Glasgow and Community Primary Care, Community & Mental Health LREC<br>South Glasgow & Clyde REC<br>R&D Directorate, 1st Floor - The Tennent Institute<br>Western Infirmary<br>38 Church Street Glasgow G11 6NT
67<br>68	NRES Committee London South East<br>Room 4W/10, 4th Floor West<br>Charing Cross Hospital<br>Fulham Palace Road<br>London, UK W6 8RF<br><br>NRES Committee London-South East<br>South East Coast Strategic Health Authority<br>Preston Hall Aylesford, Kent ME20 7NJ

090177e184835a0b\Approved\Approved On: 07-Sep-2013 01:35

Site Number	Name and Address of Committee
**United Kingdom**	
159	NRES Committee London South East<br>Room 4W/10, 4th Floor West<br>Charing Cross Hospital<br>Fulham Palace Road<br>London, UK W6 8RF<br><br>NRES Committee London-South East<br>South East Coast Strategic Health Authority<br>Preston Hall<br>Aylesford, Kent ME20 7NJ<br><br>Kings Health Partners<br>Joint Clinical Trials Office<br>Floor 16, Tower Wing<br>Guy's Hospital<br>Great Maze Pond London SE1 9RT

090177e184835a0b\Approved\Approved On: 07-Sep-2013 01:35

Site Number	Name and Address of Committee
**United Kingdom**	
166	NRES Committee London South East<br>Room 4W/10, 4th Floor West, Charing Cross Hospital<br>Fulham Palace Road, London, UK W6 8RF<br><br>NRES Committee London-South East<br>South East Coast Strategic Health Authority, Preston Hall, Aylesford, Kent ME20 7NJ<br><br>Imperial College London and Imperial College Healthcare NHS Trust, AHSC Joint Research Office, Room GM14<br>St. Mary 's Hospital, Faculty of Medicine<br>Ground Mezzanine Floor<br>Praed Street Wing London W2 1PG

090177e184835a0b\Approved\Approved On: 07-Sep-2013 01:35

Site Number	Name and Address of Committee
**United Kingdom**	
168	The Newcastle upon Tyne Hospitals NHS Foundation Trust<br>Royal Victoria Infirmary<br>Queen Victoria Road<br>Newcastle upon Tyne NE1 4LP<br><br>NRES Committee London South East<br>Room 4W/10, 4th Floor West<br>Charing Cross Hospital<br>Fulham Palace Road<br>London, UK W6 8RF<br><br>Northumberland, Tyne and Wear NHS Trust<br>Research Department<br>St Nicholas Hospital<br>Jubilee Road<br>GosforthNewcastle upon Tyne NE3 3XT<br><br>NRES Committee London-South East<br>South East Coast Strategic Health Authority<br>Preston Hall<br>Aylesford, Kent ME20 7NJ

090177e184835a0b\Approved\Approved On: 07-Sep-2013 01:35

Site Number	Name and Address of Committee
**United Kingdom**	
175	South East Research Ethics Committee<br>South East Coast Strategic Health Authority<br>Preston Hall<br>Aylesford, Kent ME20 7NJ<br><br>Northampton General Hopsital NHS Trust<br>Research & Development Centre<br>Cliftonville, Northampton NN1 5BD<br><br>NRES Committee London South East<br>Room 4W/10, 4th Floor West<br>Charing Cross Hospital<br>Fulham Palace Road, London, UK W6 8RF

090177e184835a0b\Approved\Approved On: 07-Sep-2013 01:35

Site Number	Name and Address of Committee
**United States**	
216	
217	
218	
219	
220	
221	
223	
224	
227	
228	
229	
230	Western Institutional Review Board
235	3535 Seventh Avenue Southwest
236	Olympia, WA 98502-5010
238	
239	
241	
242	
243	
247	
250	
251	
252	
253	
254	

090177e184835a0b\Approved\Approved On: 07-Sep-2013 01:35

Site Number	Name and Address of Committee
**United States**	
255	
256	
258	
260	
269	
279	
280	
286	
288	
289	
317	
318	Western Institutional Review Board
319	3535 Seventh Avenue Southwest
320	Olympia, WA 98502-5010
322	
325	
326	
327	
332	
333	
334	
357	
359	
363	
370	

090177e184835a0b\Approved\Approved On: 07-Sep-2013 01:35

Site Number	Name and Address of Committee
**United States**	
372<br>396<br>397<br>398<br>400<br>402<br>406<br>407<br>409<br>413<br>414<br>416<br>417<br>418<br>421<br>423<br>432<br>433<br>434<br>445<br>450<br>463<br>465	Western Institutional Review Board<br>3535 Seventh Avenue Southwest<br>Olympia, WA 98502-5010
468<br>469	Western Institutional Review Board<br>3535 Seventh Avenue Southwest<br>Olympia, WA 98502-5010

090177e184835a0b\Approved\Approved On: 07-Sep-2013 01:35

Site Number	Name and Address of Committee
**United States**	
240	Springfield Committee for Research Involving Human Subjects<br>P.O. Box 19616<br>801 North Rutledge<br>Springfield, IL 62794-9616
244	Greenwich Hospital Institutional Review Board<br>Greenwich Hospital<br>5 Perryridge Road<br>Greenwich, CT 06830
245	Abington Memorial Hospital IRB<br>1200 Old York Road<br>Abington, PA 19001
246	Providence Health & Services Institutional Review Board<br>Building A<br>5251 NE Glisan Street<br>3rd Floor Portland, OR 97213
257	Institutional Review Board for Health Sciences Research<br>University of Virginia Health Sciences Center<br>Suite 400<br>One Morton Drive<br>Charlottesville, VA 22903
259	Human Investigation Committee<br>Yale University School of Medicine<br>53-55 College Street<br>New Haven, CT 06520

090177e184835a0b\Approved\Approved On: 07-Sep-2013 01:35

Site Number	Name and Address of Committee
**United States**	
261	Michigan State University<br>Biomedical and Health IRB<br>207 Olds Hall<br>East Lansing, MI 48824
262	University of Michigan Medical School Institutional Review Board (IRBMED)<br>Room 2086<br>2800 Plymouth Road<br>Building 200Ann Arbor, MI 48109-2800
264	Medical University of South Carolina Institutional Review Board for Human Research<br>Office of Research Integrity<br>Suite 601 - MSC 857<br>19 Hagood Avenue<br>Charleston, SC 29425
268	University of Pennsylvania Office of Regulatory Affairs<br>Suite 301S<br>3624 Market Street<br>Philadelphia, PA 19104
271	Butler Hospital Institutional Review Board<br>345 Blackstone Boulevard<br>Providence, RI 02906

090177e184835a0b\Approved\Approved On: 07-Sep-2013 01:35

Site Number	Name and Address of Committee
**United States**	
355	Partners Research Committee<br>Suite 1002<br>116 Huntington Avenue<br>Boston, MA 02116<br><br>PHS Research Management<br>101 Huntington Avenue<br>Boston, MA 02116
358	University of North Texas Health Science Center at Fort Worth IRB<br>3400 Camp Bowie Blvd<br>Forth Worth, TX 76107
365	Sharp Institutional Review Board<br>8695 Spectrum Center Boulevard<br>San Diego, CA 92123
412	Loma Linda University Adventist Health Sciences Center - Institutional Review Board<br>11188 Anderson Street<br>Loma Linda, CA 92350
422	Broward Health IRB<br>1600 South Andrews Avenue<br>Fort Lauderdale, FL 33316

090177e184835a0b\Approved\Approved On: 07-Sep-2013 01:35
